# Chromatin-mediated upregulation of *Monascus* pigments in *Talaromyces purpurogenus* OUCMDZ-019 via disruption of *Ash2*

**DOI:** 10.1080/21501203.2026.2657145

**Published:** 2026-05-04

**Authors:** Chengfeng Hou, Deng Yu, Yuqing Wang, Peng Fu, Weiming Zhu, Yi Wang

**Affiliations:** a School of Medicine and Pharmacy, Ocean University of China, Qingdao, China; b Laboratory for Marine Drugs and Bioproducts, Qingdao Marine Science and Technology Centre, Qingdao, China; c Key Laboratory of Marine Drugs, Ministry of Education of China, School of Medicine and Pharmacy, Ocean University of China, Qingdao, China

**Keywords:** *Talaromyces purpurogenus*, *Monascus* pigments, H3K4 methylation, epigenetic derepression, GNPS molecular networking

## Abstract

*Monascus* pigments (MPs) are important azaphilone-type polyketide pigments, yet yield control, stability, and safety pose challenges. Using an epigenetic derepression strategy, we constructed an *Ash2* deletion mutant in the acidophilic fungus *Talaromyces purpurogenus* OUCMDZ-019, with *Ash2* being a COMPASS subunit involved in H3K4 methylation. Transcriptomic analysis revealed broad transcriptional changes, including marked upregulation of an MPs-related BGC, accompanied by pronounced metabolome remodeling. Integrating genome mining, transcriptomics, and GNPS molecular networking enabled targeted isolation of twelve MP derivatives, including four new azaphilones (talarpurpurones A–D), whose absolute configurations were confirmed by spectroscopy. pH-dependent transformation experiments clarified the behavior of azaphilic addition, providing insight into pigment interconversion and composition drift during fermentation. Talarpurpurone A exhibited high color value and stable red coloration from pH 3 to 11, outperforming commercial preparations. These pigments displayed selected moderate activity against Gram-positive bacteria and lipid-lowering activity. Notably, bioinformatic and fermentation analysis supported the absence of citrinin biosynthesis in OUCMDZ-019. Overall, these results suggest that *Ash2* deletion is associated with azaphilone pigment diversification in *Talaromyces* under the tested conditions and provide a citrinin-free fungal strain for MPs production.

## GRAPHICAL ABSTRACT

**Figure:**
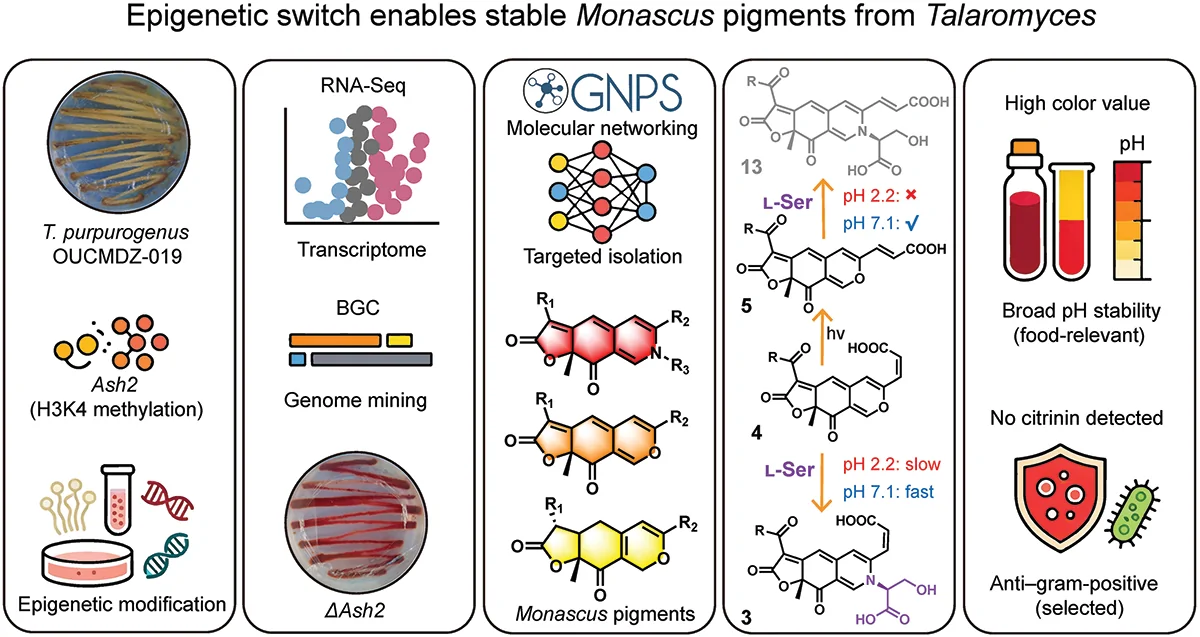

## 1. Introduction

Fungal secondary metabolites are a major source of natural-product chemical diversity and play important roles in ecological adaptation, interspecies interactions, and competition (Chen et al. 2019). Among them, polyketide pathways centered on polyketide synthases (PKSs) are particularly notable for their productivity, yielding a wide array of compounds (Pavesi et al. 2021). *Monascus* pigments (MPs) are representative azaphilone-type polyketide pigments (Gao et al. 2013; Chen et al. 2020) that typically occur as mixtures of closely related congeners and exhibit striking color phenotypes (Chen et al. 2017, 2019). In addition to their coloring properties, MPs/azaphilone compounds have also been reported to possess antibacterial, anti-inflammatory, and antioxidant activities (Hsu et al. 2011; Zhou et al. 2019; Zhang et al. 2020; Huang et al. 2023; Long et al. 2023; Husakova and Patakova 2025), suggesting that they may serve not only as “pigment products” but also as chemical mediators of defense and adaptation in specific fungal niches (Chen et al. 2019). Nevertheless, the profiles of MPs and the distribution of their congeners often vary markedly among fungal sources and cultivation conditions (Chen et al. 2019; He et al. 2023), and the biological basis of “congener compositional drift” remains to be elucidated at both the gene-cluster and regulatory levels (Chen et al. 2017, 2019).

From a biosynthetic perspective, azaphilone/MP formation is usually encoded by clustered biosynthetic gene clusters (BGCs) and diversified by the coordinated action of multiple tailoring enzymes (Chen et al. 2017; Pavesi et al. 2021). With the advance of fungal genomics, increasing evidence shows that many fungal genomes harbor rich repertoires of secondary-metabolite BGCs, yet substantial fractions are silent or weakly expressed under standard laboratory conditions, reflecting extensive “cryptic metabolic potential” (Lyu et al. 2020; Zhao et al. 2023). Therefore, how to effectively activate silent BGCs, translate genome-encoded metabolic capacity into detectable metabolite profiles, and further establish robust “gene cluster-metabolite” links remains a central question in natural-product discovery and fungal secondary-metabolism research (Lyu et al. 2020; Zhao et al. 2023).

Epigenetic regulation is considered an important upstream determinant of BGC expression states in fungi (Lyu et al. 2020; Zhao et al. 2023). Chromatin modifications can reshape genome-wide transcription by altering DNA accessibility and recruitment of the transcriptional machinery; in many filamentous fungi, genetic perturbation or chemical intervention targeting histone acetylation/deacetylation or methylation has been shown to derepress silent BGCs and thereby release new secondary metabolites (Lyu et al. 2020; Zhao et al. 2023). H3K4 methylation mediated by the COMPASS complex is generally associated with transcriptional activation and is thus thought to participate in BGC licensing and metabolome remodeling (Zhao et al. 2023; Wu et al. 2024). However, for pigment biosynthetic gene clusters (BGCs), especially azaphilone/MP pathways, systematic studies that provide empirical evidence for the associations between regulators, transcriptomes, and metabolite remain relatively limited. Accordingly, selecting a model fungus with strong azaphilone metabolic capacity and combining epigenetic target perturbation with multi-omics evidence chains should facilitate a clearer understanding of the intrinsic connection from chromatin regulation to BGC activation, metabolite release, and diversification (Lyu et al. 2020; Zhao et al. 2023; Wu et al. 2024).

In addition to enzyme-driven biosynthesis that defines the primary scaffold, the final congener composition of MPs may also be shaped by post-biosynthetic chemical processes (Chen et al. 2019). A classic example is azaphilic addition, in which orange MPs react with amines/amino acids to form nitrogen-containing red derivatives (Chen et al. 2017; He et al. 2023), potentially altering congener ratios and color phenotypes during cultivation (Chen et al. 2019; He et al. 2023). Environmental conditions (e.g. pH) may influence the extent of such transformations and thereby contribute to profile differences, but reported patterns across systems are not fully consistent (He et al. 2023). Therefore, evaluating condition associated transformation behaviors of representative congeners can provide experimental clues for interpreting links between cultivation conditions and congener composition (Chen et al. 2019; He et al. 2023).

In this study, we investigated the acidophilic fungus *Talaromyces purpurogenus* OUCMDZ-019, a prolific producer of azaphilone-type polyketides (Wang et al. 2011, 2013; Hou et al. 2025), aiming to elucidate its latent capacity for MPs production and the underlying regulatory basis. Using an epigenetic derepression strategy, we constructed an *Ash2* deletion mutant (Ash2 is a COMPASS subunit involved in H3K4 methylation) (Zhao et al. 2023; Wu et al. 2024) and assessed the impact of *ΔAsh2* on global transcription and secondary-metabolite profiles through transcriptomic and metabolomic analyses, which identified an MPs-related BGC showing marked upregulation in the mutant. To establish a traceable correspondence between transcriptional responses and specific compounds, we integrated genome mining, transcriptomics, and GNPS molecular networking to localize structurally related metabolite families, enabling targeted isolation and structural elucidation of a set of MP derivatives. In addition, we performed supplementary characterization of pH-linked azaphilic addition behavior of representative orange congeners and pigment properties to broaden our understanding of the sources of congener variation (He et al. 2023). Given that the risk of citrinin is an unavoidable concern in pigment-producing fungi, we also evaluated the citrinin biosynthetic potential of this strain via bioinformatic analyses (Ogihara et al. 2000; Huang et al. 2023). Overall, by integrating the research strategy of epigenetic perturbation, multi-omics association analysis, and molecular network guided isolation, this study provides an integrative framework for investigating the biosynthesis and structural diversification of azaphilone/MP pigments in *Talaromyces*, and lays a foundation for follow-up studies on pigment metabolism and fungal chemical ecology in a citrinin-free background.

## 2. Materials and methods

### 2.1. General experimental procedures

Optical rotations were recorded with a JASCO P-1020 digital polarimeter (JASCO, Tokyo, Japan). ECD spectra were determined using a JASCO J-815 spectropolarimeter. UV spectra were measured on a Thermofisher NanoDrop OneC microvolume UV-Vis spectrophotometer (Thermo Fisher Scientific, Waltham, MA, USA). IR spectra were performed on a Bruker Tensor-27 spectrophotometer (Bruker, Germany). NMR spectroscopic data were recorded on an Agilent DD2-500 (Agilent, America). HR-ESI-MS spectral data were collected on a MaXis quadrupole time-of-flight mass spectrometer (Waters, Milford, MA, USA). Low-resolution LC/ESI-MS data were measured using a Waters ACQUITY SQD 2 UPLC/MS system. Semipreparative HPLC was performed on a Waters or Hitachi HPLC equipped with a diode array detector (DAD detector). Other detailed procedures were described in the Supplemental material.

### 2.2. Fungal material

The fungus *Talaromyces purpurogenus* OUCMDZ-019 was isolated from red soil collected in Jianshui, Yunnan, China (Wang et al. 2011, 2013). The strain was deposited in the Key Laboratory of Marine Drugs, the Ministry of Education of China, Ocean University of China. The working strain was cultured on potato dextrose agar (PDA) slants and stored at 4 °C.

### 2.3. Bioinformatics analysis

The procedures for bioinformatics analysis and phylogenetic tree construction were described in experimental section of Supplemental material.

### 2.4. Construction of knockout mutant strains

Deletion of the epigenetic and global regulators code gene in the WT strain was performed through homologous recombination. The knockout plasmid was constructed by inserting the homologous arms flanking the target gene and the *neo*-resistance fragment into the linearized pMD19T vector using seamless cloning. The deletion cassette fragments were amplified from the knockout plasmid and then transferred into the WT, yielding the knockout mutant by PEG-mediated protoplast transformation. Transformants were selected and single-spore purified in the presence of geneticin. The genotype of the deletion mutant was confirmed by diagnostic PCR. The detailed procedures are described in the Supplemental material.

### 2.5. *Transcriptome sequencing of OUCMDZ-019 WT and the* ΔAsh2 *mutant*

The fresh mycelia of OUCMDZ-019WT and *ΔAsh*2 mutant cultivated in PDB medium (pH ~ 2.2) for 6 d were collected, flash-frozen in liquid nitrogen, and promptly submitted to Majorbio (Shanghai, China) for transcriptome sequencing. The detailed procedures were described in the Supplemental material.

### 2.6. Molecular network construction

The LC-MS/MS data for crude extract of *ΔAsh*2 cultivated in PDB medium (pH ~ 2.2) for 12 d were acquired. The raw files were converted to centroided open formats mzML using MSConvert, uploaded to the GNPS platform via WinSCP (https://gnps.ucsd.edu/), and molecular networks were constructed using the Classical Molecular Networking workflow. The detailed procedures are described in experimental section of Supplemental material.

### 2.7. Fermentation, extraction, and purification

The *ΔAsh*2 mutant was cultivated in PDB medium (pH ~ 2.2) under shaking condition at 180 r/min, 28 °C for 12 d. The fermentation broth was extracted repeatedly with an equal volume of ethyl acetate (EtOAc) five times, and the organic solvent was evaporated to dryness under vacuum to give a crude extract (20 g). The crude extract was purified by repeated separation by column chromatography using silica gel, LH-20, and HPLC with an ODS column, yielding compounds (**1−12**) for structural elucidation and bioactive evaluation. The details were described in Experimental section of Supplemental material.

### 2.8. Structural elucidation of compounds

The spectroscopic data of the new compounds are provided below, whereas those of the known compounds are available in the Supplemental material.

*Talarpurpurones A (**1**)*: red-black amorphous powder; ${\left[\alpha \right]}_{D}^{25}$− 655 (*c* 0.002, MeOH); UV (MeOH) *λ*_max_ (log *ε*) 200 (2.04), 272 (1.20), 425 (1.32), 515 (2.06) nm; ECD (0.94 mmol/L, MeOH) *λ*_max_ (*Δε*) 228 (−4.53), 273 (+17.04), 343 (−2.50), 409 (+0.79), 519 (−14.76) nm; IR (KBr) *v*_max_ 3424, 2927, 2855, 1717, 1632, 1461, 1319, 1200, 1150, 1095, 1020, 721 cm^−1^; ^1^H and ^13^C NMR, see Table S3; HRESIMS *m*/z 532.1979 [M + H]^+^ (calcd. for C_30_H_30_NO_8_, 532.1966).

*Talarpurpurones B (**2**)*: red-black amorphous powder; ${\left[\alpha \right]}_{D}^{25}$− 8680 (*c* 0.0005, MeOH); UV (MeOH) *λ*_max_ (log *ε*) 200 (2.05), 272 (1.23), 425 (1.33), 502 (1.66) nm; ECD (1.1 mmol/L, MeOH) *λ*_max_ (*Δε*) 229 (−0.97), 275 (+20.20), 332 (−2.85), 416 (+1.25), 511 (−12.0) nm; IR (KBr) *v*_max_ 3567, 2926, 2854, 1715, 1628, 1543, 1463, 1207, 1147, 726 cm^−1^; ^1^H and ^13^C NMR, see Table S4; HRESIMS *m/z* 456.2006 [M + H]^+^ (calcd. for C_25_H_30_NO_7_, 456.2017).

*Talarpurpurones C (**9**)*: yellow amorphous powder; ${\left[\alpha \right]}_{D}^{25}$− 162 (*c* 0.001, MeOH), UV (MeOH) *λ*_max_ (log *ε*) 228 (2.05), 387 (1.23) nm; ECD (1.3 mmol/L, MeOH) *λ*_max_ (*Δε*) 221 (−5.73), 258 (+2.49), 338 (−2.29), 401 (+6.06) nm; IR (KBr) *v*_max_ 3646, 3399, 2926, 2855, 1702, 1639, 1540, 1458, 1399, 1261, 1032, 805 cm^−1^; ^1^H and ^13^C NMR, see Table S11; HRESIMS *m/z* 391.2114 [M + H]^+^ (calcd. for C_22_H_31_O_6_, 391.2017).

*Talarpurpurones D (**10**)*: colorless oil; ${\left[\alpha \right]}_{D}^{25}$+ 122.9 (*c* 0.1, MeOH); UV (MeOH) *λ*_max_ (log *ε*) 214 (0.31), 247 (0.99), 321 (0.05) nm; ECD (1.2 mmol/L, MeOH) *λ*_max_ (*Δε*) 215 (+1.40), 246 (+7.95), 285 (−1.36), 322 (+0.65), 383 (−0.05) nm; IR (KBr) *v*_max_ 3646, 3359, 2926, 2855, 1778, 1716, 1699, 1650, 1540, 1380, 1078, 799 cm^−1^; ^1^H and ^13^C NMR, see Table S12; HRESIMS *m/z* 421.2218 [M + H]^+^ (calcd. for C_23_H_33_O_7_, 421.2221).

### 2.9. Electronic circular dichroism (ECD) calculations

The absolute configurations were determined by comparing experimental electronic circular dichroism (ECD) spectra with quantum-chemical ECD calculations. Conformational searches were conducted in Spartan 14 using the MMFF force field within an energy window of 10 kJ/mol, and the calculated ECD spectra of low-energy conformers were matched with the experimental data to assign absolute configurations. The detailed process was described in experimental section of Supplemental material.

### 2.10. *Azaphilic addition assay for orange* Monascus *pigments*

Orange MPs and _L_-serine were reacted under varying conditions, including different pH, reaction times, and reaction solvents, to verify azaphilic addition. Detailed procedures were described in Experimental section of Supplemental material.

### 2.11. Color value measurement and calculation method

The color values of the compounds were determined by UV-Vis spectroscopy at the maximum absorption wavelength (λ_max_) for each compound. The λ_max_ values of the compounds were measured between 386 and 517 nm (Table S18). The procedures for color value measurement and calculation method are described in the Supplemental material.

### 2.12. pH stability testing method

MPs were dissolved in methanol aqueous solution, adjusted to the appropriate absorbance, and mixed with buffers at different pH values to a final volume of 1 mL. All samples were incubated at 25 °C in the dark for 24 h. Color changes of MPs under different pH conditions were measured and recorded using a UV-Vis spectrophotometer. Detailed methods are provided in the Supplemental material.

### 2.13. Lipid-lowering activity and antimicrobial assay

The procedures for bioassays are described in the Supplemental material.

## 3. Results and discussion

### 3.1. *Genome mining and transcriptional regulator modulation of* T. purpurogenus *OUCMDZ-019*

Genome sequencing combined with antiSMASH analysis showed that the genome of *T. purpurogenus* OUCMDZ-019 contains about 80 secondary metabolite BGCs spanning diverse metabolite classes, indicating substantial secondary-metabolic potential. However, consistent with our previous isolation and characterization of metabolites from this strain (Wang et al. 2013; Hou et al. 2025), most putative products were not detected or identified under the previously used culture conditions, suggesting that a considerable portion of the corresponding BGCs may be silent or expressed at low levels. Guided by the concept that epigenetic/global regulation can activate silent gene clusters and expand fungal chemical space, we tried to employ transcriptional regulator perturbation by manipulating different epigenetic and global regulators (*HosA*, *HosB*, *LaeA*, *HdaA*, and *Ash2*) to reshape the secondary metabolic profile of this strain and to further explore its MPs-associated biosynthetic potential (Figures S2, S3; Table S1).

Among the regulator panel, we focused on *Ash*2, a COMPASS complex subunit involved in histone H3K4 methylation. The *Ash*2-coding gene was located and identified in the OUCMDZ-019 genome based on Local-BLAST and NCBI BLAST analyses, and its phylogenetic relationship with homologous proteins was examined (Figures 1a and S1). We then constructed an *Ash*2 deletion mutant (*ΔAsh*2) via homologous recombination using the geneticin-resistance gene *neo* as the selection marker, and diagnostic PCR confirmed complete deletion of *Ash*2 in positive transformants (Figure S2). *ΔAsh*2 exhibited the most pronounced pigment-related phenotypic shift and metabolic-profile alteration, and was therefore selected for subsequent integrated transcriptomic and metabolite-profiling analyses (Table S1 and Figure S4).

**Figure 1.**
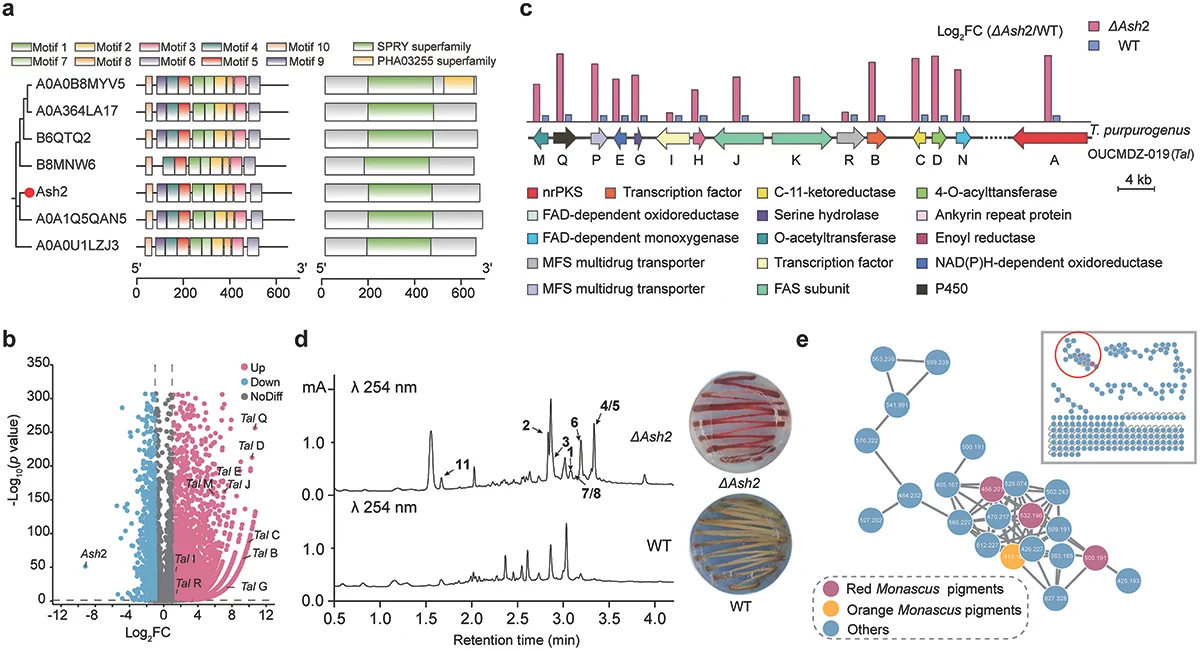
Identification of *Ash*2 and the impact of its deletion on the transcription of the MPs BGC and the metabolite profile in OUCMDZ-019. (a) Phylogenetic analysis of *Ash*2 with motif distribution and domain annotation. (b) Volcano plot of global transcriptomic changes in *ΔAsh*2 relative to WT (Log_2_FC > 1, *p* > 0.05). (c) Schematic organization of the MPs BGC and differential gene expression between WT and *ΔAsh*2. (d) HPLC chromatograms of EtOAc extracts monitored at λ_max_ 254 nm together with colony phenotypes of WT and *ΔAsh*2. (e) GNPS molecular networking analysis of the *ΔAsh*2 crude extract revealing the clustering pattern of MPs-related metabolites. Nodes highlighted in red represent the red *Monascus* pigments (red MPs) discussed in this study, orange nodes represent orange *Monascus* pigments (orange MPs), and blue nodes represent other metabolites.

### 3.2. *Analysis of transcriptome and secondary metabolite profile of the* ΔAsh2 *and WT strains*

To assess how *Ash2* deletion was associated with changes in secondary-metabolite gene expression and pigment-associated metabolic output, we performed RNA-seq on the *ΔAsh2* mutant and the wild-type (WT) strain and visualized differentially expressed genes (DEGs) using a volcano plot (Figure 1b). Overall, the number of upregulated genes in *ΔAsh2* was markedly higher than that of downregulated genes, indicating broad transcriptional activation and reorganization of expression programs upon *Ash2* deletion (Figure 1b). Meanwhile, *Ash2* transcripts in *ΔAsh2* decreased to near-background levels, further confirming effective deletion at the transcriptional level (Figure 1b). This genome-wide activation was consistent with the pronounced pigment-related phenotypic shift of *ΔAsh2*, suggesting systemic reprogramming of secondary metabolism.

We then focused on the MPs-related BGC. Most genes within this cluster were significantly upregulated in *ΔAsh2*; the cluster organization and expression changes are summarized in Figure 1c (Table S2). To test whether transcriptional activation translated into altered metabolite output, fermentation extracts from *ΔAsh2* and WT cultivated under identical conditions were analyzed by UPLC-MS (Figure 1d). Compared with WT, *ΔAsh2* displayed a strongly remodeled metabolite fingerprint, featuring a group of products with UV characteristics typical of MPs and molecular weights mainly in the 390–550 Da range. In contrast, the hydrogenated azaphilone derivatives previously reported as dominant metabolites in WT were substantially reduced in *ΔAsh2* (Figure 1d). Taken together with the pigment phenotype and coordinated upregulation of the MPs-related BGC, these metabolomic differences indicate that MP-related compounds represent the major class of differential metabolites in *ΔAsh2* relative to WT. Because *ΔAsh2* also exhibited broad transcriptomic changes, the enhanced expression of the MPs-related cluster may reflect both pathway-associated regulation and broader pleiotropic effects of *Ash2* deletion; direct locus-specific chromatin validation will be required to distinguish these possibilities.

### 3.3. GNPS molecular networking analysis and compounds characterization

To systematically delineate metabolite families associated with the MPs pathway under the *ΔAsh2*-induced metabolomic reprogramming and to improve the efficiency of discovering new congeners, we constructed a GNPS molecular network based on MS/MS data from the crude extract of the *ΔAsh2* mutant (Figures 1e and S5). The network revealed well-defined clusters corresponding to metabolites related to MP, which were clearly separated from other metabolite classes. Moreover, adjacent nodes within the same cluster showed a continuous distribution of MS/MS spectral similarity, suggesting that these metabolites share a common core scaffold and gain structural diversity through stepwise modifications (Figure 1e). This “family-like” organization is consistent with the coordinated transcriptional activation of the MPs BGC described above, providing metabolomics-level support for grouping differential features into a coherent biosynthetic product spectrum.

Within the largest MPs cluster, the node at *m/z* 413.16 could be assigned to the orange MP PP-O (**4**) and its analogue talaralbol B (**5**). A series of nodes connected to this feature displayed signatures consistent with amine-adduct derivatives generated via azaphilic addition, implying that orange MPs may serve as a central hub for diversification within this cluster. In addition, the node at *m/z* 383.185 was tentatively proposed as monascorubrin, whose terminal carboxylation of the branched carbon chain could yield PP-O, further suggesting multi-step derivatization and congener interconversion centered on orange MPs. Guided by the GNPS clustering information together with UPLC-MS differential metabolite fingerprints, we performed network-informed targeted isolation from the *ΔAsh2* fermentation extract and obtained 12 MPs and related derivatives (**1**–**12**) (Figure 2a). Notably, the interconnections suggested by the network also provide clues for interpreting dynamic changes in pigment composition during fermentation.

**Figure 2.**
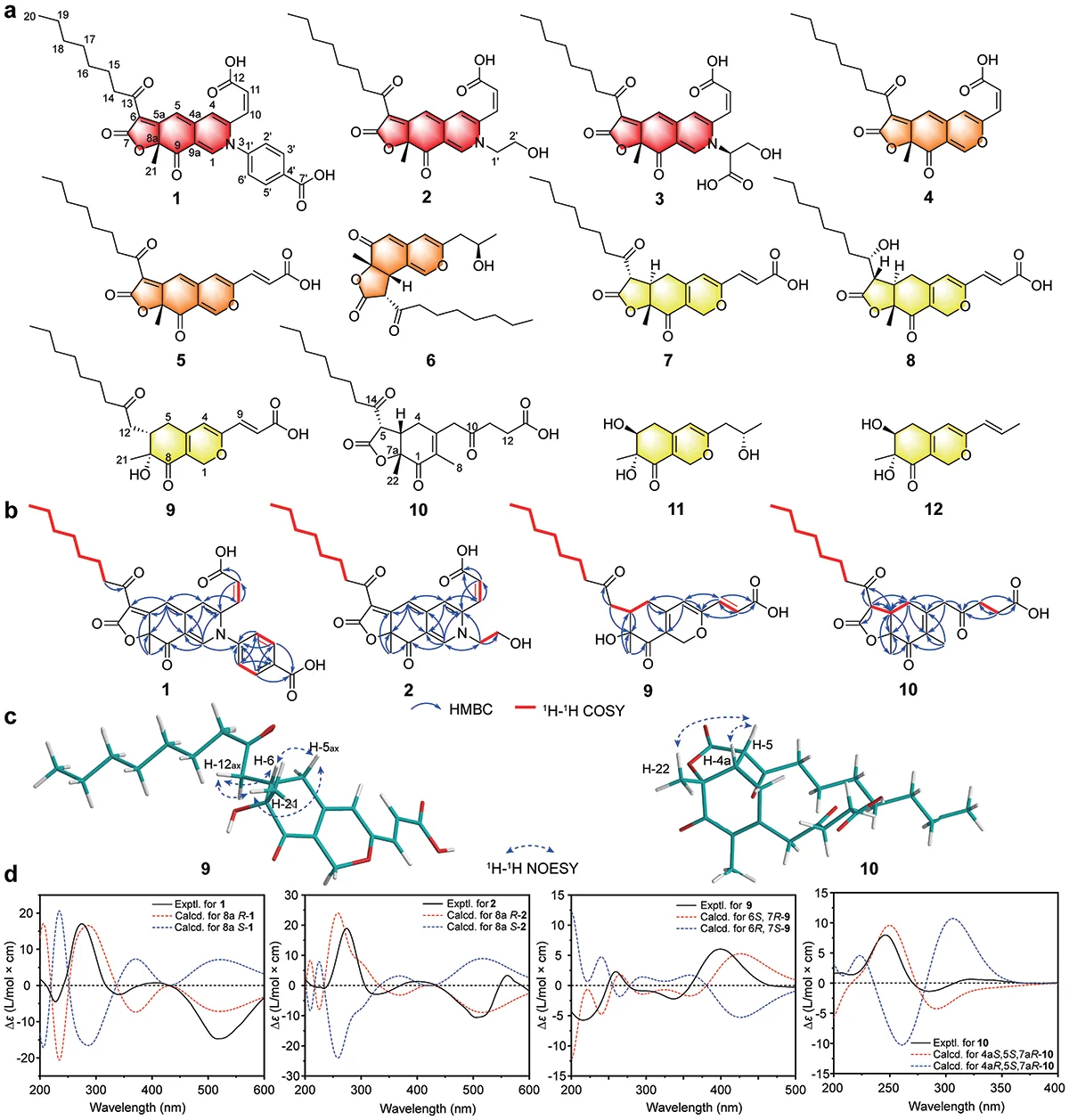
Structural characterization of MPs derivatives isolated in this study and stereochemical assignment of the new compounds. (a) Chemical structures of compounds **1**–**12**, including typical MPs (red: **1**–**3**; orange: **4**, **5**; yellow: **7**, **8**) and atypical MPs (orange: **6**; yellow: **9**, **11**, **12**; white: **10**). (b) Key ^1^H-^1^H COSY and HMBC correlations for the new compounds **1**, **2**, **9**, and **10**. (c) Diagnostic ^1^H−^1^H NOESY correlations for compounds **9** and **10** used to establish the relative configurations. (d) Comparison of experimental and calculated ECD spectra for compounds **1**, **2**, **9**, and **10** to determine the absolute configurations.

Based on the network localization and targeted isolation described above, the structures and absolute configurations of compounds **1**–**12** are presented below, with emphasis on the key structural features of the new compounds and their relationships to known MPs.

Talarpurpurones A (**1**) was obtained as a red-black amorphous powder; its molecular formula was established as C_30_H_30_NO_8_ based on HRESIMS (*m*/*z* 532.1979 [M + H]^+^), consistent with 17 degrees of unsaturation. Comprehensive NMR (Figures 2b and S7−S14; Table S3) analysis suggested that compound **1** possessed a bulgarialactone-type azaphilone core skeleton and was highly similar to the known *Monascus* pigment PP-V (Ogihara et al. 2000). The main differences between **1** and PP-V were that one 4-aminobenzoic acid group was attached to the azaphilone skeleton of **1** via azaphilic addition reaction, which was supported by the observation of two pairs of mutually coupled aromatic protons H-2′/6′ (*δ*_H_ 7.61, d, *J* = 8.3 Hz) and H-3′/5′ (*δ*_H_ 8.06, d, *J* = 8.3 Hz) combined with the key HMBC correlations from H-3′/H-5′ to C-1′, H-3′/H-5′ to C-7′, and H-1 to C-1′. The coupling constant between H-10 and H-11 (*J* = 11.9 Hz) defined the double bond to be also in the *Z* orientation.

The absolute configuration at C-8a in compound **1** is closely related to its specific optical rotation (Steyn and Vleggaar 1976; Kanokmedhakul et al. 2006). Compound **1** exhibited a negative specific rotation, which agrees in sign with that of the structurally related rubropunctatin (Steyn and Vleggaar 1976) but contrasts with that of (+)-rotiorin (Jackman et al. 1958). This comparison suggests an *R*-configuration at the C-8a for compound **1**. To further confirm the absolute configuration at C-8a, the theoretical ECD spectrum was obtained by the TDDFT [B3LYP/6-31G(d)] method. The measured ECD spectrum of compound **1** matched well with the calculated ECD curve of 8a*R*-**1** (Figure 2d), further determining the stereochemical structure of **1**. Finally, the structure of compound **1** was characterized and given the name as talarpurpurones A.

Talarpurpurones B (**2**) was obtained as a red-black amorphous powder with a molecular formula of C_25_H_30_NO_7_ based on HRESIMS (*m*/*z* 456.2006 [M + H]^+^), indicating 12 degrees of unsaturation. The ^1^H and ^13^C NMR data (Figures 2b and S15−S22, Table S4) and UV absorbance profiles revealed a remarkable resemblance to those of **1**, differing only by the substituent attached to the C-2 position of the azaphilone skeleton: a 4-aminobenzoic acid group in **1** versus a 2-aminoethanol group in **2**. The presence of a 2-aminoethanol group was inferred by the methylene protons at *δ*_H_ 4.09 (m)/3.65 (dd, *J* = 6.8, 4.4 Hz) combined with the key HMBC connections of H-1′ to C-1/C-3/C-2′ and H-1 to C-1′. The coupling constant between H-10 and H-11 (*J* = 12.1, 11.9 Hz) indicated the presence of a *Z*-configuration. Based on the closely matched experimental ECD curves of compound **2** (Figure 2d) as well as the negative optical rotation of **2**, the absolute configuration of C-8a was presumed as *R*. ECD calculations further validated its 8a*R*-configuration (Figure 2d). Finally, the structure of compound **2** was elucidated and named as talarpurpurones B (**2**).

Talarpurpurones C (**9**) was isolated as a yellow amorphous powder. The molecular formula was determined to be C_22_H_31_O_6_ by the HRESIMS peak at *m*/*z* 391.2114 [M + H]^+^, indicating 8 degrees of unsaturation. The combined evidence from ^1^H and ^13^C NMR analysis indicated that compound **9** closely resembles those of the planar structure of known azaphilone monaphilone A (Hsu et al. 2010) (Figures 2b and S54−S62, Table S11)，except for the presence of a carboxyl group at the C-11 position in **9**. The coupling constant between H-9 and H-10 (*J* = 15.5 Hz) suggested the presence of an *E*-configuration. The relative configuration of compound **9** was determined by NOESY correlations. In the NOESY spectrum of **9** (Figure 2c), the correlation between H-12_ax_ and H_3_-21, H-12_ax_ and H-6, H-5_ax_ and H_3_-21, as well as H-5_ax_ and H-6 were observed, which indicated the syn relationship between H-6 and H_3_-21. To further confirm the absolute configuration of **9**, the theoretical ECD spectrum was obtained by the TDDFT [B3LYP/6-31G(d)] method. The measured ECD spectrum of compound **9** matched well with the calculated ECD curve of 6*S*, 7*R*-**9** (Figure 2d), determining the stereochemical structure of **9**. In addition, monaphilone A has been reported to possess an *R* configuration at C-6. The opposite sign of the optical rotation observed for compound **9** relative to monaphilone A further supports assignment of an *S* configuration at C-6 for **9**. Therefore, the absolute configurations of **9** was assigned as 6*S*, 7*R*. Finally, the structure of compound **9** was established and named as talarpurpurones C.

Talarpurpurones D (**10**) was isolated as a colorless oil. The molecular formula was deduced to be C_23_H_33_O_7_ by the *m*/*z* 421.2218 [M + H]^+^ of the HRESIMS, suggesting 7 degrees of unsaturation. Comprehensive analysis of NMR (Figures 2b and S63−S71, Table S12) revealed that compound **10** possessed a cyclohex-2-enone fused with *γ*-lactone bicyclic scaffold and was highly similar to purpureusone (Hsu et al. 2010)，except for the presence of a carboxyl group at the C-13 position in **10**. The relative configuration of compound **10** was determined by NOESY correlations. In the NOESY spectrum of **10** (Figure 2c), the correlation between H-5 and H-4a as well as H-5 and H_3_-22 was observed, which indicated the syn relationship between H-4a, H-5, and H_3_-22. To determine the absolute configuration of **10**, the theoretical ECD spectrum was obtained by the TDDFT [B3LYP/6-31G(d)] method. The measured ECD spectrum of compound **10** matched well with the calculated ECD curve of 4a*S*, 5*S*, 7a*R*-**10** (Figure 2d), determining the stereochemical structure of **10**. Moreover, the specific rotation of compound **10** is consistent with that reported for purpureusone (Hsu et al. 2010), further corroborating the absolute configuration of **10**.  Therefore, the absolute configurations of **10** was established as 4a*S*, 5*S*, 7a*R* and named as talarpurpurones D.

The structures of known compounds were identified as atrorosin S (**3**) (Isbrandt et al. 2020) (Figures 2a and S23−S29, Table S5), PP-O (**4**) (Ogihara and Oishi 2002) (Figures 2a and S30−S34, Table S6), talaralbol B (**5**) (Bai et al. 2021) (Figures 2a and S35−S36, Table S7), monasfluol B (**6**) (Promsuk et al. 2023) (Figures 2a and S37−S41, Table S8), talaralbol A (**7**) (Bai et al. 2021) (Figures 2a and S42−S47, Table S9), sequoiamonascin C (**8**) (Gong et al. 2023) (Figures 2a and S48−S53, Table S10), monascusone A (**11**) (Jongrungruangchok et al. 2004) (Figure 2a and S72−S76, Table S13), and FK17-P2b1 (**12**) (Promsuk et al. 2023) (Figures 2a and S77−S81, Table S14) by careful comparison of their spectroscopic and chiroptical data with those reported in the literature.

Many of these congeners exhibited terminal carboxylation on the branched side chain, highlighting a coherent congener series passociated with the MPs-related metabolite family observed in *ΔAsh2*. Because ionization of a terminal carboxyl group is sensitive to environmental pH, this shared feature suggests that pH may influence both the physicochemical state and the propensity for post-biosynthetic transformations within this congener family. We therefore examined pH-associated interconversion behaviors using representative orange MPs as model substrates, with a particular focus on azaphilic addition reactions with amino acids.

### 3.4. Analysis of azaphilic addition reaction conditions for orange MPs

Azaphilic addition between the representative orange MPs (**4**, **5**) and _L_-Ser was examined under a range of conditions, including pH, reaction time, and solvent system, to probe pH-associated post-biosynthetic interconversion. Under neutral conditions (pH 7.2), compound **4** was converted to the corresponding amine-adduct **3** more rapidly than under acidic conditions (pH 2.2), indicating that near-neutral pH favors the reaction (Figure 3a). In contrast, at pH 2.2 the reaction proceeded more slowly, but a higher overall conversion could be achieved upon extending the reaction time (Figure 3a). The conversion of **4** and _L_-Ser to **3**, observed in both H_2_O and PDB medium with greater efficacy at pH 7.2, confirmed that the PDB medium did not significantly influence the rate of azaphilic addition reaction, while reiterating its preference for neutral conditions (Figure 3b). The observation that compound **5** reacted with _L_-Ser to yield **13** under neutral, but not acidic conditions demonstrated that this *E*-configured orange MP can undergo azaphilic addition to form red MPs only at neutral pH (Figure 3c). This result provided a rationale for the exclusive isolation of *Z*-configured red MPs from the *ΔAsh2* strain. Combined with the observation that *E*-configured orange MPs were more abundant than their *Z*-configured counterparts during isolation, we speculate that the orange MPs are initially produced with an *E*-configured side-chain via biosynthetic enzyme catalysis. These *E*-configured orange MPs then undergo a non-enzymatic azaphilic addition reaction to yield the red MPs. Concurrently, a portion of the *E*-configured orange MPs can undergo photoisomerization (or other influences) to convert to the *Z*-configuration (Bai et al. 2021).

**Figure 3.**
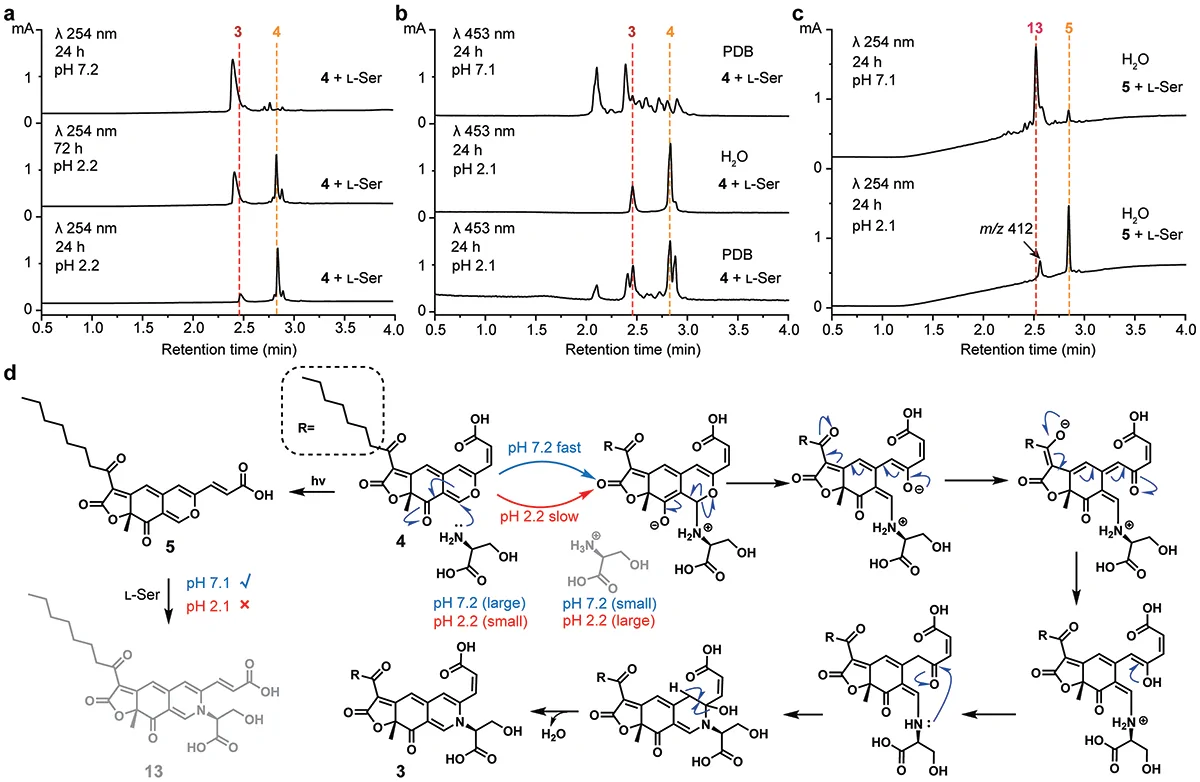
Azaphilic addition of orange MPs with _L_-Ser and a proposed scheme for pH-dependent chemical transformation. (a) UPLC chromatograms of orange MP **4** incubated with _L_-Ser in water under different pH values and reaction times, monitored at 254 nm. (b) UPLC chromatograms of orange MP **4** incubated with _L_-Ser in PDB medium or water at different pH values, monitored at 453 nm. (c) UPLC chromatograms of orange MP **5** incubated with _L_-Ser under different pH conditions, monitored at 254 nm. (d) Proposed scheme for pH-dependent chemical transformation, illustrating that orange MP **4** can interconvert with orange MP **5** upon light irradiation, that **4** undergoes azaphilic addition with _L_-Ser followed by rearrangement and dehydration to yield the red MP **3**, and that orange MP **5** undergoes azaphilic addition with _L_-Ser to yield **13** at pH 7.1.

Based on the above experimental results, we provide the following chemical rationale for the pH-dependent azaphilic addition observed under our assay conditions (Figure 3d). The orange MP **4** contains a pronounced electrophilic center enabled by its conjugated carbonyl enone system. After addition of _L_-Ser, the amino group first attacks the electrophilic site of **4** in a nucleophilic manner to form an N−C bond adduct intermediate; the intermediate then undergoes proton transfer and resonance reorganization, which induces opening and reclosure of the original oxygen-containing scaffold accompanied by dehydration, ultimately expanding the conjugated system and incorporating nitrogen into the chromophore, to afford the *N*-containing red pigment **3**. Meanwhile, **4** can undergo photoinduced isomerization or tautomerization under irradiation to yield **5** (Bai et al. 2021), and **5** can be converted to **13** via azaphilic nucleophilic addition at pH 7.1. This chemical rationale is consistent with the experimental observations of the azaphilic reaction: First, the conversion of **4** to **3** proceeds more rapidly at pH 7.2 because under neutral conditions, the amino group of _L_-Ser remains largely in the unprotonated form with higher nucleophilicity, thereby promoting the initial addition step. Second, at pH 2.2 adjusted with HCl, the amino group predominantly exists as −NH^3+^, so the addition kinetics are suppressed and the reaction proceeds more slowly. However, prolonged incubation can still lead to more complete conversion due to the gradual accumulation of the adduct and acid-favored downstream rearrangement and dehydration that stabilize the product. Third, compound **5** yields **13** only under neutral conditions but not under acidic conditions, indicating that entry from **5** into the azaphilic addition pathway is more sensitive to amino nucleophilicity and the required reactive conformation, and the loss of nucleophilicity under acidic conditions disfavors effective addition, making formation of **13** difficult to observe. These results suggest that pH and residence time during fermentation and in acidic food matrices can markedly influence azaphilic addition and congener interconversion, thereby reshaping the final pigment profile and color stability.

### 3.5. Assays of the physicochemical properties and bioactivities for MPs

Color value can serve as an integrated metric for evaluating color intensity and optical output of pigments, and to some extent reflects the relative contribution of different congeners to overall coloration. As shown in Figure 4a (Table S18), the isolated pigments exhibited an overall trend of color values in the order red MPs > yellow MPs > orange MPs, and these values were markedly higher than those previously reported for MP mixtures, suggesting that the MPs-related pigment family observed in *ΔAsh2* has strong chromogenic capacity. Notably, red MP **1** displayed the highest color value (27,520 U/g), indicating the strongest tinting strength among the congeners tested.

**Figure 4.**
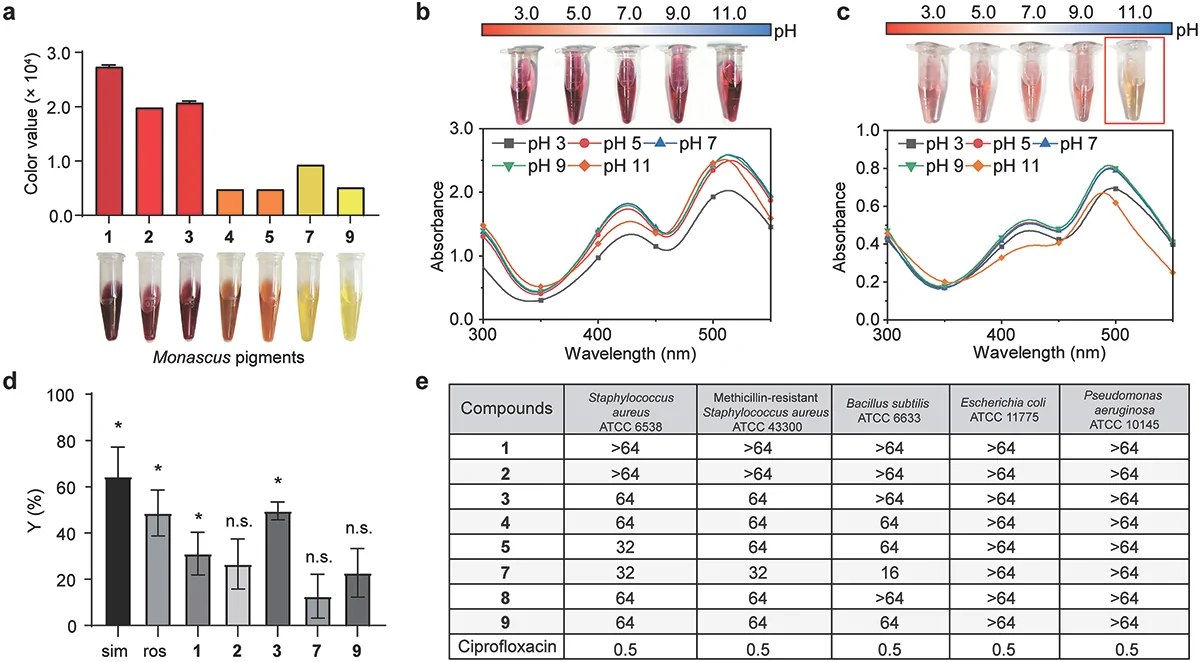
Color value, pH stability, and bioactivity evaluation of MPs derivatives. (a) Color values and representative photographs of solutions of compounds **1**–**5**, **7**, and **9**. (b, c) Color changes and UV-Vis absorption spectra of compound **1** (b) and a commercial MP (c) preparation across pH 3–11 to assess pH stability. (d) Lipid-lowering activity of compounds **1**–**3**, **7**, and **9**, with simvastatin (sim) and rosiglitazone (ros) as positive controls; the *y*-axis indicates inhibition rate (%). The y-axis represents the inhibition rate relative to the oleic acid + palmitic acid (OA + PA) blank model group, with the inhibition rate of the blank group defined as 0%. A two-sided unpaired two-sample *t*-test was used to analyze the differences between each treatment group and the aforementioned blank model group. Error bars represent the mean ± SD. n.s. (not significant) *p* > 0.05; *p < 0.05. (e) Minimum inhibitory concentration (MIC, μg/mL) values of compounds **1**–**5** and **7**–**9** against the indicated bacterial strains, with ciprofloxacin as the positive control.

Because pH is an important variable in fungal cultivation environments and many ecological microhabitats, and may shape product profiles by altering pigment ionization states, congener stability, and post-biosynthetic transformations, we further evaluated the color phenotype and spectroscopic responses of red MP **1** across different pH conditions. Compound **1** maintained a consistently bright red appearance over pH 3.0–11.0, with no obvious changes in hue or intensity (Figures 4b and S82), and its UV-Vis spectra showed no pronounced hypochromic effect. In contrast, a commercial red MP preparation appeared weaker (pinkish) at pH 3.0–9.0 and shifted from pink to pale orange at pH 11.0, accompanied by a clear hypochromic effect in the UV-Vis spectra (Figures 4c and S82), indicating reduced stability under strongly alkaline conditions. This difference is consistent with previous observations that red MPs bearing an amino-acid moiety generally exhibit improved pH stability compared with conventional red MPs (He et al. 2023). In addition, the isolated MPs remained stable under strongly acidic conditions (pH 2.2), in line with pigment output under the acidophilic cultivation background of this strain. Together with structural features revealed by elucidation (terminal carboxylation on a branched side chain for many congeners), our data provide experimental support for the view that terminal carboxylation may be associated with enhanced stability of azaphilone pigments under acidic conditions (Xue et al. 2023).

Beyond pigmentation phenotypes, we performed preliminary *in vitro* functional assays for pigments **1**–**3**, **7**, and **9** to obtain clues about potential ecological roles of this pigment class. Overall, lipid-lowering effects were modest *in vitro*; compounds **1** and **3** showed limited but statistically significant inhibition relative to the negative control (oleic acid/palmitic acid), with inhibition rates of 31.08% and 49.55%, respectively (Figure 4d). By contrast, antibacterial assays revealed clearer trends: the tested MPs were notably more active against Gram-positive bacteria (*Staphylococcus aureus*, MRSA, and *Bacillus subtilis*) than against Gram-negative bacteria (*Escherichia coli* and *Pseudomonas aeruginosa*). In particular, yellow MP **7** exhibited moderate antibacterial activity, with MIC values of 32, 32, and 16 μg/mL against *S. aureus*, MRSA, and *B. subtilis*, respectively (Figure 4e). These results suggest that, in addition to coloration, these pigments may possess bioactive attributes that warrant further evaluation under conditions more closely resembling natural or cultivation-relevant settings.

Notably, based on bioinformatic analysis, *T. purpurogenus* OUCMDZ-019 showed no indication of citrinin biosynthesis (Figure S6). This provides a cleaner genetic background for future studies of MP-related BGC regulation and pigment chemical ecology in *Talaromyces*, and supports further screening of lower-risk pigment-producing strains.

In summary, using the acidophilic filamentous fungus *Talaromyces purpurogenus* OUCMDZ-019 as a model, we found that deletion of Ash2 was associated with broad transcriptomic and metabolomic remodeling, including enhanced expression of an MPs-related biosynthetic gene cluster and increased production of a corresponding azaphilone pigment family. By integrating genome mining, transcriptomics, and GNPS molecular networking, we established an association between Ash2 perturbation, candidate pigment-BGC expression, and the output of a corresponding metabolite family; however, direct locus-specific chromatin validation will be required to define the underlying chromatin-level mechanism. This framework enabled network-informed targeted isolation, yielding four new azaphilone pigments (talarpurpurones A–D) together with eight known analogues. Condition-dependent transformation assays with representative orange congeners showed a clear pH dependence of azaphilic addition, providing experimental clues to interpret congener drift and interconversion during cultivation. In parallel, talarpurpurone A exhibited a high color value and maintained a stable red phenotype across a broad pH range, highlighting phenotypic robustness of this pigment class under variable environmental conditions. Several compounds displayed moderate inhibitory activity against Gram-positive bacteria, whereas lipid-lowering effects were generally weak *in vitro*, offering preliminary insights into potential ecological functions beyond pigmentation. Notably, bioinformatic analysis and fermentation analysis confirmed the absence of citrinin biosynthetic genes in strain OUCMDZ-019, which provides a favorable condition for investigating the regulatory mechanisms of azaphilone pigments and fungal chemical ecology in a cleaner genetic background. Overall, this study proposes a research framework centered on chromatin regulators and assisted by multi-omics and molecular network technologies, which advances understanding of the biosynthesis and structural diversification of azaphilone pigments in *Talaromyces*.

## Supplementary Material

20260403-Supplemental material_re.docx

## References

[cit0001] Bai W, Jing L, Guan Q, Tan R. 2021. Two new azaphilone pigments from *Talaromyces albobiverticillius* and their anti-inflammatory activity. J Asian Nat Prod Res. 23(4):325–332. doi: 10.1080/10286020.2020.1847093. 33200618

[cit0002] Chen C, Tao H, Chen W, Yang B, Zhou X, Luo X, Liu Y. 2020. Recent advances in the chemistry and biology of azaphilones. RSC Adv. 10(17):10197–10220. doi: 10.1039/d0ra00894j. PMC9050426 35498578

[cit0003] Chen W, Chen R, Liu Q, He Y, He K, Ding X, Kang L, Guo X, Xie N, Zhou Y, et al. 2017. Orange, red, yellow: biosynthesis of azaphilone pigments in *Monascus* fungi. Chem Sci. 8(7):4917–4925. doi: 10.1039/c7sc00475c. PMC5603960 28959415

[cit0004] Chen W, Feng Y, Molnár I, Chen F. 2019. Nature and nurture: confluence of pathway determinism with metabolic and chemical serendipity diversifies *Monascus* azaphilone pigments. Nat Prod Rep. 36(4):561–572. doi: 10.1039/c8np00060c. PMC6470053 30484470

[cit0005] Gao J, Yang S, Qin J. 2013. Azaphilones: chemistry and biology. Chem Rev. 113(7):4755–4811. doi: 10.1021/cr300402y. 23581812

[cit0006] Gong Y, Li X, Shi L, Feng L, Wang J, Tan N, Wang Z. 2023. Azaphilones with anti-colon cancer activities from the plant endophytic fungus *Talaromyces primulinus* WZ-883. Phytochem Lett. 57:115–125. doi: 10.1016/j.phytol.2023.08.006.

[cit0007] He L, Liu C, Chen S, Zhang J, Gao M, Li L. 2023. Precursor-directed production of water-soluble red *Monascus* pigments with high thermal stability via azaphilic addition reaction-based semi-synthesis. Food Chem X. 20:100940. doi: 10.1016/j.fochx.2023.100940. PMC10739767 38144809

[cit0008] Hou C, Wang Y, Meng X, Lu L, Fu P, Zhu W, Wang Y. 2025. Bioactive hydrogenated azaphilones from acid-tolerant fungus *Penicillium purpureum* OUCMDZ-019. J Antibiot. 78(5):281–287. doi: 10.1038/s41429-025-00818-7. 40128317

[cit0009] Hsu Y, Hsu L, Liang Y, Kuo Y, Pan T. 2010. Monaphilones A−C, three new antiproliferative azaphilone derivatives from *Monascus purpureus* NTU 568. J Agric Food Chem. 58(14):8211–8216. doi: 10.1021/jf100930j. 20597545

[cit0010] Hsu Y, Hsu L, Liang Y, Kuo Y, Pan T. 2011. New bioactive orange pigments with yellow fluorescence from *Monascus*-fermented dioscorea. J Agric Food Chem. 59(9):4512–4518. doi: 10.1021/jf1045987. 21506577

[cit0011] Huang Y, Fu R, Yin W, Chen R, Zhao C, Hu F, Bao G, Lu R. 2023. Metabolites analysis and new bioactive pigments from a *Monascus purpureus* strain with low citrinin. Microchem J. 195:109441. doi: 10.1016/j.microc.2023.109441.

[cit0012] Husakova M, Patakova P. 2025. Purified *Monascus* pigments: biological activities and mechanisms of action. J Nat Prod. 88(2):607–615. doi: 10.1021/acs.jnatprod.4c01008. PMC11877510 39906945

[cit0013] Isbrandt T, Tolborg G, Ødum A, Workman M, Larsen TO. 2020. Atrorosins: a new subgroup of *Monascus* pigments from *Talaromyces atroroseus*. Appl Microbiol Biotechnol. 104(2):615–622. doi: 10.1007/s00253-019-10216-3. 31802169

[cit0014] Jackman GB, Robertson A, Travers RB, Whalley WB. 1958. The chemistry of fungi. Part XXXIV. Rotiorin, a metabolite of *Penicillium sclerotiorum* van Beyma. J Chem Soc. 161:1825–1832. doi: 10.1039/jr9580001825.

[cit0015] Jongrungruangchok S, Kittakoop P, Yongsmith B, Bavovada R, Tanasupawat S, Lartpornmatulee N, Thebtaranonth Y. 2004. Azaphilone pigments from a yellow mutant of the fungus *Monascus kaoliang*. Phytochemistry. 65(18):2569–2575. doi: 10.1016/j.phytochem.2004.08.032. 15451319

[cit0016] Kanokmedhakul S, Kanokmedhakul K, Nasomjai P, Louangsysouphanh S, Soytong K, Isobe M, Kongsaeree P, Prabpai S, Suksamrarn A. 2006. Antifungal azaphilones from the fungus *Chaetomium cupreum* CC3003. J Nat Prod. 69(6):891–895. doi: 10.1021/np060051v. 16792406

[cit0017] Long P, Zhu L, Lai H, Xu S, Dong X, Shao Y, Wang L, Cheng S, Liu G, He J, et al. 2023. *Monascus* red pigment liposomes: microstructural characteristics, stability, and anticancer activity. Foods. 12(3):447. doi: 10.3390/foods12030447. PMC9914458 36765975

[cit0018] Lyu H, Liu H, Keller NP, Yin W. 2020. Harnessing diverse transcriptional regulators for natural product discovery in fungi. Nat Prod Rep. 37(1):6–16. doi: 10.1039/c8np00027a. 31033969

[cit0019] Ogihara J, Kato J, Oishi K, Fujimoto Y, Eguchi T. 2000. Production and structural analysis of PP-V, a homologue of monascorubramine, produced by a new isolate of *Penicillium* sp. J Biosci Bioeng. 90(5):549–554. doi: 10.1016/s1389-1723(01)80039-6. 16232908

[cit0020] Ogihara J, Oishi K. 2002. Effect of ammonium nitrate on the production of PP-V and monascorubrin homologues by *Penicillium* sp. AZ. J Biosci Bioeng. 93(1):54–59. doi: 10.1016/s1389-1723(02)80054-8. 16233165

[cit0021] Pavesi C, Flon V, Mann S, Leleu S, Prado S, Franck X. 2021. Biosynthesis of azaphilones: a review. Nat Prod Rep. 38(6):1058–1071. doi: 10.1039/d0np00080a. 33527918

[cit0022] Promsuk G, Chuawong P, Songjanthuek P, Thaisri S, Yongsmith B, Wattana-Amorn P. 2023. Absolute configuration of azaphilones from *Monascus kaoliang* KB9 and solvent effects on their keto and enol forms. Nat Prod Res. 37(13):2181–2188. doi: 10.1080/14786419.2022.2034812. 35142570

[cit0023] Steyn PS, Vleggaar R. 1976. The structure of dihydrodeoxy-8-*epi*-austdiol and the absolute configuration of the azaphilones. J Chem Soc Perkin Trans. (2):204–206. doi: 10.1039/p19760000204. 942960

[cit0024] Wang H, Wang Y, Liu P, Wang W, Fan Y, Zhu W. 2013. Purpurides B and C, two new sesquiterpene esters from the aciduric fungus *Penicillium purpurogenum* JS03-21. Chem Biodiver. 10(7):1185–1192. doi: 10.1002/cbdv.201200175. 23847064

[cit0025] Wang H, Wang Y, Wang W, Fu P, Liu P, Zhu W. 2011. Anti-influenza virus polyketides from the acid-tolerant fungus *Penicillium purpurogenum* JS03-21. J Nat Prod. 74(9):2014–2018. doi: 10.1021/np2004769. 21879714

[cit0026] Wu Z, Gao H, Liu Z. 2024. COMPASS core subunits MpSet1 and MpSwd3 regulate *Monascus* pigments synthesis in *Monascus purpureus*. J Basic Microbiol. 64(4):2300686. doi: 10.1002/jobm.202300686. 38362934

[cit0027] Xue Y, Wang L, Zhang X, Wang Z. 2023. Terminal carboxylation of branched carbon chain contributing to acidic stability of azaphilone pigments from a new isolate of *Talaromyces amestolkiae*. Food Chem. 424:136338. doi: 10.1016/j.foodchem.2023.136338. 37207602

[cit0028] Zhang X, Liu C, Tian W, Zhang H, Li P, Wang J, He W. 2020. Theoretical and experimental investigation of the antioxidative activity of monascin. Food Funct. 11(7):5915–5923. doi: 10.1039/c9fo02410g. 32584351

[cit0029] Zhao F, Sun C, Liu Z, Cabrera A, Escobar M, Huang S, Yuan Q, Nie Q, Luo KL, Lin A, et al. 2023. Multiplex base-editing enables combinatorial epigenetic regulation for genome mining of fungal natural products. J Am Chem Soc. 145(1):413–421. doi: 10.1021/jacs.2c10211. PMC10162584 36542862

[cit0030] Zhou W, Guo R, Guo W, Hong J, Li L, Ni L, Sun J, Liu B, Rao P, Lv X. 2019. *Monascus* yellow, red and orange pigments from red yeast rice ameliorate lipid metabolic disorders and gut microbiota dysbiosis in Wistar rats fed on a high-fat diet. Food Funct. 10(2):1073–1084. doi: 10.1039/c8fo02192a. 30720827

