# Supplementary material for: Chromatin-mediated upregulation of Monascus pigments in Talaromyces purpurogenus OUCMDZ-019 via disruption of Ash2
Source: Mycology. 2026 May 4;17(3):735–48. doi: 10.1080/21501203.2026.2657145 (PMC13647372; doi:10.1080/21501203.2026.2657145)
Supplement: Supplementary file 1 [file TMYC_A_2657145_SM1946.docx]

Supplemental material

Chromatin-mediated upregulation of *Monascus* pigments in *Talaromyces* *purpurogenus* OUCMDZ-019 via disruption of *Ash2*

Chengfeng Hou^a^^,#^, Deng Yu^a,#^, Yuqing Wang^a^, Peng Fu^a^, Weiming Zhu^a,b,c,*^ and Yi Wang^a,b,c,*^

^a^School of Medicine and Pharmacy, Ocean University of China, Qingdao, China

^b^Laboratory for Marine Drugs and Bioproducts, Qingdao Marine Science and Technology Centre, Qingdao, China

^c^Key Laboratory of Marine Drugs, Ministry of Education of China, School of Medicine and Pharmacy, Ocean University of China, Qingdao, China

^#^Chengfeng Hou and Deng Yu contributed equally to this work.

*Corresponding authors:

Weiming Zhu Email: [weimingzhu@ouc.edu.cn](mailto:weimingzhu@ouc.edu.cn)

Yi Wang [wangyi0213@ouc.edu.cn](mailto:wangyi0213@ouc.edu.cn)

School of Medicine and Pharmacy, Ocean University of China, Qingdao 266003, China

**This file includes:**

Experimental section

Biological activity assay

Figures S1 to S82

Tables S1 to S60

**Supplemental material contents**

**Experimental section**	1

**Biological activity assay**	9

**Figure S1.** Phylogenetic tree of the Ash2 protein.	11

**Figure S2.** *ΔAsh2* knockout vector construction and transformant verification.	12

**Figure S3.** Agarose gel electrophoresis for verification of gene knockout.	13

**Figure S4.** Knockout of *Ash2* significantly upregulated secondary metabolite gene expression.	15

**Figure S5.** Molecular network of the crude extract from the *ΔAsh2* mutant.	17

**Figure S6.** Local BLAST-based identification of the citrinin BGC in OUCMDZ-019.	18

**Figure S7.** ^1^H NMR spectrum of **1** in DMSO-*d_6_* (500 MHz).	19

**Figure S8.** ^13^C NMR spectrum of **1** in DMSO-*d_6_* (125 MHz).	20

**Figure S9**. HSQC spectrum of **1** in DMSO-*d_6_* (500 MHz, 125 MHz).	21

**Figure S10.** HMBC spectrum of **1** in DMSO-*d_6_* (500 MHz, 125 MHz).	22

**Figure S11.** ^1^H-^1^H COSY spectrum of **1** in DMSO-*d_6_* (500 MHz, 500 MHz).	23

**Figure S12.** HRESIMS spectrum of compound **1**.	24

**Figure S13.** IR spectrum of compound **1**.	25

**Figure S14.** ECD spectrum of compound **1**.	26

**Figure S15.** ^1^H NMR spectrum of **2** in DMSO-*d_6_* (400 MHz).	27

**Figure S16.** ^13^C NMR spectrum of **2** in DMSO-*d_6_* (150 MHz).	28

**Figure S17**. HSQC spectrum of **2** in DMSO-*d_6_* (500 MHz, 125 MHz).	29

**Figure S18.** HMBC spectrum of **2** in DMSO-*d_6_* (500 MHz, 125 MHz).	30

**Figure S19.** ^1^H-^1^H COSY spectrum of **2** in DMSO-*d_6_* (500 MHz, 500 MHz).	31

**Figure S20.** HRESIMS spectrum of compound **2**.	32

**Figure S21.** IR spectrum of compound **2**.	33

**Figure S22.** ECD spectrum of compound **2**.	34

**Figure S23.** ^1^H NMR spectrum of **3** in DMSO-*d_6_* (400 MHz).	35

**Figure S24.** ^13^C NMR spectrum of **3** in DMSO-*d_6_* (125 MHz).	36

**Figure S25**. HSQC spectrum of **3** in DMSO-*d_6_* (500 MHz, 125 MHz).	37

**Figure S26.** HMBC spectrum of **3** in DMSO-*d_6_* (500 MHz, 125 MHz).	38

**Figure S27.** ^1^H-^1^H COSY spectrum of **3** in DMSO-*d_6_* (500 MHz, 500 MHz).	39

**Figure S28.** HRESIMS spectrum of compound **3**.	40

**Figure S29.** IR spectrum of compound **3**.	41

**Figure S30.** ^1^H NMR spectrum of **4** in CDCl_3_ (400 MHz).	42

**Figure S31.** ^13^C NMR spectrum of **4** in CDCl_3_ (100 MHz).	43

**Figure S32**. HSQC spectrum of **4** in CDCl_3_ (500 MHz, 125 MHz).	44

**Figure S33.** HMBC spectrum of **4** in CDCl_3_ (500 MHz, 125 MHz).	45

**Figure S34.** ^1^H-^1^H COSY spectrum of **4** in CDCl_3_ (500 MHz, 500 MHz).	46

**Figure S35.** ^1^H NMR spectrum of **5** in CDCl_3_ (500 MHz).	47

**Figure S36.** ^13^C NMR spectrum of **5** in CDCl_3_ (125 MHz).	48

**Figure S37.** ^1^H NMR spectrum of **6** in DMSO-*d_6_* (400 MHz).	49

**Figure S38.** ^13^C NMR spectrum of **6** in DMSO-*d_6_* (125 MHz).	50

**Figure S39**. HSQC spectrum of **6** in DMSO-*d_6_* (500 MHz, 125 MHz).	51

**Figure S40.** HMBC spectrum of **6** in DMSO-*d_6_* (500 MHz, 125 MHz).	52

**Figure S41.** ^1^H-^1^H COSY spectrum of **6** in DMSO-*d_6_* (500 MHz, 500 MHz).	53

**Figure S42.** ^1^H NMR spectrum of **7** in DMSO-*d_6_* (400 MHz).	54

**Figure S43.** ^13^C NMR spectrum of **7** in DMSO-*d_6_* (150 MHz).	55

**Figure S44**. HSQC spectrum of **7** in DMSO-*d_6_* (500 MHz, 125 MHz).	56

**Figure S45.** HMBC spectrum of **7** in DMSO-*d_6_* (500 MHz, 125 MHz).	57

**Figure S46.** ^1^H-^1^H COSY spectrum of **7** in DMSO-*d_6_* (500 MHz, 500 MHz).	58

**Figure S47.** ^1^H-^1^H NOESY spectrum of **7** in DMSO-*d_6_* (500 MHz, 500 MHz)	59

**Figure S48.** ^1^H NMR spectrum of **8** in DMSO-*d_6_* (500 MHz).	60

**Figure S49.** ^13^C NMR spectrum of **8** in DMSO-*d_6_* (150 MHz).	61

**Figure S50**. HSQC spectrum of **8** in DMSO-*d_6_* (500 MHz, 125 MHz).	62

**Figure S51.** HMBC spectrum of **8** in DMSO-*d_6_* (500 MHz, 125 MHz).	63

**Figure S52.** ^1^H-^1^H COSY spectrum of **8** in DMSO-*d_6_* (500 MHz, 500 MHz).	64

**Figure S53.** ^1^H-^1^H NOESY spectrum of **8** in DMSO-*d_6_* (500 MHz, 500 MHz)	65

**Figure S54.** ^1^H NMR spectrum of **9** in CD_3_OD-*d*_4_ (500 MHz).	66

**Figure S55.** ^13^C NMR spectrum of **9** in CD_3_OD-*d*_4_ (150 MHz).	67

**Figure S56**. HSQC spectrum of **9** in CD_3_OD-*d*_4_ (500 MHz, 125 MHz).	68

**Figure S57.** HMBC spectrum of **9** in CD_3_OD-*d*_4_ (500 MHz, 125 MHz).	69

**Figure S58.** ^1^H-^1^H COSY spectrum of **9** in CD_3_OD-*d*_4_ (500 MHz, 500 MHz).	70

**Figure S59.** ^1^H-^1^H NOESY spectrum of **9** in CD_3_OD-*d*_4_ (500 MHz, 500 MHz)	71

**Figure S60.** HRESIMS spectrum of compound **9**.	72

**Figure S61.** IR spectrum of compound **9**.	73

**Figure S62.** ECD spectrum of compound **9**.	74

**Figure S63.** ^1^H NMR spectrum of **10** in CDCl_3_ (600 MHz).	75

**Figure S64.** ^13^C NMR spectrum of **10** in CDCl_3_ (150 MHz).	76

**Figure S65**. HSQC spectrum of **10** in CDCl_3_ (500 MHz, 125 MHz).	77

**Figure S66.** HMBC spectrum of **10** in CDCl_3_ (500 MHz, 125 MHz).	78

**Figure S67.** ^1^H-^1^H COSY spectrum of **10** in CDCl_3_ (500 MHz, 500 MHz).	79

**Figure S68.** ^1^H-^1^H NOESY spectrum of **10** in CDCl_3_ (500 MHz, 500 MHz)	80

**Figure S69.** HRESIMS spectrum of compound **10**.	81

**Figure S70.** IR spectrum of compound **10**.	82

**Figure S71.** ECD spectrum of compound **10**.	83

**Figure S72.** ^1^H NMR spectrum of **11** in DMSO-*d_6_* (400 MHz).	84

**Figure S73.** ^13^C NMR spectrum of **11** in DMSO-*d_6_* (150 MHz).	85

**Figure S74**. HSQC spectrum of **11** in DMSO-*d_6_* (600 MHz, 150 MHz).	86

**Figure S75.** HMBC spectrum of **11** in DMSO-*d_6_* (600 MHz, 150 MHz).	87

**Figure S76.** ^1^H-^1^H COSY spectrum of **11** in DMSO-*d_6_* (600 MHz, 600 MHz).	88

**Figure S77.** ^1^H NMR spectrum of **12** in DMSO-*d_6_* (400 MHz).	89

**Figure S78.** ^13^C NMR spectrum of **12** in DMSO-*d_6_* (150 MHz).	90

**Figure S79**. HSQC spectrum of **12** in DMSO-*d_6_* (500 MHz, 125 MHz).	91

**Figure S80.** HMBC spectrum of **12** in DMSO-*d_6_* (500 MHz, 125 MHz).	92

**Figure S81.** ^1^H-^1^H COSY spectrum of **12** in DMSO-*d_6_* (500 MHz, 500 MHz).	93

**Figure S82.** Raw images for pH stability tests of *Monascus* pigments.	94

**Table S1.** Epigenetic and global regulators in *Talaromyces purpureogenus* OUCMDZ-019	95

**Table S2.** *Tal*-*MrPig* cluster: protein identity and RNA-seq Log_2_FC (*ΔAsh*2/WT).	96

**Table S3.** ^1^H (500 MHz) and ^13^C (125 MHz) NMR data for compound **1** in DMSO-*d*_6_.	97

**Table S4.** ^1^H (400 MHz) and ^13^C (150 MHz) NMR data for compound **2** in DMSO-*d*_6_.	98

**Table S5.** ^1^H (400 MHz) and ^13^C (125 MHz) NMR data for compound **3** in DMSO-*d*_6_.	99

**Table S6.** ^1^H (400 MHz) and ^13^C (100 MHz) NMR data for compound **4** in CDCl_3_.	100

**Table S7.** ^1^H (500 MHz) and ^13^C (125 MHz) NMR data for compound **5** in CDCl_3_.	101

**Table S8.** ^1^H (400 MHz) and ^13^C (125 MHz) NMR data for compound **6** in DMSO-*d*_6_.	102

**Table S9.** ^1^H (400 MHz) and ^13^C (150 MHz) NMR data for compound **7** in DMSO-*d*_6_.	103

**Table S10.** ^1^H (500 MHz) and ^13^C (150 MHz) NMR data for compound **8** in DMSO-*d*_6_.	104

**Table S11.** ^1^H (500 MHz) and ^13^C (150 MHz) NMR data for compound **9** in MeOH-*d*_4_.	105

**Table S12.** ^1^H (600 MHz) and ^13^C (150 MHz) NMR data for compound **10** in CDCl_3_.	106

**Table S13**. ^1^H (400 MHz) and ^13^C (150 MHz) NMR data for compound **11** in DMSO-*d*_6_.	107

**Table S14.** ^1^H (400 MHz) and ^13^C (150 MHz) NMR data for compound **12** in DMSO-*d*_6_.	108

**Table S15.** Culture media compositions used in this study.	109

**Table S16.** Solution compositions used in this study.	110

**Table S17.** Primer information.	111

**Table S18.** Raw data for color value determination.	114

**Table S19.** Raw data for lipid-lowering activity assay (OD at 510 nm).	115

**Table S20.** DFT-optimized structures for low-energy conformers of **1**.	116

**Table S21.** DFT-optimized structures for low-energy conformers of **2**.	117

**Table S22.** DFT-optimized structures for low-energy conformers of **9**.	118

**Table S23.** DFT-optimized structures for low-energy conformers of **10a**.	119

**Table S24.** DFT-optimized structures for low-energy conformers of **10b**.	120

**Table S25.** The Cartesian coordinates of the lowest energy conformers (Conf. A) for **1**.	121

**Table S26.** The Cartesian coordinates of the lowest energy conformers (Conf. B) for **1**.	122

**Table S27.** The Cartesian coordinates of the lowest energy conformers (Conf. C) for **1**.	123

**Table S28.** The Cartesian coordinates of the lowest energy conformers (Conf. D) for **1**.	124

**Table S29.** The Cartesian coordinates of the lowest energy conformers (Conf. E) for **1**.	125

**Table S30.** The Cartesian coordinates of the lowest energy conformers (Conf. A) for **2**.	126

**Table S31.** The Cartesian coordinates of the lowest energy conformers (Conf. B) for **2**.	127

**Table S32.** The Cartesian coordinates of the lowest energy conformers (Conf. C) for **2**.	128

**Table S33.** The Cartesian coordinates of the lowest energy conformers (Conf. D) for **2**.	129

**Table S34.** The Cartesian coordinates of the lowest energy conformers (Conf. E) for **2**.	130

**Table S35.** The Cartesian coordinates of the lowest energy conformers (Conf. F) for **2**.	131

**Table S36.** The Cartesian coordinates of the lowest energy conformers (Conf. G) for **2**.	132

**Table S37.** The Cartesian coordinates of the lowest energy conformers (Conf. H) for **2**.	133

**Table S38.** The Cartesian coordinates of the lowest energy conformers (Conf. A) for **9**.	134

**Table S39.** The Cartesian coordinates of the lowest energy conformers (Conf. B) for **9**.	135

**Table S40.** The Cartesian coordinates of the lowest energy conformers (Conf. C) for **9**.	136

**Table S41.** The Cartesian coordinates of the lowest energy conformers (Conf. D) for **9**.	137

**Table S42.** The Cartesian coordinates of the lowest energy conformers (Conf. E) for **9**.	138

**Table S43.** The Cartesian coordinates of the lowest energy conformers (Conf. F) for **9**.	139

**Table S44.** The Cartesian coordinates of the lowest energy conformers (Conf. G) for **9**.	140

**Table S45.** The Cartesian coordinates of the lowest energy conformers (Conf. A) for **10a**.	141

**Table S46.** The Cartesian coordinates of the lowest energy conformers (Conf. B) for **10a**.	142

**Table S47.** The Cartesian coordinates of the lowest energy conformers (Conf. C) for **10a**.	143

**Table S48.** The Cartesian coordinates of the lowest energy conformers (Conf. D) for **10a**.	144

**Table S49.** The Cartesian coordinates of the lowest energy conformers (Conf. E) for **10a**.	145

**Table S50.** The Cartesian coordinates of the lowest energy conformers (Conf. F) for **10a**.	146

**Table S51.** The Cartesian coordinates of the lowest energy conformers (Conf. G) for **10a**.	147

**Table S52.** The Cartesian coordinates of the lowest energy conformers (Conf. H) for **10a**.	148

**Table S53.** The Cartesian coordinates of the lowest energy conformers (Conf. A) for **10b**.	149

**Table S54.** The Cartesian coordinates of the lowest energy conformers (Conf. B) for **10b**.	150

**Table S55.** The Cartesian coordinates of the lowest energy conformers (Conf. C) for **10b**.	151

**Table S56.** The Cartesian coordinates of the lowest energy conformers (Conf. D) for **10b**.	152

**Table S57.** The Cartesian coordinates of the lowest energy conformers (Conf. E) for **10b**.	153

**Table S58.** The Cartesian coordinates of the lowest energy conformers (Conf. F) for **10b**.	154

**Table S59.** The Cartesian coordinates of the lowest energy conformers (Conf. G) for **10b**.	155

**Table S60.** The Cartesian coordinates of the lowest energy conformers (Conf. H) for **10b**.	156

**References**	157

**Experimental section**

**General experimental procedures**

Optical rotations were recorded with a JASCO P-1020 digital polarimeter (JASCO, Tokyo, Japan). ECD spectra were determined using a JASCO J-815 spectropolarimeter. UV spectra were measured on a Thermofisher NanoDrop OneC microvolume UV-Vis spectrophotometer (Thermo Fisher Scientific, Waltham, MA, USA). IR spectra were performed on a Bruker Tensor-27 spectrophotometer (Bruker, Germany) with KBr discs. NMR spectroscopic data were recorded on an Agilent DD2-500 (Agilent, America) and chemical shifts were referenced to the corresponding residual solvent signals (*δ*_H/C_ 2.50/39.52 for DMSO-*d*_6_). HR-ESI-MS spectral data were collected on a MaXis quadrupole time-of-flight mass spectrometer (Waters, Milford, MA, USA). Low-resolution LC/ESI-MS data were measured using a Waters ACQUITY SQD 2 UPLC/MS system with a reversed-phase C_18_ column (ACQUITY UPLC BEH C_18_, 2.1 mm × 50 mm, 1.7 μm) at a flow rate of 0.4 mL/min. Semipreparative HPLC was performed on a Waters or Hitachi HPLC equipped with a diode array detector (DAD detector) using an ODS column (YMC-pack ODS-A, 10 mm × 250 mm, 5 μm, 2.5/3.0 mL/min), a phenyl column (YMC-pack Ph, 10 mm × 250 mm, 5 μm, 3.0 mL/min) or a PFP column (YMC-pack PFP, 10 mm × 250 mm, 5 μm, 2.5 mL/min). Silica gel (200−300 or 100−200 mesh, Marine Chemical Factory of Qingdao) was used for vacuum-liquid chromatography (VLC). RP-18 silica gel (YMC ODS-A, 50 μm) and Sephadex LH-20 (Amersham Biosciences) were used for column chromatography (CC). The General experimental procedures are described in detail in the Supporting Information.

**Fungal material**

The isolation and identification of the fungus *Talaromyces purpurogenum* OUCMDZ-019, derived from Jianshui red soil, have been previously described (GenBank accession No. HQ434765). (Wang et al. 2011) Initially, it was identified as *Penicillium purpurogenum* JS03-21; (Wang et al. 2011, 2013; Hou et al. 2025) owever, recent comprehensive literature reviews (Frisvad et al. 2013) and NCBI BLAST analysis have confirmed its correct classification as *T*. *purpureogenus* OUCMDZ-019. The fungus was deposited in our Lab, OUC, China. The working strain was cultured on potato dextrose agar slants (PDA) and stored at 4 °C.

Genomic DNA was extracted from the mycelia of *T*. *purpureogenus* OUCMDZ-019 strain grown in PDB after 5 days. The whole genome of *T*. *purpureogenus* OUCMDZ-019 was sequenced using Illumina HiSeq PE150 at Beijing Allwegene Technology Co., Ltd.

**Bioinformatics analysis**

Analysis of secondary metabolite biosynthesis gene clusters in the genome of *T*. *purpureogenus* OUCMDZ-019 was performed by antiSMASH 5.0 fungal version. Nucleic acid and amino acid sequence homology and function were analyzed using the BLAST engine from the National Center for Biotechnology Information (NCBI) (<https://blast.ncbi.nlm.nih.gov/Blast.cgi>). Multiple sequence alignments were performed by DNAMAN. Protein domains were predicted using InterPro program (<https://www.ebi.ac.uk/interpro/>) and Conserved Domain Database (CCD) search of the NCBI ([http://www.ncbi.nlm.nih.gov/Structure /cdd/wrpsb.cgi](http://www.ncbi.nlm.nih.gov/Structure%20/cdd/wrpsb.cgi)).

**Phylogenetic tree construction**

Amino acid sequences retrieved from NCBI were aligned using ClustalW implemented in MEGA11. Phylogenetic analysis of representative proteins was performed in MEGA11 using the neighbor-joining (NJ) method. The reliability of the resulting tree was assessed by bootstrap analysis with 1000 replicates.

**General molecular biology methods**

Genomic DNA (gDNA) from the wild-type fungus *T*. *purpurogenus* OUCMDZ-019 was prepared using OMEGA Fungal DNA Kit (Omega Bio-tek) according to the manufacturer’s protocol. Polymerase chain reactions for cloning were performed using PrimeSTAR ^®^ HS premix (Takara). T4 DNA Ligase (Thermo Scientific™) was responsible for ligation of the enzyme digested linear genes and vectors. The plasmids for gene inactivation were constructed by yeast homologous recombination and rescued using Zymoprep Yeast Miniprep Kit (Zymo Research). Plasmids in *E. coli* were extracted using the OMEGA Plasmid Mini Kit I (Omega Bio-tek).

**Construction of knockout mutant strains**

*T*. *purpureogenus* OUCMDZ-019 gDNA was used as a template to amplify the upstream and downstream homology arm fragments flanking the target gene *Ash2* (*026*), using the primer pairs 026-1-F/R and 026-2-F/R, respectively. The *neo*-resistant fragment (G-418) was amplified from the pMD19-T-*neo* plasmid using primers 026-G418-F/R. The *Ash2* knockout plasmid was constructed by inserting the fragment (containing homology arms and the *neo*-resistant fragment) into the linearized pMD19T vector using seamless cloning (*pEASY* ^®^-Basic Seamless Cloning and Assembly Kit), named pMD19T-*Ash2*-KO. The recombinant pMD19T-*Ash2*-KO plasmid was transformed into *E. coli* DH5α via the hot-punch method. The genetic testing for the fusion fragment 026-1 + G-418 + 026-2 amplified by the primers M13-F/R was performed to verify the successful construction of the knockout plasmid. The deletion cassette fragments were amplified from the pMD19T-*Ash2*-KO plasmid using primer pairs 026-1 + G418-F/R and G418 + 026-2-F/R.

*T*. *purpureogenus* OUCMDZ-019 was cultured in a PDA medium for 3–4 days at 28 °C. Rinse the spore with sterile water to obtain a spore suspension. 5 mL of the spore suspension was added to 150 mL of SDYS media and incubated at 180 r/min in a shaker at 28 °C. Approximately 12–13 h later, the mycelial length reached 3 times the spore diameter. After centrifuging the culture medium, obtain the mycelium. Resuspend the mycelium in TF_1_ solution and centrifuge twice, and then re-suspended in 5 mL TF_2_ enzyme (Yatalase, TaKara) solution and incubated at 30°C, 100 r/min for 1 h. Filter the enzymatic digestion solution through Miracloth filter cloth, and lowly add 1 mol/L sorbitol solution to rinse the Miracloth filter cloth, yielding a protoplast suspension. The protoplasts were collected by filtration and centrifuged at 4 °C, 3000 g, 10 min. The protoplasts were washed with STC solution and centrifuged twice, subsequently resuspended in STC solution to a concentration of 10⁸ protoplasts/mL. Add 1% DMSO to the protoplasts, aliquot them, and store at –80 °C refrigerator. 2–5 μg of purified linear deletion cassette fragments were mixed with 100 μL protoplasts and incubated on ice for 20 min. PTC solution was slowly added to the mixture and incubated at room temperature for 20 min. Slowly add the protoplast suspension to 10 mL hypertonic PDB medium and incubate on a shaker at 28 °C and 100 r/min for approximately 11 h. Resuspend the protoplast regenerates obtained by centrifugation at 4000 r/min for 10 min in STC solution. Subsequently, spread them onto hypertonic *neo*-resistance (500 μg/mL) PDA medium and culture them in a 28 °C incubator for 4–6 d.

The genomic DNA of the positive selected transformant was further verified through diagnostic PCR. The 2.69 kb and 2.72 kb (*neo* gene) fragments could be amplified from the correct *ΔAsh2* strain using the primers wai + 026-1 + G418-F/R and G-418-F/R, respectively, but were absent in the WT. No PCR product was amplified from the *ΔAsh2* strain using primers 026-yz-F/R, while a 1.1 kb product (Part of the *Ash2* gene fragment) could appear using the genomic DNA of the WT. Fragments of 4.8 kb and 4.1 kb were amplified from the *ΔAsh2* and WT strains, respectively, using primers 026-1-F/026-2-R. The above diagnostic PCR results confirmed that the target *Ash2* gene was successfully replaced at the correct locus with the *neo* resistance gene, with no evidence of random insertion.

**Transcriptome sequencing of OUCMDZ-019 WT and the *ΔAsh*2 mutant**

Spores of *T. purpurogenus* OUCMDZ-019 WT and the *ΔAsh*2 mutant were inoculated into acidified PDB medium (pH ~2.2) and cultivated at 28 °C and 180 r/min for 6 d. The mycelia were collected by filtration through gauze and repeatedly washed with sterile ddH_2_O to remove residual medium and impurities. The cleaned mycelia were transferred into sterile 1.5 mL centrifuge tubes, rapidly frozen in liquid nitrogen, and immediately submitted to Majorbio (Shanghai, China) for transcriptome sequencing.

**Construction of GNPS molecular network for the *ΔAsh*2 crude extract**

The crude extract of the *ΔAsh2* strain was dissolved in HPLC-grade methanol to a final concentration of 1.0 mg/mL and filtered through a 0.22 μm PTFE syringe filter prior to analysis. LC-MS/MS data were acquired on a Waters 2695 HPLC system coupled to an amaZon SL ion-trap mass spectrometer (Bruker). Chromatographic separation was performed on a YMC ODS-A column (250 mm × 4.6 mm, 5 μm) at ambient temperature. The injection volume was 10 μL and the flow rate was set to 1.0 mL/min. The mobile phase consisted of methanol (A) and water containing 0.1% formic acid (B). A linear gradient was applied as follows: 0–20 min, 10%–100% A; 21–25 min, 100% A.

Mass spectrometric detection was carried out in positive electrospray ionization (ESI+) mode with an m/z scan range of 100–1000. MS/MS spectra were collected using automated data-dependent acquisition (auto-MS/MS), in which precursor ions detected in the full scan were sequentially selected for fragmentation. The acquired MS/MS data were converted to mzXML format using MSConvert (ProteoWizard) and uploaded to the Global Natural Products Social Molecular Networking (GNPS) platform for molecular networking analysis. Molecular networks were generated in GNPS using the classical molecular networking workflow with standard spectral filtering and cosine-based spectral similarity scoring; only edges above the predefined similarity threshold and minimum matched fragment ions were retained. The resulting network files were exported from GNPS and visualized in Cytoscape (version 3.7.1).

**Fermentation, Extraction and Purification**

The *ΔAsh2* strains were cultivated in the potato dextrose broth (PDB) medium adjusted to pH 2.2. The cultures were inoculated with fresh spores in 150 × 500 mL Erlenmeyer flasks containing 150 mL of PDB medium (pH 2.2) and grown under shaking condition at 180 r/min, 28 ℃ for 12 d. The fermentation broth was extracted repeatedly with an equivalent volume of ethyl acetate (EtOAc) five times, and the combined organic solvent was evaporated to dryness under vacuum to yield the crude extract of *ΔAsh2* mutant (20.0 g).

The extract was subjected to a VLC separation on a silica gel column using step gradient elution with petroleum (100%) and MeOH-CH_2_Cl_2_ (0−100%) to give 8 fractions (Fr. 1−Fr. 8). Fr. 2 (2.6 g) was fractionated into 11 fractions (Fr. 2.1−Fr. 2.11) by an RP-silica gel column eluting with a stepwise gradient of MeOH-H_2_O (10%−100%). Fr. 2.5 was subjected to a Sephadex LH-20 column eluting with MeOH and further purified by semipreparative HPLC on an ODS column (YMC-pack ODS-A, 10 mm × 250 mm, 5 μm, 3.0 mL/min) eluting with 45% MeCN-H_2_O (0.5‰ TFA) to yield compound **10** (2.1 mg, *t*_R_ = 16.3 min). Fr. 2.6 was subjected to a Sephadex LH-20 column eluting with MeOH and further purified by semipreparative HPLC on the same ODS column eluting with 52% MeCN-H_2_O (0.5‰ TFA) to yield compound **6** (4.5 mg, *t*_R_ = 12.2 min) and compound **9** (5.5 mg, *t*_R_ = 15.5 min). Fr. 2.7 was subjected to a Sephadex LH-20 column eluting with MeOH and further purified by semipreparative HPLC on a phenyl column (YMC-pack Ph, 10 mm × 250 mm, 5 μm, 3.0 mL/min) eluting with 48% MeCN-H_2_O (0.5‰ TFA) to obtain compound **8** (4.0 mg, *t*_R_ = 15.5 min). Fr. 2.9 was subjected to a Sephadex LH-20 column eluting with MeOH and further purified by semipreparative HPLC on the same phenyl column eluting with 55% MeCN-H_2_O (0.5‰ TFA) to obtain compound **7** (6.0 mg, *t*_R_ = 16.0 min). Fr. 2.10 was subjected to a Sephadex LH-20 column eluting with MeOH and further purified by semipreparative HPLC on the same ODS column (2.5 mL/min) eluting with 64% MeCN-H_2_O (0.5‰ TFA) to obtain compound **4** (3.5 mg, *t*_R_ = 20.5 min). Fr. 2.11 was subjected to a Sephadex LH-20 column eluting with MeOH and further purified by semipreparative HPLC on the same ODS column (2.5 mL/min) eluting with 68% MeCN-H_2_O (0.5‰ TFA) to yield compound **5** (2.6 mg, *t*_R_ = 16.8 min).

Fr. 4 (0.75 g) was fractionated into 8 fractions (Fr. 4.1−Fr. 4.8) by an RP-silica gel column eluting with a stepwise gradient of MeOH-H_2_O (10%−100%). Fr. 4.1 was subjected to a Sephadex LH-20 column eluting with MeOH and further purified by semipreparative HPLC on a PFP column (YMC-pack PFP, 10 mm × 250 mm, 5 μm, 2.5 mL/min) eluting with 40% MeCN-H_2_O (0.5‰ TFA) to yield compound **12** (2.3 mg, *t*_R_ = 16.6 min). Fr. 4.4 was subjected to a Sephadex LH-20 column eluting with MeOH and further purified by semipreparative HPLC on the same ODS column (2.5 mL/min) eluting with 62% MeCN-H_2_O (0.5‰ TFA) to yield compound **1** (6.5 mg, *t*_R_ = 16.0 min). Fr. 4.5 was subjected to a Sephadex LH-20 column eluting with MeOH and further purified by semipreparative HPLC on the same ODS column (2.5 mL/min) eluting with 15% MeCN-H_2_O (0.5‰ TFA) to yield compound **11** (5.4 mg, *t*_R_ = 22.5 min). Fr. 6 (2.0 g) was fractionated into 7 fractions (Fr. 6.1−Fr. 6.7) by an RP-silica gel column eluting with a stepwise gradient of MeOH-H_2_O (10−100%). Fr. 6.7 was subjected to a Sephadex LH-20 column eluting with MeOH and further purified by semipreparative HPLC on the same ODS column (2.5 mL/min) eluting with 57% MeCN-H_2_O (0.5‰ TFA) to obtain compound **2** (1.4 mg, *t*_R_ = 15.3 min). Fr. 7 (3.23 g) was fractionated into 8 fractions (Fr. 7.1−Fr. 7.8) by an RP-silica gel column eluting with a stepwise gradient of MeOH−H_2_O (10−100%). Fr. 7.6 was subjected to a Sephadex LH-20 column eluting with MeOH and further purified by semipreparative HPLC on the same PFP column eluting with 45% MeCN-H_2_O (0.5‰ TFA) to obtain compound **3** (3.0 mg, *t*_R_ = 14.9 min).

**Structural Elucidation of Compounds**

*Talarpurpurones A* *(****1****)*: red-black amorphous powder; [*α*]25 D −655 (*c* 0.002, MeOH); UV (MeOH) *λ*_max_ (log *ε*) 200 (2.04), 272 (1.20), 425 (1.32), 515 (2.06) nm; ECD (0.94 mmol/L, MeOH) *λ*_max_ (Δ*ε*) 228 (−4.53), 273 (+17.04), 343 (−2.50), 409 (+0.79), 519 (−14.76) nm; IR (KBr) *v*_max_ 3424, 2927, 2855, 1717, 1632, 1461, 1319, 1200, 1150, 1095, 1020, 721 cm^−1^; ^1^H and ^13^C NMR, see Table S3; HRESIMS *m*/z 532.1979 [M + H]^+^ (calcd. for C_30_H_30_NO_8_, 532.1966)

*Talarpurpurones B (****2****)*: red-black amorphous powder; [*α*]25 D −8680 (*c* 0.0005, MeOH); UV (MeOH) *λ*_max_ (log *ε*) 200 (2.05), 272 (1.23), 425 (1.33), 502 (1.66) nm; ECD (1.1 mmol/L, MeOH) *λ*_max_ (Δ*ε*) 229 (−0.97), 275 (+20.20), 332 (−2.85), 416 (+1.25), 511 (−12.0) nm; IR (KBr) *v*_max_ 3567, 2926, 2854, 1715, 1628, 1543, 1463, 1207, 1147, 726 cm^−1^; ^1^H and ^13^C NMR, see Table S4; HRESIMS *m/z* 456.2006 [M + H]^+^ (calcd. for C_25_H_30_NO_7_, 456.2017)

*Atrorosin S (****3****)*: red-black amorphous powder; [*α*]25 D −4980 (*c* 0.0005，MeOH); UV (MeOH) *λ*_max_ (log *ε*) 200 (1.95), 271 (1.08), 425 (1.21), 499 (1.94) nm; ECD (1.0 mmol/L, MeOH) *λ*_max_ (Δ*ε*) 227 (−0.13), 275 (+22.38), 331 (−3.23), 407 (+1.74), 519 (−15.89) nm; IR (KBr) *v*_max_ 3561, 2923, 2856, 1716, 1630, 1541, 1464, 1213, 1148 cm^−1^; ^1^H and ^13^C NMR, see Table S5; HRESIMS *m/z* 500.1904 [M + H]^+^ (calcd. for C_26_H_30_NO_9_, 500.1915)

*PP-O (****4****)*: orange-black amorphous powder; [*α*]25 D −4980 (*c* 0.0005，MeOH); ^1^H and ^13^C NMR, see Table S6; ESIMS *m/z* 413.26 [M + H]^+^ (calcd .for C_23_H_25_O_7_, 413.28)

*Talaralbol B (****5****)*: orange-black amorphous powder; ^1^H and ^13^C NMR, see Table S7; ESIMS *m/z* 413.26 [M + H]^+^ (calcd. for C_23_H_25_O_7_, 413.28)

*Monasfluol B (****6****)*: Yellow amorphous powder; [*α*]25 D −43 (*c* 0.001, MeOH); ^1^H and ^13^C NMR, see Table S8; ESIMS *m/z* 403.26 [M + H]^+^ (calcd. for C_23_H_25_O_7_, 403.26)

*Talaralbol A (****7****)*: Yellow amorphous powder; ECD (1.2 mmol/L, MeOH) *λ*_max_ (Δ*ε*) 225 (+0.76), 238 (+0.02), 256 (+2.23), 289 (−0.95), 308 (−0.46), 335 (−1.01), 405 (+4.45) nm; ^1^H and ^13^C NMR, see Table S9; HRESIMS *m*/*z* 417.1914 [M + H]^+^ (calcd. for C_23_H_29_NO_7_, 417.1908)

*Sequoiamonascin A (****8****)*: Yellow amorphous powder; [*α*]25 D −33 (*c* 0.001, MeOH); ^1^H and ^13^C NMR, see Table S10; HRESIMS *m/z* 419.2072 [M + H]^+^ (calcd. for C_23_H_31_NO_7_, 419.2064)

*Talarpurpurones C (****9****)*: yellow amorphous powder; [*α*]25 D −162 (*c* 0.001, MeOH), UV (MeOH) *λ*_max_ (log *ε*) 228 (2.05), 387 (1.23) nm; ECD (1.3 m, MeOH) *λ*_max_ (Δ*ε*) 221 (−5.73), 258 (+2.49), 338 (−2.29), 401 (+6.06) nm; IR (KBr) *v*_max_ 3646, 3399, 2926, 2855, 1702, 1639, 1540, 1458, 1399, 1261, 1032, 805 cm^−1^; ^1^H and ^13^C NMR, see Table S11; HRESIMS *m/z* 391.2006 [M + H]^+^ (calcd. for C_22_H_31_O_6_, 391.2017)

*Talarpurpurones D (****10****)*: colorless oil; [*α*]25 D +122.9 (*c* 0.1, MeOH); UV (MeOH) *λ*_max_ (log *ε*) 214 (0.31), 247 (0.99), 321 (0.05) nm; ECD (1.2 mmol/L, MeOH) *λ*_max_ (Δ*ε*) 215 (+1.40), 246 (+7.95), 285 (−1.36), 322 (+0.65), 383 (−0.05) nm; IR (KBr) *v*_max_ 3646, 3359, 2926, 2855, 1778, 1716, 1699, 1650, 1540, 1380, 1078, 799 cm^−1^; ^1^H and ^13^C NMR, see Table S12; HRESIMS *m/z* 421.2218 [M + H]^+^ (calcd. for C_23_H_33_O_7_, 421.2221)

*Monascusone A (****11****)*: yellow amorphous powder; ^1^H and ^13^C NMR, see Table S13; ESIMS *m/z* 255.56 [M + H]^+^ (calcd. for C_13_H_18_O_5_, 255.58)

*FK17-P2b1 (****12****)*: yellow amorphous powder; ^1^H and ^13^C NMR, see Table S14; ESIMS *m/z* 237.04 [M + H]^+^ (calcd. for C_13_H_18_O_5_, 237.10)

**Electronic circular dichroism (ECD) calculations**

Conformational searches were performed in Spartan’14 using the MMFF force field with an energy window of 10 kJ/mol to generate a set of low-energy conformers. All candidate conformers were then re-optimized in Gaussian 16 at the B3LYP/6-31G(d) level with implicit methanol solvation (SCRF, PCM) using the Opt Freq protocol. Frequency calculations were carried out at the same level to confirm that each optimized structure corresponded to a true minimum (no imaginary frequencies) and to obtain the thermochemical data used for Boltzmann population analysis.

Time-dependent density functional theory (TDDFT) ECD calculations were subsequently conducted in Gaussian 16 at the B3LYP/6-31G(d) level in methanol (SCRF, PCM) to obtain excitation energies and rotational strengths for the optimized conformers. The ECD spectra of individual conformers were generated in GaussView and Boltzmann-weighted according to their relative Gibbs free energies. The final simulated spectra were produced by applying Gaussian band broadening with a half-height bandwidth (σ) of 0.35 eV. The resulting calculated ECD curves were plotted in Origin 2021 and compared with the experimental ECD spectra to assign the absolute configurations of the chiral centers.

**Azaphilic addition assay for** **orange *Monascus* pigments**

Optimization of azaphilic (aminophilic) addition conditions for orange *Monascus* pigments was performed as follows: 0.1 mg of the target compound and 0.1 mg of _L_-serine were reacted under varying conditions, including different pH values, reaction times, and reaction solvents, to verify azaphilic addition. The external reaction conditions were kept consistent with the *ΔAsh2* fermentation conditions (28 °C, 180 r/min). After completion, the reaction mixtures were analyzed by LC-MS to assess product formation and conversion.

**Biological activity assay**

**Color value measurement and calculation method**

The color values of the compounds were determined by UV-Vis spectroscopy at the maximum absorption wavelength (λ_max_) for each compound. The λ_max_ values of the compounds were measured between 386 and 517 nm (Table S18). The color value (*E*) was calculated using the following formula:

$$E_{1cm}^{1\%}(\lambda_{max})=\frac{A\times f}{m\times100}$$

where *A* is the absorbance measured at the maximum absorption wavelength (λ_max_) using a 1 cm path length, *f* is the dilution factor (40 or 80), and *m* is the sample mass in grams. The absorbance was measured at three different wavelengths (Table S18), and the average was used to calculate the color value. The color values were expressed in U/g and represent the light absorbance capacity per gram of compound at the maximum absorption wavelength. The final color value (*E*) for each compound was calculated based on a concentration of 1 mg/mL solution, prepared by dissolving the compound in an appropriate solvent and diluting as necessary to achieve measurable absorbance.

For calculation, the sample mass was weighed accurately, and the solutions were prepared using appropriate solvents. All samples were measured in triplicate, and the average value was used for color value calculations. The λ_max_ values were determined from UV-Vis spectra, and the final color values were reported in units of *E* (U/g).

**pH stability testing method**

First, 0.5 mg of MPs was dissolved in 500 μL of 40% methanol aqueous solution, and the solution was adjusted to an absorbance of OD_λmax_ = 2.0 ± 0.1 at the maximum absorption wavelength (λ_max_). Then, 100 μL of the solution was taken and mixed with the corresponding buffer: citric acid-phosphate buffer (pH 3.0, pH 5.0, or pH 7.0), sodium carbonate-sodium bicarbonate buffer (pH 9.0, pH 11.0), bringing the total volume to 1 mL per tube. All samples were incubated at 25 °C in the dark for 24 h.

During the experiment, the color changes of MPs under different pH conditions were measured using a TU-1900 UV-Vis spectrophotometer (Presee, Beijing, China), and their UV characteristics were recorded. Commercial red *Monascus* pigment was used as a control and treated under the same conditions to compare stability changes.

**Lipid-lowering activity assay**

Lipid-lowering activity was evaluated using a cell-free oleic acid/palmitic acid (OA/PA) model and an Oil Red O–based colorimetric readout at 510 nm. OA and PA were prepared as stock solutions and mixed to generate the lipid model solution (OA + PA). The assay included a blank control (vehicle only), a model control (OA + PA), positive controls [OA + PA + simvastatin (SIM) or rosiglitazone (ROS)], and test groups (OA + PA + the indicated concentrations of compounds **1**–**3**, **7**, and **9**). After incubation, Oil Red O working solution was added to each well and allowed to stain; excess dye was removed and wells were washed. Bound dye was then eluted with isopropanol, and absorbance was measured at 510 nm using a microplate reader. The lipid-lowering rate was calculated as: lipid-lowering rate (%) = [1 − (OD_sample_ − OD_blank_)/(OD_model_ − OD_blank_)] × 100. All measurements were performed in triplicate.

**Antimicrobial assay**

The antimicrobial activities against *Staphylococcus aureus* subsp. *aureus* ATCC 6538, methicillin-resistant *Staphylococcus aureus* (MRSA) ATCC 43300, *Bacillus subtilis* ATCC 6633, *Escherichia coli* ATCC 11775, and *Pseudomonas aeruginosa* ATCC 10145 were evaluated by the broth microdilution method. The tested strains were cultivated in LB (for bacteria) or YPD (for fungi). Briefly, two-fold serial dilutions of the test compounds were prepared in LB medium and inoculated with bacterial suspensions to a final density of 5 × 10^5^ CFU/mL. After incubation, the minimum inhibitory concentration (MIC) was defined as the lowest compound concentration that completely inhibited visible bacterial growth. All MIC determinations were performed in triplicate for each strain, and ciprofloxacin was used as the positive control.

Figure S1. Phylogenetic tree of the Ash2 protein.

Figure S2. *ΔAsh2* knockout vector construction and transformant verification.

Construction of the *ΔAsh2* knockout vector, homologous recombination, and verification of transformants. (a) Schematic illustration of the construction strategy for the *Ash2* knockout plasmid (pMD19T-*Ash2*-KO). (b) Schematic overview of the double cross-over homologous recombination strategy for gene replacement. (c) Primer design and PCR-amplification scheme used to verify transformants/knockout strains. (d) Agarose gel electrophoresis of the upstream and downstream homology arms and the *neo* (G418) resistance cassette. (e) Colony PCR verification of the recombinant plasmid in *Escherichia coli*. (f) Diagnostic PCR verification of the *Ash2* deletion mutant (M, DNA marker). Lanes 1, 3, 5, and 7 used *ΔAsh2* genomic DNA as the template, lanes 2, 4, 6, and 8 used wild-type (WT) genomic DNA as the template, and lanes 9 and 10 were water blanks.

**Figure S3.** Agarose gel electrophoresis for verification of gene knockout.

Diagnostic PCR verification of gene-deletion mutants by agarose gel electrophoresis. (A and B) Verification of *ΔHosA* (6689) (a) and *ΔHosB* (*407*) (b) transformants (lanes 1–14). Every two lanes represent one set consisting of the deletion mutant and the wild type (WT), with odd-numbered lanes corresponding to the mutant and even-numbered lanes to WT. Lanes 1–2 verify amplification using an outer primer with the upstream homologous arm and a partial *neo* (*G418*) fragment; lanes 3–4 verify the full-length *G418* gene; lanes 5–6 verify the upstream homologous arm and a fragment of the target gene; lanes 7–8 verify a fragment of the target gene; lanes 9–10 verify the target gene and the downstream homologous arm; lanes 11–12 verify the three-fragment products (upstream arm–target gene or *G418*–downstream arm); and lanes 13–14 are water blanks. (c and e) Verification of *ΔLaeA* (*8155*) (c) and *ΔAsh2* (*026*) (e) transformants (lanes 1–10). Every two lanes represent one set (odd lanes, mutant; even lanes, WT): lanes 1–2 verify an outer primer with the upstream arm and a partial *G418* fragment; lanes 3–4 verify the full-length *G418* gene; lanes 5–6 verify a fragment of the target gene; lanes 7–8 verify the three-fragment products (upstream arm–target gene or *G418*–downstream arm); and lanes 9–10 are water blanks. (d) Verification of *ΔHdaA* (*5375*) transformants (lanes 1–12). Every three lanes represent one set consisting of two mutant replicates (first two lanes) and one WT control (third lane): lanes 1–3 verify an outer primer with the upstream arm and a partial *G418* fragment; lanes 4–6 verify the full-length *G418* gene; lanes 7–9 verify a fragment of the target gene; and lanes 10–12 verify the three-fragment products (upstream arm–target gene or *G418*–downstream arm). M: DNA marker; primer combinations are indicated in the panels.

Figure S4. Knockout of *Ash2* significantly upregulated secondary metabolite gene expression.

Knockout of *Ash2* significantly upregulated secondary metabolite gene expression. (a) Numbers of the significantly upregulated (Log_2_FC > 1) core biosynthetic genes from various BGC types between *ΔAsh2* and WT. The results indicated that the deletion of *Ash2* led to significant upregulations of most core biosynthetic genes of BGCs. (b) Statistics of the top five genes with the highest Log_2_FC values for core biosynthetic genes in different types of BGCs between *ΔAsh2* and WT. The core biosynthetic genes for red MPs exhibited the highest Log_2_FC value, reflecting the significant upregulation of their BGCs. (c and d) UPLC chromatograms of the extracts from OUCMDZ-019 strains detected at 254 nm. (c) Comparison between WT and the *ΔLaeA* mutant; (d) Comparison between WT and the *ΔAsh2* mutant. Red dashed lines indicated the differential metabolites between the indicated strains.

Figure S5. Molecular network of the crude extract from the *ΔAsh2* mutant.

Global GNPS molecular network and a zoomed-in view of the crude extract from the *ΔAsh2* mutant fermented in acidified PDB. MPs-related metabolites (*Monascus* pigments, MPs) formed a distinct, densely populated cluster in the network, indicating that *Ash2* deletion markedly enriched and expanded the MPs metabolite profile in this strain. The MPs chemical structures corresponding to key nodes in the molecular network were presented.

**Figure S6.** Local BLAST-based identification of the citrinin BGC in OUCMDZ-019.

The reported citrinin biosynthetic gene cluster and its biosynthetic pathway (He and Cox 2016).

| ***Cit*** | **Protein in OUCMDZ-019** | **Index (%)** | ***Cit*** | **Protein in OUCMDZ-019** | **Index (%)** |
| --- | --- | --- | --- | --- | --- |
| Cit A | OUCMDZ-0193510-PA | 50.38 | Cit D | OUCMDZ-0196120-PA | 37.77 |
|  | OUCMDZ-0196996-PA | 51.75 |  | OUCMDZ-0195224-PA | 39.34 |
|  | OUCMDZ-0193595-PA | 49.07 |  | OUCMDZ-0197514-PA | 35.23 |
|  | OUCMDZ-0194254-PA | 27.47 |  | OUCMDZ-0198184-PA | 38.01 |
|  | OUCMDZ-0199783-PA | 26.62 |  | OUCMDZ-0197222-PA | 36.83 |
| Cit B | OUCMDZ-01910487-PA | 60.25 | Cit E | OUCMDZ-0197464-PA | 57.39 |
|  | OUCMDZ-019824-PA | 50.32 |  | OUCMDZ-0195576-PA | 48.80 |
|  | OUCMDZ-0196007-PA | 35.53 |  | OUCMDZ-019867-PA | 48.59 |
|  | OUCMDZ-0196286-PA | 32.88 |  | OUCMDZ-0196455-PA | 47.31 |
|  | OUCMDZ-019884-PA | 30.86 |  | OUCMDZ-0193517-PA | 47.35 |
| Cit C | OUCMDZ-0198518-PA | 33.92 | Cit S | OUCMDZ-0193596-PA | 49.14 |
|  | OUCMDZ-0193939-PA | 34.58 |  | OUCMDZ-0198745-PA | 34.64 |
|  | OUCMDZ-0196317-PA | 35.50 |  | OUCMDZ-0196995-PA | 33.67 |
|  | OUCMDZ-01910162-PA | 30.03 |  | OUCMDZ-01910480-PA | 37.02 |
|  | OUCMDZ-0192817-PA | 29.41 |  | OUCMDZ-019435-PA | 35.55 |

Local BLAST-based search for the citrinin BGC in *Talaromyces* *purpureogenus* OUCMDZ-019. The table displayed the top five genes with the highest homology to each citrinin BGC gene in strain OUCMDZ-019. Compared with the reported citrinin BGC, only scattered genes exhibiting low homology were detected in strain OUCMDZ-019. Crucially, these genes were not organized into a contiguous cluster. These results indicated that *T*. *purpureogenus* OUCMDZ-019 lacks a complete citrinin BGC and is therefore unable to biosynthesize citrinin.

Figure S7. ^1^H NMR spectrum of 1 in DMSO-*d_6_* (500 MHz).

**Figure S8.** ^13^C NMR spectrum of **1** in DMSO-*d_6_* (125 MHz).

**Figure S9**. HSQC spectrum of **1** in DMSO-*d_6_* (500 MHz, 125 MHz).

**Figure S10.** HMBC spectrum of **1** in DMSO-*d_6_* (500 MHz, 125 MHz).

**Figure S11.** ^1^H-^1^H COSY spectrum of **1** in DMSO-*d_6_* (500 MHz, 500 MHz).

**Figure S12.** HRESIMS spectrum of compound **1**.

**Figure S13.** IR spectrum of compound **1**.

**Figure S14.** ECD spectrum of compound **1**.

Figure S15. ^1^H NMR spectrum of 2 in DMSO-*d_6_* (400 MHz).

**Figure S16.** ^13^C NMR spectrum of **2** in DMSO-*d_6_* (150 MHz).

**Figure S17**. HSQC spectrum of **2** in DMSO-*d_6_* (500 MHz, 125 MHz).

**Figure S18.** HMBC spectrum of **2** in DMSO-*d_6_* (500 MHz, 125 MHz).

**Figure S19.** ^1^H-^1^H COSY spectrum of **2** in DMSO-*d_6_* (500 MHz, 500 MHz).

**Figure S20.** HRESIMS spectrum of compound **2**.

**Figure S21.** IR spectrum of compound **2**.

**Figure S22.** ECD spectrum of compound **2**.

Figure S23. ^1^H NMR spectrum of 3 in DMSO-*d_6_* (400 MHz).

**Figure S24.** ^13^C NMR spectrum of **3** in DMSO-*d_6_* (125 MHz).

**Figure S25**. HSQC spectrum of **3** in DMSO-*d_6_* (500 MHz, 125 MHz).

**Figure S26.** HMBC spectrum of **3** in DMSO-*d_6_* (500 MHz, 125 MHz).

**Figure S27.** ^1^H-^1^H COSY spectrum of **3** in DMSO-*d_6_* (500 MHz, 500 MHz).

**Figure S28.** HRESIMS spectrum of compound **3**.

**Figure S29.** IR spectrum of compound **3**.

Figure S30. ^1^H NMR spectrum of 4 in CDCl_3_ (400 MHz).

**Figure S31.** ^13^C NMR spectrum of **4** in CDCl_3_ (100 MHz).

**Figure S32**. HSQC spectrum of **4** in CDCl_3_ (500 MHz, 125 MHz).

**Figure S33.** HMBC spectrum of **4** in CDCl_3_ (500 MHz, 125 MHz).

**Figure S34.** ^1^H-^1^H COSY spectrum of **4** in CDCl_3_ (500 MHz, 500 MHz).

Figure S35. ^1^H NMR spectrum of 5 in CDCl_3_ (500 MHz).

**Figure S36.** ^13^C NMR spectrum of **5** in CDCl_3_ (125 MHz).

Figure S37. ^1^H NMR spectrum of 6 in DMSO-*d_6_* (400 MHz).

**Figure S38.** ^13^C NMR spectrum of **6** in DMSO-*d_6_* (125 MHz).

**Figure S39**. HSQC spectrum of **6** in DMSO-*d_6_* (500 MHz, 125 MHz).

**Figure S40.** HMBC spectrum of **6** in DMSO-*d_6_* (500 MHz, 125 MHz).

**Figure S41.** ^1^H-^1^H COSY spectrum of **6** in DMSO-*d_6_* (500 MHz, 500 MHz).

Figure S42. ^1^H NMR spectrum of 7 in DMSO-*d_6_* (400 MHz).

**Figure S43.** ^13^C NMR spectrum of **7** in DMSO-*d_6_* (150 MHz).

**Figure S44**. HSQC spectrum of **7** in DMSO-*d_6_* (500 MHz, 125 MHz).

**Figure S45.** HMBC spectrum of **7** in DMSO-*d_6_* (500 MHz, 125 MHz).

**Figure S46.** ^1^H-^1^H COSY spectrum of **7** in DMSO-*d_6_* (500 MHz, 500 MHz).

**Figure S47.** ^1^H-^1^H NOESY spectrum of **7** in DMSO-*d_6_* (500 MHz, 500 MHz)

Figure S48. ^1^H NMR spectrum of 8 in DMSO-*d_6_* (500 MHz).

**Figure S49.** ^13^C NMR spectrum of **8** in DMSO-*d_6_* (150 MHz).

**Figure S50**. HSQC spectrum of **8** in DMSO-*d_6_* (500 MHz, 125 MHz).

**Figure S51.** HMBC spectrum of **8** in DMSO-*d_6_* (500 MHz, 125 MHz).

**Figure S52.** ^1^H-^1^H COSY spectrum of **8** in DMSO-*d_6_* (500 MHz, 500 MHz).

**Figure S53.** ^1^H-^1^H NOESY spectrum of **8** in DMSO-*d_6_* (500 MHz, 500 MHz)

Figure S54. ^1^H NMR spectrum of 9 in CD_3_OD-*d*_4_ (500 MHz).

**Figure S55.** ^13^C NMR spectrum of **9** in CD_3_OD-*d*_4_ (150 MHz).

**Figure S56**. HSQC spectrum of **9** in CD_3_OD-*d*_4_ (500 MHz, 125 MHz).

**Figure S57.** HMBC spectrum of **9** in CD_3_OD-*d*_4_ (500 MHz, 125 MHz).

**Figure S58.** ^1^H-^1^H COSY spectrum of **9** in CD_3_OD-*d*_4_ (500 MHz, 500 MHz).

**Figure S59.** ^1^H-^1^H NOESY spectrum of **9** in CD_3_OD-*d*_4_ (500 MHz, 500 MHz)

**Figure S60.** HRESIMS spectrum of compound **9**.

**Figure S61.** IR spectrum of compound **9**.

**Figure S62.** ECD spectrum of compound **9**.

Figure S63. ^1^H NMR spectrum of 10 in CDCl_3_ (600 MHz).

**Figure S64.** ^13^C NMR spectrum of **10** in CDCl_3_ (150 MHz).

**Figure S65**. HSQC spectrum of **10** in CDCl_3_ (500 MHz, 125 MHz).

**Figure S66.** HMBC spectrum of **10** in CDCl_3_ (500 MHz, 125 MHz).

**Figure S67.** ^1^H-^1^H COSY spectrum of **10** in CDCl_3_ (500 MHz, 500 MHz).

**Figure S68.** ^1^H-^1^H NOESY spectrum of **10** in CDCl_3_ (500 MHz, 500 MHz)

**Figure S69.** HRESIMS spectrum of compound **10**.

**Figure S70.** IR spectrum of compound **10**.

**Figure S71.** ECD spectrum of compound **10**.

Figure S72. ^1^H NMR spectrum of 11 in DMSO-*d_6_* (400 MHz).

**Figure S73.** ^13^C NMR spectrum of **11** in DMSO-*d_6_* (150 MHz).

**Figure S74**. HSQC spectrum of **11** in DMSO-*d_6_* (600 MHz, 150 MHz).

**Figure S75.** HMBC spectrum of **11** in DMSO-*d_6_* (600 MHz, 150 MHz).

**Figure S76.** ^1^H-^1^H COSY spectrum of **11** in DMSO-*d_6_* (600 MHz, 600 MHz).

Figure S77. ^1^H NMR spectrum of 12 in DMSO-*d_6_* (400 MHz).

**Figure S78.** ^13^C NMR spectrum of **12** in DMSO-*d_6_* (150 MHz).

**Figure S79**. HSQC spectrum of **12** in DMSO-*d_6_* (500 MHz, 125 MHz).

**Figure S80.** HMBC spectrum of **12** in DMSO-*d_6_* (500 MHz, 125 MHz).

**Figure S81.** ^1^H-^1^H COSY spectrum of **12** in DMSO-*d_6_* (500 MHz, 500 MHz).

**Figure S82.** Raw images for pH stability tests of *Monascus* pigments.

**Table S1.** Epigenetic and global regulators in *Talaromyces purpureogenus* OUCMDZ-019

| **Protein** | **Protein in**  **OUCMDZ-019** | **Size (aa)** | **Function** | **Mutant** | **Differences from WT** |
| --- | --- | --- | --- | --- | --- |
| Ash2 | 019026-PA | 665 | histone H3K4 methyltransferase  complex subunit | *ΔAsh2* | Significantly activated SMs and impaired conidiation |
| HdaA | 0195375-PA | 791 | Histone deacetylase | *ΔHdaA* | No significant difference |
| HosB | 019407-PA | 1165 | Histone deacetylase | *ΔHosB* | No significant difference |
| HosA | 0196689-PA | 486 | Histone deacetylase | *ΔHosA* | No significant difference |
| LaeA | 0198155-PA | 373 | Histone methyltransferase | *ΔLaeA* | Significant downregulation of SMs and fluffy spores |

Colony phenotype changes of *Talaromyces purpurogenus* OUCMDZ-019 after genetic manipulation of epigenetic regulators (representative plate images).

**Table S2.** *Tal*-*MrPig* cluster: protein identity and RNA-seq Log_2_FC (*ΔAsh*2/WT).

| ***MrPig*** | **Function** | ***Tal* in**  **OUCMDZ-019** | **Identity (%)** | ***ΔAsh2*/WT**  **_Log_2_FC** |
| --- | --- | --- | --- | --- |
| MrPigA | nrPKS | TalA | 57 | 10.39 |
| MrPigB | Transcription factor | TalB | 42 | 9.36 |
| MrPigC | C-11-ketoreductase | TalC | 71 | 9.95 |
| MrPigD | 4-O-Acylttansferase | TalD | 63 | 10.34 |
| MrPigE | NAD(P)H-dependent oxidoreductase | TalE | 69 | 6.70 |
| MrPigF | FAD-dependent oxidoreductase | — | — | — |
| MrPigG | Serine hydrolase | TalG | 70 | 7.38 |
| MrPigH | Enoyl reductase | TalH | 62 | 5.07 |
| MrPigI | Transcription factor | TalI | 41 | 1.43 |
| MrPigJ | FAS subunit alpha | TalG | 61 | 7.09 |
| MrPigK | FAS subunit beta | TalK | 57 | 7.09 |
| MrPigL | Ankyrin repeat protein | — | — | — |
| MrPigM | O-Acetyltransferase | TalM | 52 | 5.85 |
| MrPigN | FAD-dependent monoxygenase | TalN | 63 | 8.20 |
| MrPigO | Deacetylase | TalO | 43 | 1.27 |
| MrPigP | MFS multidrug transporter | TalP | 61 | 9.14 |
| — | P450 | TalQ | — | 10.69 |
| — | MFS multidrug transporter | TalR | — | 1.51 |
| — | FAD-linked oxidoreductase | TalS | — | 6.88 |

**Table S3.** ^1^H (500 MHz) and ^13^C (125 MHz) NMR data for compound **1** in DMSO-*d*_6_.

| No. | **1** | |
| --- | --- | --- |
|  | *δ*_C_, type | *δ*_H_, mult (*J* in H_Z_) |
| 1 | 141.9, CH | 8.20, s |
| 3 | 146.1, C |  |
| 4 | 118.9, CH | 7.08, s |
| 4a | 149.1, C |  |
| 5 | 97.5, CH | 6.69, s |
| 5a | 172.8, C |  |
| 6 | 104.5, C |  |
| 7 | 170.2, C |  |
| 8a | 85.2, C |  |
| 9 | 193.6, C |  |
| 9a | 116.8, C |  |
| 10 | 133.7, CH | 6.58, d (11.8) |
| 11 | 128.5, CH | 6.10, d (11.9) |
| 12 | 165.7, C |  |
| 13 | 194.9, C |  |
| 14 | 39.8, CH_2_ | 2.74, td (7.5, 2.5) |
| 15 | 24.0, CH_2_ | 1.49, m |
| 16 | 28.8, CH_2_ | 1.25, m |
| 17 | 28.6, CH_2_ | 1.25, m |
| 18 | 31.2, CH_2_ | 1.25, m |
| 19 | 22.1, CH_2_ | 1.25, m |
| 20 | 14.0, CH_3_ | 0.85, t (7.0) |
| 21 | 29.7, CH_3_ | 1.62, s |
| 1′ | 143.6, C |  |
| 2′ | 126.8, CH | 7.61, d (8.3) |
| 3′ | 130.6, CH | 8.06, d (8.3) |
| 4′ | 131.9, C |  |
| 5′ | 130.6, CH | 8.06, d (8.3) |
| 6′ | 126.8, CH | 7.61, d (8.3) |
| 7′ | 166.3, C |  |

**Table S4.** ^1^H (400 MHz) and ^13^C (150 MHz) NMR data for compound **2** in DMSO-*d*_6_.

| No. | **2** | |
| --- | --- | --- |
|  | *δ*_C_, type | *δ*_H_, mult (*J* in H_Z_) |
| 1 | 143.1, CH | 8.32, s |
| 3 | 147.8, C |  |
| 4 | 119.3, CH | 6.95, s |
| 4a | 150.1, C |  |
| 5 | 96.3, CH | 6.57, s |
| 5a | 172.3, C |  |
| 6 | 102.3, C |  |
| 7 | 170.4, C |  |
| 8a | 84.9, C |  |
| 9 | 194.6, C |  |
| 9a | 116.8, C |  |
| 10 | 133.1, CH | 7.09, d (12.1) |
| 11 | 129.4, CH | 6.38, d (11.9) |
| 12 | 165.6, C |  |
| 13 | 194.6, C |  |
| 14 | 39.5, CH_2_ | 2.70, t (7.3) |
| 15 | 24.2, CH_2_ | 1.47, m |
| 16 | 28.9, CH_2_ | 1.24, m |
| 17 | 28.6, CH_2_ | 1.24, m |
| 18 | 31.2, CH_2_ | 1.24, m |
| 19 | 22.1, CH_2_ | 1.25, m |
| 20 | 14.0, CH_3_ | 0.85, t (7.0) |
| 21 | 30.0, CH_3_ | 1.56, s |
| 1′ | 56.7, CH_2_ | 4.09, m |
| 2′ | 59.4, CH_2_ | 3.65, dd (6.8, 4.4) |

**Table S5.** ^1^H (400 MHz) and ^13^C (125 MHz) NMR data for compound **3** in DMSO-*d*_6_.

| No. | **3** | |
| --- | --- | --- |
|  | *δ*_C_, type | *δ*_H_, mult (*J* in H_Z_) |
| 1 | 142.0, CH | 8.45, s |
| 3 | 147.8, C |  |
| 4 | 118.4, CH | 6.90, s |
| 4a | 149.4, C |  |
| 5 | 96.7, CH | 6.59, s |
| 5a | 172.7, C |  |
| 6 | 103.3, C |  |
| 7 | 170.3, C |  |
| 8a | 85.0, C |  |
| 9 | 194.3, C |  |
| 9a | 116.4, C |  |
| 10 | 132.7, CH | 6.98, d (11.8) |
| 11 | 130.1, CH | 6.37, d (11.8) |
| 12 | 165.5, C |  |
| 13 | 194.7, C |  |
| 14 | 39.8, CH_2_ | 2.72, t (7.3) |
| 15 | 24.1, CH_2_ | 1.48, m |
| 16 | 28.9, CH_2_ | 1.24, m |
| 17 | 28.6, CH_2_ | 1.24, m |
| 18 | 31.2, CH_2_ | 1.24, m |
| 19 | 22.1, CH_2_ | 1.25, m |
| 20 | 14.0, CH_3_ | 0.85, t (7.0) |
| 21 | 30.0, CH_3_ | 1.57, s |
| 1′ | 168.1, C |  |
| 2′ | 65.7, CH_2_ | 5.00, m |
| 3′ | 61.2, CH_2_ | 3.93, dd (12.1, 3.0) |
|  |  | 4.16, dd (12.2, 5.5) |

**Table S6.** ^1^H (400 MHz) and ^13^C (100 MHz) NMR data for compound **4** in CDCl_3_.

| No. | **4** | |
| --- | --- | --- |
|  | *δ*_C_, type | *δ*_H_, mult (*J* in H_Z_) |
| 1 | 152.9, CH | 7.84, s |
| 3 | 153.2, C |  |
| 4 | 116.2, CH | 6.84, s |
| 4a | 140.0, C |  |
| 5 | 106.3, CH | 6.96, s |
| 5a | 171.2, C |  |
| 6 | 115.0, C |  |
| 7 | 169.1, C |  |
| 8a | 86.1, C |  |
| 9 | 190.5, C |  |
| 9a | 116.2, C |  |
| 10 | 129.3, CH | 6.35, d (12.9) |
| 11 | 125.8, CH | 6.23, d (12.8) |
| 12 | 167.9, C |  |
| 13 | 197.6, C |  |
| 14 | 41.9, CH_2_ | 2.94, q (7.1) |
| 15 | 23.8, CH_2_ | 1.60, m |
| 16 | 29.3, CH_2_ | 1.30, m |
| 17 | 29.3, CH_2_ | 1.26, m |
| 18 | 31.8, CH_2_ | 1.25, m |
| 19 | 22.8, CH_2_ | 1.27, m |
| 20 | 14.2, CH_3_ | 0.86, t (6.9) |
| 21 | 28.1, CH_3_ | 1.71, s |

**Table S7.** ^1^H (500 MHz) and ^13^C (125 MHz) NMR data for compound **5** in CDCl_3_.

| No. | **5** | |
| --- | --- | --- |
|  | *δ*_C_, type | *δ*_H_, mult (*J* in H_Z_) |
| 1 | 152.6, CH | 7.86, s |
| 3 | 153.2, C |  |
| 4 | 117.4, CH | 6.53, s |
| 4a | 138.9, C |  |
| 5 | 107.5, CH | 7.01, s |
| 5a | 170.5, C |  |
| 6 | 115.9, C |  |
| 7 | 168.8, C |  |
| 8a | 86.1, C |  |
| 9 | 190.1, C |  |
| 9a | 116.3, C |  |
| 10 | 135.1, CH | 7.21, d (15.6) |
| 11 | 123.7, CH | 6.52, d (15.5) |
| 12 |  |  |
| 13 | 197.5, C |  |
| 14 | 42.0, CH_2_ | 2.94, q (7.1) |
| 15 | 23.7, CH_2_ | 1.60, m |
| 16 | 29.3, CH_2_ | 1.30, m |
| 17 | 29.3, CH_2_ | 1.26, m |
| 18 | 31.8, CH_2_ | 1.25, m |
| 19 | 22.8, CH_2_ | 1.27, m |
| 20 | 14.2, CH_3_ | 0.87, t (7.1) |
| 21 | 27.9, CH_3_ | 1.72, s |

**Table S8.** ^1^H (400 MHz) and ^13^C (125 MHz) NMR data for compound **6** in DMSO-*d*_6_.

| No. | **6** | |
| --- | --- | --- |
|  | *δ*_C_, type | *δ*_H_, mult (*J* in H_Z_) |
| 1 | 147.7, CH | 7.48, s |
| 3 | 160.6, C |  |
| 4 | 107.9, CH | 6.32, s |
| 4a | 144.7, C |  |
| 5 | 104.6, CH | 5.29, s |
| 6 | 190.6, C |  |
| 6a | 82.6, C |  |
| 8 | 170.0, C |  |
| 9 | 55.9, CH | 4.34, d (12.2) |
| 9a | 42.5, CH | 3.84, d (12.2) |
| 9b | 114.2, C |  |
| 10 | 42.5, CH_2_ | 2.45, m |
| 11 | 64.1, CH | 3.90, m |
| 12 | 23.5, C | 1.10, d (6.2) |
| 13 | 22.7, CH_3_ | 1.42, s |
| 14 | 202.8, C |  |
| 15 | 41.8, CH_2_ | 2.57, m; 2.88, m |
| 16 | 22.4, CH_2_ | 1.47, m |
| 17 | 28.5, CH_2_ | 1.26, m |
| 18 | 28.3, CH_2_ | 1.21, m |
| 19 | 31.1, CH_3_ | 1.21, m |
| 20 | 22.1, CH_3_ | 1.23, m |
| 21 | 14.0, C | 0.85, t (7.0) |

**Table S9.** ^1^H (400 MHz) and ^13^C (150 MHz) NMR data for compound **7** in DMSO-*d*_6_.

| No. | **7** | |
| --- | --- | --- |
|  | *δ*_C_, type | *δ*_H_, mult (*J* in H_Z_) |
| 1 | 63.3, CH_2_ | 4.80, dd (13.0) |
|  |  | 4.91, dd (12.3) |
| 3 | 155.6, C |  |
| 4 | 111.5, CH | 6.12, s |
| 4a | 149.3, C |  |
| 5 | 28.7, CH_2_ | 2.57, m; 2.64, m |
| 5a | 43.2, CH | 3.07, ddd (4.9, 10.6, 13.0) |
| 6 | 54.2, CH | 4.46, d (13.1) |
| 7 | 170.7, C |  |
| 8a | 83.2, C |  |
| 9 | 190.5, C |  |
| 9a | 117.2, C |  |
| 10 | 135.9, CH | 7.08, d (15.5) |
| 11 | 123.0, CH | 6.22, d (15.5) |
| 12 | 166.7, C |  |
| 13 | 203.5, C |  |
| 14 | 42.2, CH_2_ | 2.82, td (14.6, 7.2) |
|  |  | 2.65, m |
| 15 | 22.5, CH_2_ | 1.51, m |
| 16 | 28.4, CH_2_ | 1.25, m |
| 17 | 28.4, CH_2_ | 1.25, m |
| 18 | 31.1, CH_2_ | 1.25, m |
| 19 | 22.0, CH_2_ | 1.25, m |
| 20 | 13.9, CH_3_ | 0.86, t (7.0) |
| 21 | 17.2, CH_3_ | 1.40, s |

**Table S10.** ^1^H (500 MHz) and ^13^C (150 MHz) NMR data for compound **8** in DMSO-*d*_6_.

| No. | **8** | |
| --- | --- | --- |
|  | *δ*_C_, type | *δ*_H_,mult (*J* in H_Z_) |
| 1 | 63.3, CH_2_ | 4.80, d (13.1) |
|  |  | 4.89, d (13.1) |
| 3 | 155.5, C |  |
| 4 | 111.6, CH | 6.15, s |
| 4a | 149.5, C |  |
| 5 | 28.7, CH_2_ | 2.57, m; 2.64, m |
| 5a | 42.6, CH | 2.79, ddd |
|  |  | (3.4, 8.9, 10.5) |
| 6 | 47.5, CH | 3.03, dd (2.2, 10.4) |
| 7 | 173.8, C |  |
| 8a | 82.2, C |  |
| 9 | 191.5, C |  |
| 9a | 117.6, C |  |
| 10 | 135.9, CH | 7.10, d (15.5) |
| 11 | 122.8, CH | 6.21, d (15.5) |
| 12 | 166.7, C |  |
| 13 | 67.1, CH | 3.70, d (8.4) |
| 14 | 33.2, CH_2_ | 1.73, m; 1.43, m |
| 15 | 25.9, CH_2_ | 1.43, m |
| 16 | 28.9, CH_2_ | 1.25, m |
| 17 | 28.7, CH_2_ | 1.25, m |
| 18 | 31.3, CH_2_ | 1.25, m |
| 19 | 22.1, CH_2_ | 1.25, m |
| 20 | 14.0, CH_3_ | 0.86, t (5.6) |
| 21 | 16.8, CH_3_ | 1.33, s |

**Table S11.** ^1^H (500 MHz) and ^13^C (150 MHz) NMR data for compound **9** in MeOH-*d*_4_.

| No. | **9** | |
| --- | --- | --- |
|  | *δ*_C_, type | *δ*_H_, mult (*J* in H_Z_) |
| 1 | 64.9, CH_2_ | 4.85, m |
|  |  | 4.96, dd (13.1, 1.9) |
| 3 | 157.9, C |  |
| 4 | 111.8, CH | 5.83, s |
| 4a | 151.0, C |  |
| 5 | 33.3, CH_2_ | 2.27, m；2.57, m |
| 6 | 40.8, CH | 2.61, m |
| 7 | 76.0, C |  |
| 8 | 200.3, C |  |
| 8a | 118.6, C |  |
| 9 | 137.6, CH | 7.07, d (15.5) |
| 10 | 123.9, CH | 6.33, d (15.5) |
| 11 | 169.5, C |  |
| 12 | 42.8, CH_2_ | 2.50, m |
|  |  | 2.92, dd (16.9,2.75) |
| 13 | 212.6, C |  |
| 14 | 44.0, CH_2_ | 2.50, m |
| 15 | 24.9, CH_2_ | 1.57, m |
| 16 | 30.2, CH_2_ | 1.30, m |
| 17 | 30.2, CH_2_ | 1.30, m |
| 18 | 32.9, CH_2_ | 1.29, m |
| 19 | 23.7, CH_2_ | 1.30, m |
| 20 | 14.4, CH_2_ | 0.90, t (7.1) |
| 21 | 19.3, CH_2_ | 1.17，s |

**Table S12.** ^1^H (600 MHz) and ^13^C (150 MHz) NMR data for compound **10** in CDCl_3_.

| No. | **10** | |
| --- | --- | --- |
|  | *δ*_C_, type | *δ*_H_,mult (*J* in H_Z_) |
| 1 | 193.6, C |  |
| 2 | 132.4, C |  |
| 3 | 147.8, C |  |
| 4 | 33.3, CH_2_ | 2.51, m |
| 4a | 42.5, CH | 3.16, ddd |
|  |  | (13.1, 10.2, 5.34) |
| 5 | 55.0, CH | 3.67, d (13.3) |
| 6 | 169.7, C |  |
| 7a | 83.3, C |  |
| 8 | 12.0, CH_3_ | 1.81, s |
| 9 | 48.6, CH_2_ | 3.34, d (16.6) |
| 10 | 204.0, C |  |
| 11 | 37.3, CH_2_ | 2.76, m |
| 12 | 27.7, CH_2_ | 2.68, m |
| 13 | 177.2, C |  |
| 14 | 202.7, C |  |
| 15 | 43.1, CH_2_ | 2.59, m; 3.00, m |
| 16 | 23.2, CH_2_ | 1.59, m |
| 17 | 29.1, CH_2_ | 1.28, m |
| 18 | 29.2, CH_2_ | 1.25, m |
| 19 | 31.8, CH_2_ | 1.25, m |
| 20 | 22.7, CH_2_ | 1.27, m |
| 21 | 14.2, CH_3_ | 0.87, t (7.1) |
| 22 | 17.3, CH_3_ | 1.47, s |

**Table S13**. ^1^H (400 MHz) and ^13^C (150 MHz) NMR data for compound **11** in DMSO-*d*_6_.

| No. | **11** | |
| --- | --- | --- |
|  | *δ*_C_, type | *δ*_H_, mult (*J* in H_Z_) |
| 1 | 64.3, CH_2_ | 3.84, dd (12.7, 6.4) |
| 3 | 164.2, C |  |
| 4 | 103.2, CH | 5.34, s |
| 4a | 148.2, C |  |
| 5 | 34.0, CH_2_ | 2.25, m |
|  |  | 2.58, dd (18.1, 4.9) |
| 6 | 71.9, CH | 3.75, dd (8.7, 4.9) |
| 7 | 75.6, C |  |
| 8 | 196.8, C |  |
| 8a | 112.6, C |  |
| 9 | 43.5, CH_2_ | 2.17, m |
|  |  | 2.27, m |
| 10 | 63.6, CH | 4.72, m |
| 11 | 18.3, CH_3_ | 1.09, d (4.9) |
| 12 | 23.4, CH_3_ | 1.07, s |

**Table S14.** ^1^H (400 MHz) and ^13^C (150 MHz) NMR data for compound **12** in DMSO-*d*_6_.

| No. | **12** | |
| --- | --- | --- |
|  | *δ*_C_, type | *δ*_H_, mult (*J* in H_Z_) |
| 1 | 64.4, CH_2_ | 4.74, dd (22.3, 12.6) |
| 3 | 158.3, C |  |
| 4 | 104.1, CH | 5.47, s |
| 4a | 148.2, C |  |
| 5 | 33.9, CH_2_ | 2.63, m (17.9, 5.0) |
|  |  | 2.29, dd (18.2, 8.3) |
| 6 | 71.9, CH | 3.76, dd (8.1, 4.8) |
| 7 | 75.6, C |  |
| 8 | 196.7, C |  |
| 8a | 114.2, C |  |
| 9 | 124.9, CH | 6.04, d (15.5) |
| 10 | 132.9, CH | 4.72, dt (22.3,7.0) |
| 11 | 18.1, CH_3_ | 1.81, d (6.8) |
| 12 | 18.6, CH_3_ | 1.10, s |

**Table S15.** Culture media compositions used in this study.

| Culture media | Composition (per 1L) |
| --- | --- |
| PDB medium | Potato infusion from 200 g potatoes, and 20 g dextrose; sterilized at 121 °C for 15–20 min. |
| PDB medium (pH 2.2) | PDB medium was prepared, and the pH was adjusted to ~2.2 by dropwise addition of concentrated HCl before sterilization. The medium was then sterilized at 121 °C for 15–20 min and cooled to room temperature before use. |
| PDA medium | Potato infusion from 200 g potatoes, 20 g dextrose, and 20 g agar; sterilized at 121 °C for 15–20 min. |
| High-osmotic PDB | 1 mol/L Sorbitol, PDB medium |
| High-osmotic PDA medium (500 μg/mL G418) | Potato infusion from 200 g potatoes, 20 g dextrose, and 20 g agar, supplemented with an osmotic stabilizer (1 mol/L sorbitol) to 1 L; the medium was sterilized at 121 °C for 15–20 min. After cooling to ~50 °C, G418 was added to a final concentration of 500 μg/mL, mixed thoroughly, and poured into plates for selection. |
| LB medium | Tryptone 10 g, yeast extract 5 g, and NaCl 10 g; sterilized at 121 °C for 15–20 min. |
| LB medium (amp-resistance) | Tryptone 10 g, yeast extract 5 g, NaCl 10 g, and agar 20 g; sterilized at 121 °C for 15–20 min. After sterilization and cooling to approximately 50 °C, add sodium ampicillin to a final concentration of 100 μg/mL. The medium was mixed thoroughly and poured into plates for selection. |
| YPD medium | Yeast extract 10 g, peptone 20 g, and dextrose (glucose) 20 g; sterilized at 121 °C for 15–20 min. |
| SDYS medium | Peptone 10 g, glucose 40 g, yeast extract 10 g, NaCl 1 g, KH₂PO₄ 0.3 g, and MgSO₄ 0.1 g. All components were fully dissolved in tap water and the volume was adjusted to 1 L. The medium was sterilized by autoclaving at 121 °C for 15–20 min and cooled to room temperature before use. |

**Table S16.** Solution compositions used in this study.

| Solution | Composition |
| --- | --- |
| TF_0_ | Maleic acid (1.16 g) was dissolved in 100 mL of purified water, and the pH was adjusted to 7.5 with NaOH. The solution was then brought to a final volume of 200 mL with ddH₂O. |
| TF_1_ | (NH_4_)_2_SO_4_ (1.58 g) was fully dissolved in an appropriate volume of TF_0_ solution, and the volume was adjusted to 20 mL with TF_0_. |
| TF_2_ | Yatalase (0.1 g) was fully dissolved in 10 mL of TF_1_ solution. The solution was sterilized by filtration through a 0.22 μm membrane filter and stored at 4 °C. |
| 1 M D-sorbitol | 1 mol/L D-sorbitol solution was prepared by dissolving D-sorbitol (36.44 g) in an appropriate volume of ddH₂O and adjusting the final volume to 200 mL. |
| STC | 1 mol/L D-Glucitol, 10 mmol/L Tris, 50 mmol/L CaCl_2_, pH 7.5 |
| PEG-mediated transformation solution  (PTC) | 40% PEG 6000, 50 mmol/L Tris, 50 mmol/L CaCl_2_·2H_2_O, pH 7.5 |

**Table S17.** Primer information.

| **Primer** | **Sequence (5’→3’)** | **Description** |
| --- | --- | --- |
| 026-1-F | CCCGGGGATCCTCTAGAGATAGACTCACGACAGACTCCGCATTGG | Construction of pMD19T-*Ash2*-KO plasmid |
| 026-1-R | ATAACTTGCACAAATGGGAGGGCTTATATTCAGAAGTAGTGGAGT |  |
| 026-G418-F | ACTACTTCTGAATATAAGCCCTCCCATTTGTGCAAGTTATTCGGC |  |
| 026-G418-R | CTATGAGTCGTTACCCAGAATTCTATCATTTTGTCATCTAAGTAT |  |
| 026-2-F | TAGATGACAAAATGATAGAATTCTGGGTAACGACTCATAGGAGAG |  |
| 026-2-R | CATGCCTGCAGGTCGACGATTGATGGACGAGATCCTACTAATCTA |  |
| 026-1+G418-F | AGACTCACGACAGACTCCGCATTGGGGCTG | Construction of *ΔAsh2* deletion cassette |
| 026-1+G418-R | AGAAGAACTCGTCAAGAAGGCGATAGAAGG |  |
| G418+026-2-F | GCGCGTTGGATTAGAGGTCGACAGAAGATG |  |
| G418+026-2-R | TGATGGACGAGATCCTACTAATCTATCCAC |  |
| 026-yz-F | GCTCCAAACGAACCAAAAAAGAC | *ΔAsh2* diagnostic PCR |
| 026-yz-R | CAGGCGGCAAGAAGGCAAACAAT |  |
| wai+026-1+G418-F | CAGGCTTGCTGGTGGTAGGCTTT |  |
| wai+026-1+G418-R | CCGTCCGCTTGCTCATTACATTT |  |
| 8155-1-F | CCCGGGGATCCTCTAGAGATGCACATCCACGTAACATGGAGTCCA | Construction of pMD19T-*LaeA*-KO plasmid |
| 8155-1-R | ATAACTTGCACAAATGGGAGTTTGTGTGTGCGAGAGAGAGTGTGG |  |
| 8155-G-418-F | CTCTCTCTCGCACACACAAACTCCCATTTGTGCAAGTTATTCGGC |  |
| 8155-G-418-R | ATATTAGGTATATATAAAGCTTCTGGGTAACGACTCATAGGAGAG |  |
| 8155-2-F | CTATGAGTCGTTACCCAGAAGCTTTATATATACCTAATATTTTCC |  |
| 8155-2-R | CATGCCTGCAGGTCGACGATGTACATCAACAACGACTAGACCAGG |  |
| 8155-1+G418-F | GCACATCCACGTAACATGGAGTCCATCGAG | Construction of *ΔLaeA* deletion cassette |
| 8155-1+G418-R | AGAAGAACTCGTCAAGAAGGCGATAGAAGG |  |
| G418+8155-2-F | GCGCGTTGGATTAGAGGTCGACAGAAGATG |  |
| G418+8155-2-R | GTACATCAACAACGACTAGACCAGGAAGAT |  |
| 8155yz-F | TATGTTGTCCCCTGTCTCTTGCC | *ΔLaeA* diagnostic PCR |
| 8155yz-R | ACCATCTCCCCACTTTCTTCTCG |  |
| wai+8155-1+G418-F | TGTGGTTGTGGTTGGTGTTGTTAT |  |
| wai+8155-1+G418-R | CATTGATGGTCTGCTGCTGATTTT |  |
| 5375-1-F | GGATCCTCTAGAGATGTGTCGTCGACCTGATTGGTATGTC | Construction of pMD19T-*HdaA*-KO plasmid |
| 5375-1-R | TTCTCCTTGCGCAACACAGCGGGTGGTTTGTTTGGCCGTCG |  |
| 5375-G-418-F | GCTGTGTTGCGCAAGGAGAAGAATTCATGC |  |
| 5375-G-418-R | TCTAGAAAGAAGGATTACCTCTAAA |  |
| 5375-2-F | AGGTAATCCTTCTTTCTAGACGCGAAGTCGTGATGACTGA |  |
| 5375-2-R | CTGCAGGTCGACGATGATGATGTCTTTCCGTCCACCTCAG |  |
| 5375-1+5375-yz-F | TCTGCCCTATTTTGCTATCACTCG | *ΔHdaA* diagnostic PCR |
| 5375-1+5375-yz-R | CAATCGCATTCTTGACCTTTCG |  |
| 5375-yz-F | AGACATCCCCAATGCTACTACA |  |
| 5375-yz-R | ACAATCAGAATCTTGCGACACG |  |
| 5375+5375-2-yz-F | AGTCTGTGGATCTATCGTGCTT |  |
| 5375+5375-2-yz-R | GAGTCGGAGTGTAGTGTTCGGT |  |
| 407-1-F | GGATCCTCTAGAGATGGCTAGGCGCTGTTACTGTTAACAG | Construction of pMD19T-*HosB*-KO plasmid |
| 407-1-R | GCTCACATGGCACCACTCATAAATTGATCAACAGTGAA |  |
| 407-G-418-F | ATGAGTGGTGCCATGTGAGCCTCCCATTTGTGCAAGTTAT |  |
| 407-G-418-R | TTCTGGGTAACGACTCATAGGAGAGTTGTAAAAAAGTTTC |  |
| 407-2-F | CTATGAGTCGTTACCCAGAAACAGTCCATTGAGCATCTTC |  |
| 407-2-R | CTGCAGGTCGACGATGATGAGCAACTTGCACTGCG |  |
| 407-1+G-418-F | GGCTAGGCGCTGTTACTGTTAACAGCCTGA | Construction of *ΔHosB* deletion cassette |
| 407-1+G-418-R | ATGTCCTGATAGCGGTCCGCCACACCCAGC |  |
| G-418+407-2-F | TGCTGAAACCTCCGTATGCCTGAAGCCGAC |  |
| G-418+407-2-R | GATGAGCAACTTGCACTGCGGGTGCGAGCA |  |
| wai+407-1+G-418-F | AAGAGGCCGGTGGCTCGATATT | *ΔHosB* diagnostic PCR |
| wai+407-1+G-418-R | CTCCGTCCGCTTGCTCATTACA |  |
| 407-1+407-yz-F | CCTGCGTGTCTCAAATAACCCC |  |
| 407-1+407-yz-R | GTGCGTAATCCCAACATGAACG |  |
| 407-yz-F | CCTGTGAGAACGGAGAAATGGAT |  |
| 407-yz-R | GCTGAGGTTTCGTAGATTGTGGC |  |
| 407+407-2-yz-F | AGCCACAATCTACGAAACCTCAG |  |
| 407+407-2-yz-R | GAGCGAACGAGTAAATAGTCAAGC |  |
| 6689-1-F | CCCGGGGATCCTCTAGAGATATCACAGCCCTGGCAATCCTGGCCA | Construction of pMD19T-*HosA*-KO plasmid |
| 6689-1-R | ATAACTTGCACAAATGGGAGTTGCCGAGAGTAGTAAGTGCGGTCA |  |
| 6689-G-418-F | GCACTTACTACTCTCGGCAACTCCCATTTGTGCAAGTTATTCGGC |  |
| 6689-G-418-R | CCAAACAATATCGAATAGCGTTCTGGGTAACGACTCATAGGAGAG |  |
| 6689-2-F | CTATGAGTCGTTACCCAGAACGCTATTCGATATTGTTTGGCGTAT |  |
| 6689-2-R | CATGCCTGCAGGTCGACGATGCACGTACAACGCCATTGCGGGGAC |  |
| 6689-1+G-418-F | ATCACAGCCCTGGCAATCCTGGCCACCAGG | Construction of *ΔHosA* deletion cassette |
| 6689-1+G-418-R | AGAAGAACTCGTCAAGAAGGCGATAGAAGG |  |
| G-418+6689-2-F | AATGAGCAAGCGGACGGAGTGCTGAAACCT |  |
| G-418+6689-2-R | GCACGTACAACGCCATTGCGGGGACGATCA |  |
| wai+6689-1+G-418-F | CCAGCACTGCAAACTACCAAG | *ΔHosA* diagnostic PCR |
| wai+6689-1+G-418-R | CCATCCCCTAAGACAAAATCC |  |
| 6689-1+6689-yz-F | TTACTAACCACCACATCACCAAGC |  |
| 6689-1+6689-yz-R | GCACGAGCCGATTATGTCTTTG |  |
| 6689-yz-F | GGACAATCGCATCCAATGAAAC |  |
| 6689-yz-R | AGCAGTGGTAAGCCCAAAGTCT |  |
| 6689+6689-2-yz-F | CGCAAACTCAACAATAAACAAG |  |
| 6689+6689-2-yz-R | AACCATCCACAAAACAACAATC |  |
| M13-F | TGTAAAACGACGGCCAGT | Plasmid diagnostic PCR |
| M13-R | CAGGAAACAGCTATGACC |  |

**Table S18.** Raw data for color value determination.

| **Compunds No.** | **λ_max_ (nm)** | ***f*** | ***A***  **(rep 1)** | ***A***  **(rep 2)** | ***A***  **(rep 3)** | ***m* (g)** | ***E* (U/g)** |
| --- | --- | --- | --- | --- | --- | --- | --- |
| **1** | 517 | 80 | 0.43 | 0.42 | 0.43 | 0.0000125 | 27520 |
| **2** | 510 | 80 | 0.31 | 0.31 | 0.31 | 0.0000125 | 19840 |
| **3** | 510 | 80 | 0.33 | 0.32 | 0.32 | 0.0000125 | 21120 |
| **4** | 410 | 40 | 0.3 | 0.3 | 0.3 | 0.000025 | 4800 |
| **5** | 410 | 40 | 0.3 | 0.3 | 0.3 | 0.000025 | 4800 |
| **7** | 388 | 40 | 0.58 | 0.58 | 0.58 | 0.000025 | 9280 |
| **9** | 386 | 40 | 0.32 | 0.32 | 0.32 | 0.000025 | 5120 |

**Table S19.** Raw data for lipid-lowering activity assay (OD at 510 nm).

| **Absorbance (OD_510_)** | | | |
| --- | --- | --- | --- |
| **Group** | **Replicate 1** | **Replicate 2** | **Replicate 3** |
| Blank | 0.091 | 0.088 | 0.086 |
| OA + PA (Model) | 0.175 | 0.160 | 0.152 |
| sim | 0.122 | 0.118 | 0.104 |
| ros | 0.118 | 0.132 | 0.129 |
| Compound **1** | 0.134 | 0.147 | 0.137 |
| Compound **2** | 0.135 | 0.151 | 0.142 |
| Compound **3** | 0.129 | 0.124 | 0.124 |
| Compound **7** | 0.148 | 0.161 | 0.150 |
| Compound **9** | 0.140 | 0.151 | 0.167 |

| **Inhibition rate (%)** | | | |
| --- | --- | --- | --- |
| **Group** | **Replicate 1** | **Replicate 2** | **Replicate 3** |
| sim | 54.50 | 59.91 | 78.83 |
| ros | 59.91 | 40.99 | 45.05 |
| Compound **1** | 38.29 | 20.72 | 34.23 |
| Compound **2** | 36.94 | 15.32 | 27.48 |
| Compound **3** | 45.05 | 51.80 | 51.80 |
| Compound **7** | 19.37 | 1.80 | 16.67 |
| Compound **9** | 30.18 | 15.32 | -6.31 |

**Table S20.** DFT-optimized structures for low-energy conformers of **1**.

| **Conformers** | **Conf. A** | **Conf. B** | **Conf. C** |
| --- | --- | --- | --- |
| DFT-optimized structures |  |  |  |
| Population | 28.94% | 6.09% | 6.09% |
| **Conformers** | **Conf. D** | **Conf. E** |  |
| DFT-optimized structures |  |  |  |
| Population | 10.85% | 48.02% |  |

**Table S21.** DFT-optimized structures for low-energy conformers of **2**.

| **Conformers** | **Conf. A** | **Conf. B** | **Conf. C** |
| --- | --- | --- | --- |
| DFT-optimized structures |  |  |  |
| Population | 5.32% | 1.03% | 1.96% |
| **Conformers** | **Conf. D** | **Conf. E** | **Conf. F** |
| DFT-optimized structures |  |  |  |
| Population | 0.92% | 45.53% | 1.43% |
| **Conformers** | **Conf. G** | **Conf. H** |  |
| DFT-optimized structures |  |  |  |
| Population | 0.55% | 43.26% |  |

**Table S22.** DFT-optimized structures for low-energy conformers of **9**.

| **Conformers** | **Conf. A** | **Conf. B** | **Conf. C** |
| --- | --- | --- | --- |
| DFT-optimized structures |  |  |  |
| Population | 1.62% | 1.95% | 32.60% |
| **Conformers** | **Conf. D** | **Conf. E** | **Conf. F** |
| DFT-optimized structures |  |  |  |
| Population | 1.62% | 8.72% | 2.04% |
| **Conformers** | **Conf. G** |  |  |
| DFT-optimized structures |  |  |  |
| Population | 51.46% |  |  |

**Table S23.** DFT-optimized structures for low-energy conformers of **10a**.

| **Conformers** | **Conf. A** | **Conf. B** | **Conf. C** |
| --- | --- | --- | --- |
| DFT-optimized structures |  |  |  |
| Population | 31.16% | 13.04% | 7.47% |
| **Conformers** | **Conf. D** | **Conf. E** | **Conf. F** |
| DFT-optimized structures |  |  |  |
| Population | 31.64% | 5.12% | 7.03% |
| **Conformers** | **Conf. G** | **Conf. H** |  |
| DFT-optimized structures |  |  |  |
| Population | 3.68% | 0.86% |  |

**Table S24.** DFT-optimized structures for low-energy conformers of **10b**.

| **Conformers** | **Conf. A** | **Conf. B** | **Conf. C** |
| --- | --- | --- | --- |
| DFT-optimized structures |  |  |  |
| Population | 34.20% | 9.61% | 1.99% |
| **Conformers** | **Conf. D** | **Conf. E** | **Conf. F** |
| DFT-optimized structures |  |  |  |
| Population | 5.19% | 16.35% | 14.89% |
| **Conformers** | **Conf. G** | **Conf. H** |  |
| DFT-optimized structures |  |  |  |
| Population | 14.50% | 3.28% |  |

**Table S25.** The Cartesian coordinates of the lowest energy conformers (Conf. A) for **1**.

| **Conf. A** | **X axis** | **Y axis** | **Z axis** | **Conf. A** | **X axis** | **Y axis** | **Z axis** |
| --- | --- | --- | --- | --- | --- | --- | --- |
| C | 2.103778 | 0.929808 | -1.01558 | C | -4.18729 | -2.09372 | 0.596322 |
| C | 1.631627 | 0.000216 | -2.11373 | C | -7.90682 | -2.51288 | 0.172199 |
| C | 0.489564 | -0.91485 | -1.67292 | O | -8.88569 | -1.68107 | -0.2406 |
| C | -0.49216 | -0.22239 | -0.8024 | O | -8.12007 | -3.66191 | 0.513385 |
| C | -0.12936 | 0.992964 | -0.10601 | C | 1.106573 | 0.807059 | -3.32973 |
| C | 1.157203 | 1.537859 | -0.18174 | H | 1.453681 | 2.336832 | 0.484772 |
| C | 3.494566 | 0.958945 | -1.07439 | H | -2.02261 | -1.67513 | -1.16066 |
| C | 3.919947 | -0.10321 | -1.99317 | H | -0.91838 | 2.398398 | 1.355444 |
| O | 2.795112 | -0.71705 | -2.52766 | H | -3.61452 | 0.681234 | 2.647547 |
| C | -1.73458 | -0.77269 | -0.63546 | H | -4.49538 | 2.887601 | 2.852046 |
| N | -2.68167 | -0.20633 | 0.162139 | H | -4.00899 | 5.564441 | 0.747363 |
| C | -2.37738 | 0.9523 | 0.879738 | H | 6.115001 | 2.715901 | -1.00877 |
| C | -1.14178 | 1.525344 | 0.753395 | H | 6.160998 | 0.960869 | -1.08325 |
| C | -3.35685 | 1.415324 | 1.885528 | H | 7.702859 | 1.961597 | 0.664349 |
| C | -3.88503 | 2.643468 | 1.988491 | H | 6.30734 | 2.680474 | 1.462822 |
| C | -3.76326 | 3.705217 | 0.957619 | H | 5.403177 | 0.406717 | 1.977644 |
| O | -4.07639 | 4.91212 | 1.471487 | H | 6.97663 | 0.703418 | 2.696127 |
| O | -3.46762 | 3.536125 | -0.21162 | H | 6.47851 | -0.94924 | 0.157553 |
| O | 5.021044 | -0.51678 | -2.30858 | H | 8.066645 | -0.59053 | 0.821926 |
| C | 4.353828 | 1.823391 | -0.26112 | H | 7.438826 | -1.76797 | 2.953249 |
| C | 5.867538 | 1.799648 | -0.45175 | H | 5.845902 | -2.11493 | 2.295278 |
| C | 6.636881 | 1.811537 | 0.88195 | H | 6.899467 | -3.45716 | 0.449716 |
| C | 6.467174 | 0.543 | 1.735148 | H | 8.49221 | -3.10976 | 1.104688 |
| C | 7.01134 | -0.74256 | 1.095602 | H | 6.279799 | -4.66797 | 2.581768 |
| C | 6.899647 | -1.96639 | 2.014602 | H | 7.718612 | -5.37916 | 1.832806 |
| C | 7.439708 | -3.25751 | 1.386109 | H | 7.884676 | -4.31821 | 3.241314 |
| C | 7.325154 | -4.47426 | 2.310447 | H | -4.92715 | 1.02479 | -0.58139 |
| O | 3.855091 | 2.602773 | 0.560281 | H | -7.21103 | 0.044305 | -0.58246 |
| O | 0.360641 | -2.06085 | -2.07082 | H | -5.62859 | -3.66524 | 0.901531 |
| C | -4.0108 | -0.77114 | 0.181959 | H | -3.3389 | -2.67794 | 0.937481 |
| C | -5.09099 | 0.006238 | -0.24573 | H | -9.7229 | -2.18183 | -0.20296 |
| C | -6.36759 | -0.54838 | -0.24872 | H | 0.223461 | 1.395645 | -3.06875 |
| C | -6.56034 | -1.87473 | 0.163134 | H | 0.853054 | 0.11354 | -4.1368 |
| C | -5.46574 | -2.64232 | 0.581247 | H | 1.895015 | 1.485009 | -3.66869 |

**Table S26.** The Cartesian coordinates of the lowest energy conformers (Conf. B) for **1**.

| **Conf. A** | **X axis** | **Y axis** | **Z axis** | **Conf. A** | **X axis** | **Y axis** | **Z axis** |
| --- | --- | --- | --- | --- | --- | --- | --- |
| C | -2.21976 | 0.846473 | 1.083999 | C | 4.18485 | -1.99757 | -0.40906 |
| C | -1.68645 | 0.031222 | 2.24333 | C | 7.962294 | -2.10695 | -0.0014 |
| C | -0.50794 | -0.86104 | 1.854444 | O | 8.087843 | -3.41415 | -0.31118 |
| C | 0.425235 | -0.1955 | 0.912608 | O | 8.914796 | -1.40318 | 0.28086 |
| C | -0.00694 | 0.941371 | 0.128588 | C | -1.18333 | 0.956234 | 3.381906 |
| C | -1.3167 | 1.429863 | 0.186434 | H | -1.66253 | 2.157112 | -0.5361 |
| C | -3.6091 | 0.813271 | 1.165603 | H | 2.029447 | -1.54138 | 1.355497 |
| C | -3.96858 | -0.19124 | 2.173005 | H | 0.689434 | 2.258021 | -1.45767 |
| O | -2.80716 | -0.70533 | 2.734241 | H | 3.431311 | 0.565863 | -2.66998 |
| C | 1.689805 | -0.69903 | 0.765358 | H | 4.216998 | 2.783721 | -3.06283 |
| N | 2.594974 | -0.15645 | -0.09491 | H | 3.664952 | 5.591427 | -1.15229 |
| C | 2.223071 | 0.923847 | -0.89765 | H | -6.2976 | 2.390175 | 1.088883 |
| C | 0.964252 | 1.448167 | -0.79187 | H | -6.27164 | 0.638924 | 1.171244 |
| C | 3.160868 | 1.346031 | -1.95989 | H | -7.89838 | 1.56931 | -0.53863 |
| C | 3.635928 | 2.58229 | -2.16861 | H | -6.55321 | 2.331594 | -1.38291 |
| C | 3.492494 | 3.71333 | -1.21739 | H | -5.58177 | 0.098416 | -1.90746 |
| O | 3.743831 | 4.889537 | -1.82724 | H | -7.18965 | 0.315252 | -2.57661 |
| O | 3.229492 | 3.621218 | -0.0318 | H | -6.52575 | -1.30273 | -0.05392 |
| O | -5.04341 | -0.63063 | 2.539516 | H | -8.15547 | -0.95973 | -0.61332 |
| C | -4.52225 | 1.564414 | 0.299538 | H | -7.56898 | -3.18326 | -1.34217 |
| C | -6.02897 | 1.483256 | 0.525428 | H | -7.67425 | -2.16061 | -2.7676 |
| C | -6.83403 | 1.457041 | -0.7854 | H | -5.17018 | -1.99604 | -2.83953 |
| C | -6.64099 | 0.187118 | -1.63212 | H | -5.0608 | -2.95334 | -1.37053 |
| C | -7.12095 | -1.1072 | -0.95668 | H | -6.24259 | -3.9415 | -4.04169 |
| C | -7.07641 | -2.34932 | -1.86291 | H | -4.65237 | -4.37681 | -3.39495 |
| C | -5.66638 | -2.79632 | -2.27495 | H | -6.12387 | -4.91099 | -2.5657 |
| C | -5.66942 | -4.07751 | -3.11593 | H | 4.78912 | 1.237649 | 0.501405 |
| O | -4.07471 | 2.295556 | -0.59245 | H | 7.125991 | 0.356471 | 0.531412 |
| O | -0.31714 | -1.96402 | 2.339626 | H | 5.681838 | -3.52319 | -0.62171 |
| C | 3.949819 | -0.6568 | -0.09703 | H | 3.360469 | -2.64688 | -0.68514 |
| C | 4.997423 | 0.204153 | 0.245276 | H | 9.038087 | -3.63172 | -0.25898 |
| C | 6.296953 | -0.28954 | 0.265095 | H | -0.33468 | 1.564336 | 3.058754 |
| C | 6.55025 | -1.63301 | -0.044 | H | -0.88335 | 0.341933 | 4.23588 |
| C | 5.489194 | -2.48521 | -0.37796 | H | -1.99922 | 1.620019 | 3.681699 |

**Table S27.** The Cartesian coordinates of the lowest energy conformers (Conf. C) for **1**.

| **Conf. A** | **X axis** | **Y axis** | **Z axis** | **Conf. A** | **X axis** | **Y axis** | **Z axis** |
| --- | --- | --- | --- | --- | --- | --- | --- |
| C | -2.21858 | 0.840225 | 1.090767 | C | 4.173323 | -2.022052 | -0.421387 |
| C | -1.68573 | 0.020183 | 2.246929 | C | 7.915614 | -2.240053 | -0.049388 |
| C | -0.510713 | -0.874756 | 1.853587 | O | 8.862113 | -1.336941 | 0.280792 |
| C | 0.422735 | -0.2098 | 0.91161 | O | 8.175777 | -3.400735 | -0.308897 |
| C | -0.00731 | 0.93043 | 0.131311 | C | -1.177625 | 0.940559 | 3.387051 |
| C | -1.315335 | 1.423072 | 0.193015 | H | -1.660192 | 2.153273 | -0.526998 |
| C | -3.60786 | 0.811103 | 1.174791 | H | 2.023334 | -1.562084 | 1.347836 |
| C | -3.968652 | -0.195076 | 2.180058 | H | 0.690084 | 2.248557 | -1.453265 |
| O | -2.807841 | -0.71417 | 2.737912 | H | 3.424218 | 0.550098 | -2.674976 |
| C | 1.68534 | -0.717101 | 0.760518 | H | 4.215101 | 2.766499 | -3.065436 |
| N | 2.590603 | -0.175237 | -0.100113 | H | 3.674246 | 5.572322 | -1.148919 |
| C | 2.220548 | 0.908112 | -0.899574 | H | -6.292127 | 2.392318 | 1.110699 |
| C | 0.963663 | 1.436276 | -0.789924 | H | -6.27076 | 0.640668 | 1.180428 |
| C | 3.157445 | 1.329676 | -1.962841 | H | -7.897863 | 1.587257 | -0.519937 |
| C | 3.635454 | 2.565023 | -2.170296 | H | -6.552305 | 2.352192 | -1.361203 |
| C | 3.497165 | 3.694755 | -1.21679 | H | -5.587388 | 0.120815 | -1.90372 |
| O | 3.749906 | 4.871405 | -1.825245 | H | -7.19616 | 0.346019 | -2.567984 |
| O | 3.236897 | 3.601305 | -0.030692 | H | -6.530319 | -1.292036 | -0.058784 |
| O | -5.044194 | -0.632152 | 2.547214 | H | -8.160592 | -0.940799 | -0.611429 |
| C | -4.520301 | 1.567377 | 0.312443 | H | -7.581378 | -3.160284 | -1.358427 |
| C | -6.026884 | 1.488844 | 0.540109 | H | -7.687944 | -2.12665 | -2.775813 |
| C | -6.834235 | 1.474132 | -0.7694 | H | -5.183585 | -1.967453 | -2.852959 |
| C | -6.645837 | 0.209868 | -1.625587 | H | -5.0728 | -2.936519 | -1.391803 |
| C | -7.127315 | -1.088232 | -0.958539 | H | -6.264067 | -3.90067 | -4.06752 |
| C | -7.088166 | -2.32362 | -1.874171 | H | -4.673263 | -4.345047 | -3.428441 |
| C | -5.680338 | -2.770891 | -2.293375 | H | -6.143925 | -4.882059 | -2.599528 |
| C | -5.688817 | -4.045414 | -3.144384 | H | 4.789761 | 1.20917 | 0.494428 |
| O | -4.072121 | 2.300464 | -0.577622 | H | 7.116552 | 0.334815 | 0.520844 |
| O | -0.322404 | -1.979483 | 2.335739 | H | 5.680129 | -3.546254 | -0.637125 |
| C | 3.943631 | -0.680437 | -0.106106 | H | 3.34655 | -2.66848 | -0.697087 |
| C | 4.994544 | 0.17562 | 0.235699 | H | 9.720554 | -1.801515 | 0.263059 |
| C | 6.295333 | -0.319324 | 0.252947 | H | -0.327608 | 1.546794 | 3.063994 |
| C | 6.5415 | -1.663839 | -0.05969 | H | -0.878103 | 0.323031 | 4.238853 |
| C | 5.475965 | -2.509833 | -0.392997 | H | -1.990827 | 1.606172 | 3.690083 |

**Table S28.** The Cartesian coordinates of the lowest energy conformers (Conf. D) for **1**.

| **Conf. A** | **X axis** | **Y axis** | **Z axis** | **Conf. A** | **X axis** | **Y axis** | **Z axis** |
| --- | --- | --- | --- | --- | --- | --- | --- |
| C | 1.985212 | -1.500463 | -0.933171 | C | -5.107932 | -1.341168 | 0.046685 |
| C | 1.356955 | -2.731891 | -0.312514 | C | -8.208287 | 0.102701 | 1.704681 |
| C | 0.082368 | -2.419179 | 0.470822 | O | -8.951465 | -1.017322 | 1.58691 |
| C | -0.764461 | -1.402825 | -0.20153 | O | -8.631786 | 1.122243 | 2.217923 |
| C | -0.198374 | -0.510123 | -1.190985 | C | 0.963513 | -3.758768 | -1.406094 |
| C | 1.158012 | -0.537348 | -1.528102 | H | 1.587694 | 0.247044 | -2.137266 |
| C | 3.356771 | -1.600595 | -0.735132 | H | -2.489878 | -1.893083 | 0.964343 |
| C | 3.596188 | -2.71759 | 0.181745 | H | -0.765791 | 1.130391 | -2.503458 |
| O | 2.380956 | -3.316206 | 0.493327 | H | -4.20241 | 0.843845 | -2.548709 |
| C | -2.071327 | -1.270448 | 0.183077 | H | -4.058693 | 3.205319 | -2.848871 |
| N | -2.905713 | -0.346374 | -0.368369 | H | -1.745232 | 5.37127 | -1.510595 |
| C | -2.419759 | 0.526004 | -1.345164 | H | 5.893261 | -1.054144 | 0.198091 |
| C | -1.109721 | 0.453336 | -1.730224 | H | 6.131067 | -1.855026 | -1.317195 |
| C | -3.396809 | 1.382096 | -2.051175 | H | 6.576352 | 0.415899 | -2.417087 |
| C | -3.344217 | 2.713987 | -2.19624 | H | 7.801216 | -0.143749 | -1.284016 |
| C | -2.412423 | 3.606706 | -1.462018 | H | 7.238962 | 2.312202 | -1.109743 |
| O | -2.3449 | 4.819089 | -2.049034 | H | 5.611626 | 1.890955 | -0.602137 |
| O | -1.811205 | 3.327532 | -0.440446 | H | 6.563239 | 0.733624 | 1.430134 |
| O | 4.618355 | -3.145414 | 0.683785 | H | 8.187419 | 1.13433 | 0.89092 |
| C | 4.36631 | -0.675853 | -1.261652 | H | 7.607442 | 3.583667 | 1.006375 |
| C | 5.827527 | -0.891221 | -0.882947 | H | 6.006715 | 3.173448 | 1.604836 |
| C | 6.76847 | 0.223566 | -1.355349 | H | 7.618345 | 3.876052 | 3.39763 |
| C | 6.654001 | 1.547597 | -0.578568 | H | 8.645932 | 2.502443 | 3.02215 |
| C | 7.143205 | 1.48269 | 0.875605 | H | 6.813503 | 0.921396 | 3.745739 |
| C | 7.054524 | 2.836851 | 1.59438 | H | 7.202222 | 2.067065 | 5.033902 |
| C | 7.599469 | 2.840125 | 3.032499 | H | 5.746928 | 2.30018 | 4.051858 |
| C | 6.796966 | 1.982525 | 4.018778 | H | -3.989926 | 1.798367 | 0.790684 |
| O | 4.033054 | 0.243636 | -2.017131 | H | -6.315562 | 1.965228 | 1.684711 |
| O | -0.236087 | -3.009201 | 1.490193 | H | -7.067769 | -2.095899 | 0.497924 |
| C | -4.256363 | -0.237266 | 0.12931 | H | -4.768926 | -2.25782 | -0.424848 |
| C | -4.675126 | 0.960469 | 0.717945 | H | -9.825205 | -0.824358 | 1.977345 |
| C | -5.968711 | 1.049377 | 1.219394 | H | 1.851229 | -3.999601 | -1.997806 |
| C | -6.835779 | -0.049468 | 1.145168 | H | 0.590718 | -4.668074 | -0.925722 |
| C | -6.399113 | -1.245616 | 0.560255 | H | 0.193594 | -3.360459 | -2.071702 |

**Table S29.** The Cartesian coordinates of the lowest energy conformers (Conf. E) for **1**.

| **Conf. A** | **X axis** | **Y axis** | **Z axis** | **Conf. A** | **X axis** | **Y axis** | **Z axis** |
| --- | --- | --- | --- | --- | --- | --- | --- |
| C | -1.947467 | 1.524018 | -0.715952 | C | 4.56148 | 0.167549 | 1.949727 |
| C | -1.14842 | 2.796035 | -0.518344 | C | 8.30883 | -0.088845 | 1.661273 |
| C | 0.08967 | 2.591066 | 0.354451 | O | 9.137382 | -0.331235 | 0.624272 |
| C | 0.777928 | 1.306617 | 0.076553 | O | 8.711238 | 0.103275 | 2.794144 |
| C | 0.079134 | 0.223636 | -0.583331 | C | -0.645184 | 3.346354 | -1.878064 |
| C | -1.268544 | 0.318334 | -0.94207 | H | -1.811406 | -0.557954 | -1.271119 |
| C | -3.288966 | 1.862807 | -0.590658 | H | 2.610926 | 1.952805 | 0.972427 |
| C | -3.35855 | 3.239904 | -0.096227 | H | 0.354984 | -1.856861 | -1.159304 |
| O | -2.068465 | 3.742277 | 0.027631 | H | 3.144411 | -2.759123 | 0.651566 |
| C | 2.07322 | 1.146154 | 0.489264 | H | 3.515188 | -4.15375 | -1.247525 |
| N | 2.765445 | -0.009127 | 0.288167 | H | 2.772025 | -3.482819 | -4.539738 |
| C | 2.132452 | -1.096584 | -0.318618 | H | -6.020851 | 2.285295 | -1.240947 |
| C | 0.83414 | -0.983418 | -0.732445 | H | -5.84736 | 1.959061 | 0.453234 |
| C | 2.848545 | -2.389822 | -0.329363 | H | -7.896956 | 0.903156 | -0.694126 |
| C | 3.093514 | -3.164766 | -1.395814 | H | -6.795738 | -0.157649 | -1.567611 |
| C | 2.898929 | -2.746041 | -2.807044 | H | -7.077356 | -0.020679 | 1.484701 |
| O | 2.872779 | -3.816419 | -3.627042 | H | -5.967139 | -1.076669 | 0.617256 |
| O | 2.816017 | -1.598948 | -3.207758 | H | -9.01843 | -1.114927 | 0.30108 |
| O | -4.304262 | 3.938675 | 0.215297 | H | -7.905031 | -2.222423 | -0.486938 |
| C | -4.422818 | 0.956736 | -0.801008 | H | -8.321275 | -2.001478 | 2.548598 |
| C | -5.83238 | 1.469655 | -0.528077 | H | -9.078842 | -3.199336 | 1.509976 |
| C | -6.923215 | 0.398933 | -0.631038 | H | -6.806658 | -4.093362 | 0.893193 |
| C | -6.943802 | -0.583903 | 0.54838 | H | -6.060737 | -2.941624 | 1.989982 |
| C | -8.057509 | -1.633843 | 0.430368 | H | -8.046541 | -5.139031 | 2.83281 |
| C | -8.171877 | -2.589168 | 1.630086 | H | -6.295647 | -5.167924 | 3.101034 |
| C | -6.972995 | -3.528918 | 1.822269 | H | -7.300938 | -3.971609 | 3.934444 |
| C | -7.162825 | -4.507068 | 2.986691 | H | 4.780898 | -0.438916 | -1.40336 |
| O | -4.232396 | -0.193026 | -1.212191 | H | 7.198897 | -0.48121 | -0.829895 |
| O | 0.507576 | 3.436836 | 1.128236 | H | 6.246448 | 0.321651 | 3.282992 |
| C | 4.16817 | -0.054688 | 0.627549 | H | 3.816759 | 0.33372 | 2.721272 |
| C | 5.10861 | -0.287483 | -0.380251 | H | 10.048585 | -0.313432 | 0.974146 |
| C | 6.461816 | -0.306255 | -0.055203 | H | 0.049648 | 2.653074 | -2.358685 |
| C | 6.871923 | -0.083569 | 1.267044 | H | -0.143501 | 4.304278 | -1.712891 |
| C | 5.916869 | 0.155485 | 2.263522 | H | -1.505498 | 3.496686 | -2.536465 |

**Table S30.** The Cartesian coordinates of the lowest energy conformers (Conf. A) for **2**.

| **Conf. A** | **X axis** | **Y axis** | **Z axis** | **Conf. A** | **X axis** | **Y axis** | **Z axis** |
| --- | --- | --- | --- | --- | --- | --- | --- |
| C | 0.793139 | -1.432782 | -0.904159 | O | 4.013465 | -2.191152 | 0.255961 |
| C | 0.597028 | -2.516895 | 0.135581 | C | 0.181279 | -3.851746 | -0.53628 |
| C | -0.494448 | -2.184605 | 1.151726 | H | -0.225809 | -0.150107 | -2.305656 |
| C | -1.664695 | -1.514815 | 0.53359 | H | -2.92722 | -1.915268 | 2.208651 |
| C | -1.54562 | -0.864579 | -0.750778 | H | -2.734603 | 0.259816 | -2.183216 |
| C | -0.327767 | -0.793331 | -1.441664 | H | 4.552846 | -0.953082 | -3.146057 |
| C | 2.167968 | -1.295902 | -1.094905 | H | 4.788635 | -0.914023 | -1.404526 |
| C | 2.838866 | -2.06315 | -0.041207 | H | 4.351215 | 1.514195 | -3.24076 |
| O | 1.880283 | -2.704517 | 0.733545 | H | 5.921276 | 0.987975 | -2.640572 |
| C | -2.845955 | -1.461867 | 1.227583 | H | 3.604739 | 2.020968 | -0.909424 |
| N | -3.961002 | -0.859755 | 0.742101 | H | 5.004761 | 2.956145 | -1.405087 |
| C | -3.905141 | -0.232001 | -0.496695 | H | 5.088021 | 0.478111 | 0.404858 |
| C | -2.737627 | -0.226738 | -1.21466 | H | 6.504923 | 1.358575 | -0.15145 |
| C | 2.794281 | -0.433254 | -2.097369 | H | 4.236369 | 2.55825 | 1.533983 |
| C | 4.313824 | -0.387762 | -2.232753 | H | 5.660021 | 3.434319 | 0.989483 |
| C | 4.854223 | 1.046028 | -2.386929 | H | 5.701726 | 0.985059 | 2.836482 |
| C | 4.675306 | 1.939307 | -1.147554 | H | 7.125297 | 1.859017 | 2.292407 |
| C | 5.439398 | 1.473126 | 0.100114 | H | 6.301043 | 3.943677 | 3.463034 |
| C | 5.301787 | 2.436938 | 1.286244 | H | 4.86678 | 3.063053 | 4.011103 |
| C | 6.060775 | 1.980632 | 2.539013 | H | 6.470971 | 2.593608 | 4.596828 |
| C | 5.91809 | 2.947787 | 3.71894 | H | -5.918641 | -0.421948 | -1.33159 |
| O | 2.095817 | 0.242854 | -2.864028 | H | -6.257858 | 1.857248 | -1.982647 |
| O | -0.437634 | -2.515906 | 2.324984 | H | -3.992963 | 4.452696 | -1.614082 |
| C | -5.152204 | 0.311719 | -1.085269 | H | -6.040277 | -0.674185 | 1.000294 |
| C | -5.364311 | 1.589353 | -1.427963 | H | -5.220099 | -1.721945 | 2.163115 |
| C | -4.458959 | 2.708821 | -1.066859 | H | -4.12119 | 0.342978 | 3.166906 |
| O | -4.588916 | 3.738989 | -1.915822 | H | -5.880021 | 0.248712 | 3.343993 |
| O | -3.703265 | 2.733385 | -0.105577 | H | -4.52078 | 1.900403 | 1.467879 |
| C | -5.151043 | -0.774743 | 1.622811 | H | 0.935081 | -4.119115 | -1.28227 |
| C | -5.073582 | 0.393483 | 2.617444 | H | 0.130832 | -4.635572 | 0.225154 |
| O | -5.281785 | 1.660913 | 2.031127 | H | -0.789442 | -3.765961 | -1.031074 |

**Table S31.** The Cartesian coordinates of the lowest energy conformers (Conf. B) for **2**.

| **Conf. A** | **X axis** | **Y axis** | **Z axis** | **Conf. A** | **X axis** | **Y axis** | **Z axis** |
| --- | --- | --- | --- | --- | --- | --- | --- |
| C | 0.964252 | -1.482917 | -0.778757 | O | -5.329676 | 1.537405 | 1.781256 |
| C | 0.695984 | -2.576609 | 0.234792 | C | 0.325999 | -3.904299 | -0.476949 |
| C | -0.46276 | -2.253057 | 1.176253 | H | 0.046568 | -0.178992 | -2.229513 |
| C | -1.587888 | -1.578253 | 0.484092 | H | -2.969052 | -2.010569 | 2.054471 |
| C | -1.378797 | -0.910787 | -0.779667 | H | -2.461442 | 0.244074 | -2.270977 |
| C | -0.11584 | -0.833509 | -1.383432 | H | -5.991648 | -0.775331 | 0.62983 |
| C | -2.817198 | -1.540721 | 1.089869 | H | -5.25532 | -1.847686 | 1.825377 |
| N | -3.896446 | -0.934871 | 0.53345 | H | -5.695865 | -0.439841 | -1.675471 |
| C | -3.750794 | -0.280774 | -0.684054 | H | -5.981921 | 1.860851 | -2.278161 |
| C | -2.534366 | -0.262456 | -1.31545 | H | -3.741828 | 4.431366 | -1.658715 |
| C | 2.349231 | -1.351129 | -0.876905 | H | 4.98523 | -0.969652 | -1.00213 |
| C | 2.945475 | -2.127135 | 0.214405 | H | 4.849176 | -1.092497 | -2.747826 |
| O | 1.935015 | -2.771916 | 0.917522 | H | 4.707229 | 1.375262 | -2.973717 |
| O | -0.487243 | -2.59441 | 2.347741 | H | 6.230228 | 0.843842 | -2.265263 |
| O | 4.096774 | -2.258247 | 0.590643 | H | 3.850574 | 2.00961 | -0.721898 |
| C | -5.153666 | -0.884964 | 1.318466 | H | 5.303414 | 2.886632 | -1.169837 |
| C | -4.950069 | 0.283301 | -1.348487 | H | 6.679861 | 1.260104 | 0.200636 |
| C | -5.13362 | 1.571757 | -1.665975 | H | 5.196569 | 0.507201 | 0.766343 |
| C | -4.255455 | 2.675108 | -1.202419 | H | 5.960677 | 3.447032 | 1.207042 |
| O | -4.314402 | 3.730366 | -2.028 | H | 6.172546 | 2.157279 | 2.382002 |
| O | -3.577575 | 2.667077 | -0.184498 | H | 3.472989 | 3.121672 | 1.291947 |
| C | 3.045678 | -0.487305 | -1.83117 | H | 3.681982 | 1.780178 | 2.406789 |
| C | 4.571025 | -0.474251 | -1.880553 | H | 4.498029 | 4.698284 | 2.978682 |
| C | 5.152781 | 0.937361 | -2.073391 | H | 4.699116 | 3.349835 | 4.107295 |
| C | 4.929702 | 1.891534 | -0.887513 | H | 3.074181 | 3.878465 | 3.639829 |
| C | 5.619557 | 1.458749 | 0.415801 | H | -6.03347 | 0.087618 | 2.99777 |
| C | 5.534308 | 2.492542 | 1.551598 | H | -4.26655 | 0.190243 | 2.976506 |
| O | 2.403683 | 0.208472 | -2.628836 | H | -4.52421 | 1.792383 | 1.29139 |
| C | 4.118901 | 2.739422 | 2.09328 | H | 0.222268 | -4.695222 | 0.271658 |
| C | 4.094147 | 3.720578 | 3.270301 | H | 1.129247 | -4.165815 | -1.171635 |
| C | -5.167897 | 0.254484 | 2.348104 | H | -0.608211 | -3.812216 | -1.036635 |

**Table S32.** The Cartesian coordinates of the lowest energy conformers (Conf. C) for **2**.

| **Conf. A** | **X axis** | **Y axis** | **Z axis** | **Conf. A** | **X axis** | **Y axis** | **Z axis** |
| --- | --- | --- | --- | --- | --- | --- | --- |
| C | 0.64572 | -1.666674 | -0.71059 | O | -5.363856 | 1.838447 | 1.883696 |
| C | 0.14864 | -2.853426 | 0.090787 | C | -0.322776 | -3.990785 | -0.852573 |
| C | -1.040315 | -2.515301 | 0.98915 | H | 0.036314 | -0.048294 | -2.000485 |
| C | -1.99837 | -1.584989 | 0.344118 | H | -3.556528 | -2.042213 | 1.730986 |
| C | -1.591482 | -0.770394 | -0.778398 | H | -2.375795 | 0.742342 | -2.130007 |
| C | -0.283892 | -0.788761 | -1.279345 | H | -6.245188 | -0.159304 | 0.352557 |
| C | -3.258663 | -1.45733 | 0.868481 | H | -5.763018 | -1.490426 | 1.409748 |
| N | -4.192243 | -0.617963 | 0.353201 | H | -5.716638 | 0.463601 | -1.847267 |
| C | -3.85477 | 0.183735 | -0.731593 | H | -5.627948 | 2.842116 | -2.108395 |
| C | -2.598698 | 0.115232 | -1.274509 | H | -3.112106 | 4.934603 | -0.967132 |
| C | 2.036217 | -1.708753 | -0.67524 | H | 4.738467 | -1.936042 | -1.501094 |
| C | 2.428272 | -2.720167 | 0.306534 | H | 4.626239 | -0.941352 | -0.090639 |
| O | 1.284488 | -3.324913 | 0.817352 | H | 6.322526 | -0.189881 | -1.888238 |
| O | -1.216471 | -3.027913 | 2.082936 | H | 4.958131 | 0.177171 | -2.937019 |
| O | 3.519561 | -3.064013 | 0.721309 | H | 4.13859 | 1.860196 | -1.235745 |
| C | -5.487758 | -0.49143 | 1.063035 | H | 5.681999 | 2.249605 | -1.976974 |
| C | -4.905165 | 1.011586 | -1.370587 | H | 5.352668 | 1.01819 | 0.811159 |
| C | -4.880674 | 2.345059 | -1.498068 | H | 6.890732 | 1.381456 | 0.041915 |
| C | -3.899927 | 3.227489 | -0.817884 | H | 6.229356 | 3.807523 | -0.107786 |
| O | -3.742497 | 4.386311 | -1.474598 | H | 4.720442 | 3.44478 | 0.716951 |
| O | -3.317952 | 2.973796 | 0.227451 | H | 6.487762 | 4.429073 | 2.204793 |
| C | 2.936306 | -0.813511 | -1.406301 | H | 7.521708 | 3.041439 | 1.90629 |
| C | 4.43865 | -0.931393 | -1.170056 | H | 4.760683 | 2.915554 | 3.272408 |
| C | 5.273418 | 0.135877 | -1.888131 | H | 5.841191 | 1.533419 | 3.038885 |
| C | 5.190247 | 1.550051 | -1.285689 | H | 6.321234 | 2.856351 | 4.107771 |
| C | 5.837864 | 1.697973 | 0.098782 | H | -6.350883 | 0.34743 | 2.821487 |
| C | 5.773579 | 3.135913 | 0.634121 | H | -4.589794 | 0.191054 | 2.912789 |
| O | 2.481041 | 0.004784 | -2.213965 | H | -4.494469 | 2.041255 | 1.487681 |
| C | 6.47008 | 3.35224 | 1.988085 | H | -0.596887 | -4.863442 | -0.252522 |
| C | 5.81267 | 2.621524 | 3.165391 | H | 0.500339 | -4.255584 | -1.522353 |
| C | -5.423583 | 0.477114 | 2.253579 | H | -1.18148 | -3.682152 | -1.45422 |

**Table S33.** The Cartesian coordinates of the lowest energy conformers (Conf. D) for **2**.

| **Conf. A** | **X axis** | **Y axis** | **Z axis** | **Conf. A** | **X axis** | **Y axis** | **Z axis** |
| --- | --- | --- | --- | --- | --- | --- | --- |
| C | 1.159029 | -1.524018 | -0.819755 | O | -5.193321 | 1.372802 | 1.722598 |
| C | 0.882283 | -2.653532 | 0.152091 | C | 0.534209 | -3.95851 | -0.61069 |
| C | -0.295384 | -2.367641 | 1.082307 | H | 0.25664 | -0.189681 | -2.253261 |
| C | -1.412851 | -1.678498 | 0.391732 | H | -2.816929 | -2.168785 | 1.924174 |
| C | -1.187494 | -0.970542 | -0.847626 | H | -2.25435 | 0.22141 | -2.321344 |
| C | 0.084208 | -0.86671 | -1.426957 | H | -5.100787 | -2.011723 | 1.662522 |
| C | -2.652361 | -1.667955 | 0.977316 | H | -5.823589 | -0.905447 | 0.489945 |
| N | -3.726563 | -1.052172 | 0.421975 | H | -5.493109 | -0.502153 | -1.800234 |
| C | -3.565547 | -0.360177 | -0.772735 | H | -5.787591 | 1.813885 | -2.335726 |
| C | -2.339334 | -0.314485 | -1.382974 | H | -3.58135 | 4.381838 | -1.599369 |
| C | 2.542044 | -1.369078 | -0.877738 | H | 5.139329 | 0.036924 | -2.616152 |
| C | 3.127031 | -2.183875 | 0.188581 | H | 5.225795 | -1.269393 | -1.406141 |
| O | 2.111073 | -2.865267 | 0.849141 | H | 4.762029 | 0.357032 | 0.414419 |
| O | -0.33805 | -2.745742 | 2.242029 | H | 6.246423 | 0.704768 | -0.453962 |
| O | 4.276284 | -2.322028 | 0.566042 | H | 3.585986 | 2.165714 | -0.912315 |
| C | -4.996048 | -1.0329 | 1.188075 | H | 5.059337 | 2.452961 | -1.812596 |
| C | -4.758078 | 0.215806 | -1.439093 | H | 4.854527 | 4.17708 | -0.128903 |
| C | -4.946793 | 1.512182 | -1.719303 | H | 6.245385 | 3.172172 | 0.246825 |
| C | -4.084912 | 2.607164 | -1.207974 | H | 4.945126 | 2.00304 | 2.039216 |
| O | -4.142681 | 3.688555 | -1.999183 | H | 5.077533 | 3.732198 | 2.294941 |
| O | -3.420358 | 2.571727 | -0.181861 | H | 2.752512 | 4.035241 | 1.355795 |
| C | 3.259818 | -0.473819 | -1.786263 | H | 2.604898 | 2.2926 | 1.18963 |
| C | 4.765051 | -0.307107 | -1.645501 | H | 3.04441 | 3.773278 | 3.853185 |
| C | 5.151533 | 0.711421 | -0.546662 | H | 2.908578 | 2.016924 | 3.681985 |
| C | 4.679826 | 2.144385 | -0.828167 | H | 1.521034 | 3.051796 | 3.307881 |
| C | 5.146167 | 3.174633 | 0.216166 | H | -5.907065 | -0.117497 | 2.883153 |
| C | 4.613748 | 2.974726 | 1.646623 | H | -4.140481 | -0.004491 | 2.891949 |
| O | 2.646517 | 0.159951 | -2.655395 | H | -4.382077 | 1.646678 | 1.252874 |
| C | 3.088366 | 3.079406 | 1.783717 | H | -0.389852 | -3.852776 | -1.184595 |
| C | 2.612287 | 2.975002 | 3.236766 | H | 0.421552 | -4.774919 | 0.108672 |
| C | -5.032724 | 0.073873 | 2.252256 | H | 1.351892 | -4.191971 | -1.298521 |

**Table S34.** The Cartesian coordinates of the lowest energy conformers (Conf. E) for **2**.

| **Conf. A** | **X axis** | **Y axis** | **Z axis** | **Conf. A** | **X axis** | **Y axis** | **Z axis** |
| --- | --- | --- | --- | --- | --- | --- | --- |
| C | -0.402096 | -1.471378 | 0.484284 | O | -2.724427 | -4.064641 | 0.098297 |
| C | 0.428467 | -2.702721 | 0.182825 | C | 1.20552 | -3.162011 | 1.444195 |
| C | 1.473058 | -2.466946 | -0.908042 | H | -0.308378 | 0.638894 | 0.921396 |
| C | 2.115939 | -1.134686 | -0.802649 | H | 3.77307 | -1.708333 | -2.021833 |
| C | 1.477881 | -0.06596 | -0.067875 | H | 1.727236 | 2.048475 | 0.377976 |
| C | 0.223924 | -0.220926 | 0.536305 | H | -4.409313 | -2.231451 | 0.127359 |
| C | -1.719349 | -1.896958 | 0.628627 | H | -4.245546 | -2.425707 | 1.843207 |
| C | -1.787519 | -3.294694 | 0.201383 | H | -5.112166 | -0.011085 | 2.137066 |
| O | -0.512235 | -3.724075 | -0.15141 | H | -6.281385 | -1.196646 | 1.56251 |
| C | 3.298369 | -0.912139 | -1.459782 | H | -4.762268 | 0.772455 | -0.226701 |
| N | 3.937409 | 0.285205 | -1.444817 | H | -5.936642 | -0.41172 | -0.790505 |
| C | 3.360418 | 1.350631 | -0.762334 | H | -7.715443 | 0.697038 | 0.604421 |
| C | 2.168304 | 1.185558 | -0.1075 | H | -6.540704 | 1.883114 | 1.156035 |
| C | -2.850294 | -1.052116 | 1.019171 | H | -6.20738 | 2.655104 | -1.216371 |
| C | -4.247827 | -1.66302 | 1.050744 | H | -7.385107 | 1.471455 | -1.766433 |
| C | -5.371833 | -0.648181 | 1.282904 | H | -7.978896 | 3.769563 | 0.177082 |
| C | -5.673131 | 0.232302 | 0.062035 | H | -9.155969 | 2.586432 | -0.371995 |
| C | -6.805386 | 1.238333 | 0.304592 | H | -8.855562 | 3.382594 | -2.753153 |
| C | -7.117576 | 2.115121 | -0.914904 | H | -7.669156 | 4.574116 | -2.200383 |
| C | -8.246318 | 3.12562 | -0.672968 | H | -9.360319 | 4.707097 | -1.691116 |
| C | -8.551107 | 3.996559 | -1.896285 | H | 3.985266 | 3.137319 | -1.86052 |
| O | -2.666519 | 0.12837 | 1.338376 | H | 4.741125 | 4.453929 | -0.0077 |
| O | 1.800068 | -3.32194 | -1.715372 | H | 4.683834 | 3.522629 | 3.328092 |
| C | 3.981905 | 2.692138 | -0.866486 | H | 5.48279 | 1.393991 | -2.342354 |
| C | 4.434286 | 3.426317 | 0.15895 | H | 5.287284 | -0.258796 | -2.936534 |
| C | 4.586593 | 2.916275 | 1.544545 | H | 6.139625 | -1.109331 | -0.69504 |
| O | 4.54939 | 3.912593 | 2.44181 | H | 7.312313 | -0.209964 | -1.669975 |
| O | 4.759866 | 1.747299 | 1.85972 | H | 5.941976 | 0.83205 | 0.595507 |
| C | 5.293239 | 0.364165 | -2.03876 | H | 1.729863 | -4.095997 | 1.221815 |
| C | 6.395435 | -0.110114 | -1.07954 | H | 0.490528 | -3.332098 | 2.2542 |
| O | 6.682912 | 0.801201 | -0.039962 | H | 1.929616 | -2.409027 | 1.765381 |

**Table S35.** The Cartesian coordinates of the lowest energy conformers (Conf. F) for **2**.

| **Conf. A** | **X axis** | **Y axis** | **Z axis** | **Conf. A** | **X axis** | **Y axis** | **Z axis** |
| --- | --- | --- | --- | --- | --- | --- | --- |
| C | -0.885566 | -0.333303 | -1.141467 | O | 6.390507 | 0.916423 | -0.166395 |
| C | -0.555522 | 0.936262 | -1.898874 | C | 0.205244 | 0.610824 | -3.210879 |
| C | 0.335402 | 1.89248 | -1.106707 | H | -0.052409 | -1.94369 | 0.02362 |
| C | 1.388796 | 1.183775 | -0.339773 | H | 2.57756 | 2.944618 | -0.114835 |
| C | 1.247917 | -0.218269 | -0.022015 | H | 2.234315 | -1.79255 | 1.108928 |
| C | 0.111058 | -0.954762 | -0.383591 | H | 5.148504 | 1.754722 | 2.034667 |
| C | 2.47331 | 1.889943 | 0.11272 | H | 4.338926 | 3.157862 | 1.327414 |
| N | 3.464394 | 1.327272 | 0.849615 | H | 4.387164 | -0.175837 | 3.126844 |
| C | 3.370782 | -0.015578 | 1.195757 | H | 5.782418 | -2.062403 | 2.644581 |
| C | 2.295248 | -0.759765 | 0.785739 | H | 5.884079 | -3.827697 | -0.334141 |
| C | -2.232817 | -0.608538 | -1.375571 | H | -4.836419 | -1.09825 | -1.675236 |
| C | -2.815541 | 0.564453 | -2.033685 | H | -4.433309 | -2.763504 | -2.064954 |
| O | -1.816907 | 1.506292 | -2.24983 | H | -6.228559 | -2.815369 | -0.431902 |
| O | 0.240788 | 3.10714 | -1.175851 | H | -4.758152 | -3.461551 | 0.291796 |
| O | -3.9561 | 0.832959 | -2.367276 | H | -4.367773 | -1.303839 | 1.48094 |
| C | 4.67433 | 2.136573 | 1.130264 | H | -5.883068 | -2.068195 | 1.927652 |
| C | 4.376304 | -0.591022 | 2.119992 | H | -5.600443 | 0.30515 | 0.001673 |
| C | 5.18203 | -1.627351 | 1.851992 | H | -7.103771 | -0.549148 | 0.313668 |
| C | 5.349595 | -2.224558 | 0.50336 | H | -7.089751 | 1.450645 | 1.662454 |
| O | 5.763292 | -3.498144 | 0.578208 | H | -6.95787 | 0.072223 | 2.744745 |
| O | 5.173825 | -1.64656 | -0.560069 | H | -4.601943 | 1.810075 | 1.828287 |
| C | -2.935725 | -1.808802 | -0.920989 | H | -4.491262 | 0.480092 | 2.970703 |
| C | -4.409638 | -2.012017 | -1.261333 | H | -4.579245 | 2.660358 | 4.188077 |
| C | -5.228924 | -2.540118 | -0.069376 | H | -6.022447 | 1.704833 | 4.563158 |
| C | -5.368171 | -1.553862 | 1.103089 | H | -6.126311 | 3.050578 | 3.418289 |
| C | -6.141219 | -0.269005 | 0.76644 | H | 5.163147 | 2.411898 | -0.961619 |
| C | -6.413888 | 0.641737 | 1.975784 | H | 6.421869 | 2.904604 | 0.182071 |
| O | -2.332612 | -2.682825 | -0.284414 | H | 5.795704 | 0.2099 | -0.483769 |
| C | -5.161566 | 1.269063 | 2.605125 | H | 1.17518 | 0.149716 | -3.008308 |
| C | -5.488557 | 2.224721 | 3.757714 | H | 0.355502 | 1.534971 | -3.776655 |
| C | 5.679359 | 2.12867 | -0.031554 | H | -0.398177 | -0.08269 | -3.803429 |

**Table S36.** The Cartesian coordinates of the lowest energy conformers (Conf. G) for **2**.

| **Conf. A** | **X axis** | **Y axis** | **Z axis** | **Conf. A** | **X axis** | **Y axis** | **Z axis** |
| --- | --- | --- | --- | --- | --- | --- | --- |
| C | 0.885886 | -1.614624 | -0.687974 | O | 3.752186 | -2.979161 | 0.791108 |
| C | 0.403571 | -2.857631 | 0.032964 | C | 0.017151 | -3.963622 | -0.983645 |
| C | -0.834427 | -2.608547 | 0.893615 | H | 0.265641 | 0.0362 | -1.930822 |
| C | -1.801586 | -1.686427 | 0.250249 | H | -3.393734 | -2.261745 | 1.55138 |
| C | -1.382101 | -0.80724 | -0.817576 | H | -2.174553 | 0.722326 | -2.145319 |
| C | -0.054034 | -0.748587 | -1.257981 | H | 5.01171 | -1.678697 | -1.376489 |
| C | 2.274223 | -1.605306 | -0.593688 | H | 4.819962 | -0.829169 | 0.118494 |
| C | 2.665715 | -2.652122 | 0.350782 | H | 5.196383 | 0.564173 | -2.598734 |
| O | 1.52619 | -3.321969 | 0.784242 | H | 6.53815 | 0.156242 | -1.534679 |
| C | -3.086104 | -1.630124 | 0.726096 | H | 4.244497 | 2.047106 | -0.799064 |
| N | -4.031767 | -0.807421 | 0.205856 | H | 5.804401 | 2.56525 | -1.416199 |
| C | -3.68485 | 0.047939 | -0.834209 | H | 6.944493 | 1.49306 | 0.555338 |
| C | -2.405204 | 0.054175 | -1.323622 | H | 5.382898 | 1.07922 | 1.235083 |
| C | 3.166089 | -0.636838 | -1.236159 | H | 6.401614 | 3.913264 | 0.655425 |
| C | 4.663736 | -0.722041 | -0.960112 | H | 6.379235 | 3.140945 | 2.234029 |
| C | 5.476563 | 0.438572 | -1.546608 | H | 4.546859 | 4.779496 | 1.948592 |
| C | 5.308963 | 1.785339 | -0.819543 | H | 3.90012 | 3.887321 | 0.585935 |
| C | 5.893938 | 1.815338 | 0.60064 | H | 4.095307 | 2.79347 | 3.460723 |
| C | 5.842391 | 3.197892 | 1.275885 | H | 3.3703 | 1.934302 | 2.094008 |
| C | 4.436135 | 3.767078 | 1.536796 | H | 2.622931 | 3.416957 | 2.69858 |
| C | 3.584602 | 2.929999 | 2.49886 | H | -5.507886 | 0.28086 | -2.021023 |
| O | 2.709059 | 0.217178 | -2.00493 | H | -5.522719 | 2.668806 | -2.199288 |
| O | -1.035438 | -3.178777 | 1.954055 | H | -3.166349 | 4.838395 | -0.881334 |
| C | -4.744596 | 0.84937 | -1.49125 | H | -6.099011 | -0.423802 | 0.141172 |
| C | -4.778653 | 2.186203 | -1.573656 | H | -5.607754 | -1.777145 | 1.166096 |
| C | -3.870056 | 3.089597 | -0.824367 | H | -4.558443 | -0.110666 | 2.775889 |
| O | -3.749184 | 4.279284 | -1.43199 | H | -6.319233 | -0.016998 | 2.620084 |
| O | -3.313372 | 2.825841 | 0.232155 | H | -4.48233 | 1.794071 | 1.419049 |
| C | -5.357445 | -0.756148 | 0.867959 | H | -0.827305 | -3.657881 | -1.606538 |
| C | -5.376525 | 0.168449 | 2.094547 | H | -0.246797 | -4.874758 | -0.438808 |
| O | -5.356136 | 1.543956 | 1.776407 | H | 0.877264 | -4.163633 | -1.628984 |

**Table S37.** The Cartesian coordinates of the lowest energy conformers (Conf. H) for **2**.

| **Conf. A** | **X axis** | **Y axis** | **Z axis** | **Conf. A** | **X axis** | **Y axis** | **Z axis** |
| --- | --- | --- | --- | --- | --- | --- | --- |
| C | 0.238529 | -1.931349 | -0.55215 | O | -5.29496 | 2.394685 | 1.792846 |
| C | -0.612399 | -3.086712 | -0.063289 | C | -1.12651 | -3.930908 | -1.258598 |
| C | -1.848937 | -2.633933 | 0.711704 | H | 0.188397 | -0.042126 | -1.593621 |
| C | -2.480948 | -1.427371 | 0.124985 | H | -4.309254 | -1.727258 | 1.186061 |
| C | -1.736151 | -0.564078 | -0.763449 | H | -1.970383 | 1.263498 | -1.919394 |
| C | -0.389485 | -0.793801 | -1.07205 | H | -6.28007 | -0.649368 | 0.683434 |
| C | -3.761785 | -1.102382 | 0.490004 | H | -6.299857 | 0.898334 | -0.168943 |
| N | -4.411458 | -0.010309 | 0.013702 | H | -5.307225 | 1.677165 | -2.143438 |
| C | -3.74574 | 0.845755 | -0.856728 | H | -4.722112 | 3.992949 | -1.975615 |
| C | -2.45295 | 0.583293 | -1.226993 | H | -2.079702 | 5.315847 | -0.169767 |
| C | 1.566166 | -2.286833 | -0.334086 | H | 4.121912 | -2.226678 | 0.727115 |
| C | 1.576397 | -3.501245 | 0.48202 | H | 4.271208 | -2.979928 | -0.827403 |
| O | 0.265383 | -3.906091 | 0.710073 | H | 5.15486 | -0.737262 | -1.758379 |
| O | -2.307296 | -3.25441 | 1.657788 | H | 6.210988 | -1.593096 | -0.638787 |
| O | 2.4904 | -4.14173 | 0.967227 | H | 4.391262 | 0.75376 | 0.113133 |
| C | -5.753863 | 0.300392 | 0.56111 | H | 5.441153 | -0.111069 | 1.231231 |
| C | -4.494861 | 1.964114 | -1.477009 | H | 7.445819 | 0.579895 | -0.125678 |
| C | -4.194569 | 3.265166 | -1.367366 | H | 6.394165 | 1.452598 | -1.231736 |
| C | -3.187368 | 3.810402 | -0.423313 | H | 6.694935 | 2.066017 | 1.76104 |
| O | -2.713582 | 4.993945 | -0.84063 | H | 5.642493 | 2.938212 | 0.655475 |
| O | -2.836462 | 3.289047 | 0.625831 | H | 8.694664 | 2.758785 | 0.403194 |
| C | 2.737004 | -1.510618 | -0.748847 | H | 7.642989 | 3.631299 | -0.701085 |
| C | 4.123673 | -2.002297 | -0.346437 | H | 7.973005 | 4.271981 | 2.29647 |
| C | 5.262312 | -1.044207 | -0.710766 | H | 8.669918 | 5.201669 | 0.95966 |
| C | 5.337874 | 0.202742 | 0.181481 | H | 6.913719 | 5.151128 | 1.183898 |
| C | 6.49852 | 1.137127 | -0.182749 | H | -6.704307 | 1.047303 | 2.316973 |
| C | 6.589274 | 2.380231 | 0.711711 | H | -5.050814 | 0.482218 | 2.602365 |
| O | 2.598464 | -0.488442 | -1.430608 | H | -4.35077 | 2.450831 | 1.549781 |
| C | 7.748713 | 3.316263 | 0.346926 | H | -1.664949 | -4.802939 | -0.876027 |
| C | 7.832511 | 4.554556 | 1.245617 | H | -0.267779 | -4.265065 | -1.847782 |
| C | -5.69011 | 1.043598 | 1.904022 | H | -1.791392 | -3.349992 | -1.902798 |

**Table S38.** The Cartesian coordinates of the lowest energy conformers (Conf. A) for **9**.

| **Conf. A** | **X axis** | **Y axis** | **Z axis** | **Conf. A** | **X axis** | **Y axis** | **Z axis** |
| --- | --- | --- | --- | --- | --- | --- | --- |
| C | -1.626468 | 1.618327 | -0.339299 | H | -0.751229 | 0.004384 | -1.507998 |
| C | -1.162308 | 2.883477 | 0.420104 | H | -0.204834 | 1.589603 | -1.988603 |
| C | 0.012941 | 2.511723 | 1.346822 | H | 1.994016 | 0.375723 | 2.409024 |
| C | 0.97648 | 1.534808 | 0.885878 | H | 2.500357 | 2.077695 | 2.319849 |
| C | 0.780337 | 0.816117 | -0.26226 | H | 1.674556 | -0.749028 | -1.522184 |
| C | -0.455867 | 0.980312 | -1.107984 | H | -2.530493 | 2.429973 | -2.15302 |
| C | 2.184925 | 1.218441 | 1.727886 | H | -3.552891 | 2.53062 | -0.699024 |
| O | 3.324249 | 0.869437 | 0.902686 | H | -1.793217 | 3.645283 | 2.060075 |
| C | 3.07162 | 0.031478 | -0.129183 | H | 4.052247 | -1.299378 | -1.524359 |
| C | 1.825962 | -0.078169 | -0.684386 | H | -4.490466 | 0.319088 | 0.216975 |
| C | -2.848338 | 1.899526 | -1.249149 | H | -5.524534 | 0.880344 | -1.063574 |
| O | -2.232266 | 3.336312 | 1.241594 | H | -5.343878 | -1.384947 | -2.199989 |
| O | 0.097423 | 3.078062 | 2.443984 | H | -6.197649 | -1.393708 | -0.661369 |
| C | 4.226824 | -0.665326 | -0.659568 | H | -4.64683 | -3.344885 | -0.992531 |
| C | -3.529623 | 0.605317 | -1.675151 | H | -3.279469 | -2.246055 | -1.049605 |
| C | -4.722259 | 0.158003 | -0.844326 | H | -3.739832 | -1.552682 | 1.329202 |
| C | -5.206914 | -1.270136 | -1.118398 | H | -5.189565 | -2.545152 | 1.335825 |
| C | -4.274407 | -2.383907 | -0.609552 | H | -3.462716 | -3.737602 | 2.52218 |
| C | -4.173413 | -2.477002 | 0.920977 | H | -3.778834 | -4.604333 | 1.026351 |
| C | -3.351976 | -3.673729 | 1.430079 | H | -1.7165 | -3.602543 | 0.004415 |
| O | -3.127711 | -0.029584 | -2.640436 | H | -1.437589 | -2.671812 | 1.468367 |
| C | -1.854917 | -3.617807 | 1.093418 | H | -1.151235 | -4.815 | 2.777805 |
| C | -1.067307 | -4.792321 | 1.684022 | H | -1.442595 | -5.750804 | 1.30405 |
| C | 5.473239 | -0.561 | -0.160218 | H | -0.002361 | -4.728269 | 1.432039 |
| C | 6.590557 | -1.296041 | -0.784431 | H | 5.69233 | 0.055953 | 0.703626 |
| O | 6.508718 | -2.03038 | -1.754749 | H | 8.447817 | -1.575048 | -0.599678 |
| O | 7.756481 | -1.061243 | -0.139887 | H | -0.36942 | 4.874479 | 0.123136 |
| C | -0.703817 | 4.036662 | -0.497294 | H | 0.120841 | 3.750915 | -1.158175 |
| H | -1.940302 | 0.912949 | 0.443444 | H | -1.542512 | 4.380894 | -1.109708 |

**Table S39.** The Cartesian coordinates of the lowest energy conformers (Conf. B) for **9**.

| **Conf. A** | **X axis** | **Y axis** | **Z axis** | **Conf. A** | **X axis** | **Y axis** | **Z axis** |
| --- | --- | --- | --- | --- | --- | --- | --- |
| C | -1.755052 | -1.876034 | 0.245927 | H | -0.833873 | -0.288738 | 1.411277 |
| C | -1.415556 | -3.301204 | -0.250189 | H | -0.727589 | -1.859926 | 2.165612 |
| C | -0.023159 | -3.285717 | -0.913062 | H | 2.979994 | -3.333551 | -0.125121 |
| C | 1.011052 | -2.459136 | -0.3237 | H | 2.504439 | -2.963829 | -1.799181 |
| C | 0.729056 | -1.539436 | 0.64869 | H | 1.655274 | -0.02714 | 1.949662 |
| C | -0.663445 | -1.347752 | 1.193745 | H | -3.163647 | -2.246068 | 1.87237 |
| C | 2.44257 | -2.619476 | -0.766945 | H | -3.860321 | -2.364262 | 0.238093 |
| O | 3.161259 | -1.363292 | -0.726626 | H | -1.83534 | -4.163794 | -1.907485 |
| C | 2.946213 | -0.594312 | 0.366316 | H | 3.778795 | 1.069904 | 1.469923 |
| C | 1.800154 | -0.692177 | 1.106092 | H | -5.469822 | -0.34972 | -0.043849 |
| C | -3.170214 | -1.799657 | 0.872435 | H | -4.085106 | -0.155702 | -1.084706 |
| O | -2.362389 | -3.672313 | -1.244875 | H | -5.045614 | 2.000167 | 0.889756 |
| O | 0.164146 | -4.009115 | -1.899142 | H | -5.553537 | 1.935472 | -0.794691 |
| C | 3.962836 | 0.404662 | 0.630879 | H | -3.170318 | 2.194335 | -1.532543 |
| C | -3.642405 | -0.357321 | 1.001953 | H | -2.65798 | 2.253803 | 0.150793 |
| C | -4.507934 | 0.180578 | -0.127001 | H | -4.600793 | 4.236713 | -1.153637 |
| C | -4.725562 | 1.696146 | -0.114647 | H | -4.014148 | 4.333763 | 0.499904 |
| C | -3.485467 | 2.503074 | -0.524254 | H | -2.256855 | 4.548204 | -2.010658 |
| C | -3.732109 | 4.018353 | -0.515865 | H | -2.870318 | 5.911954 | -1.088147 |
| C | -2.542707 | 4.866303 | -0.996575 | H | -0.93272 | 3.790033 | -0.015829 |
| O | -3.335306 | 0.321579 | 1.97203 | H | -1.601756 | 5.107912 | 0.933712 |
| C | -1.307391 | 4.819629 | -0.085902 | H | 0.159475 | 5.451155 | -1.573974 |
| C | -0.176552 | 5.734436 | -0.568582 | H | 0.691675 | 5.685225 | 0.098864 |
| C | 5.086715 | 0.55717 | -0.095411 | H | -0.505198 | 6.780376 | -0.612773 |
| C | 6.053961 | 1.619295 | 0.242175 | H | 5.313754 | -0.08569 | -0.938029 |
| O | 5.948345 | 2.4168 | 1.15897 | H | 7.705983 | 2.333333 | -0.324564 |
| O | 7.10984 | 1.61217 | -0.603568 | H | -0.69545 | -4.138681 | 1.665287 |
| C | -1.404323 | -4.369359 | 0.863511 | H | -2.404745 | -4.466423 | 1.295519 |
| H | -1.748961 | -1.248606 | -0.65663 | H | -1.126538 | -5.335228 | 0.429193 |

**Table S40.** The Cartesian coordinates of the lowest energy conformers (Conf. C) for **9**.

| **Conf. A** | **X axis** | **Y axis** | **Z axis** | **Conf. A** | **X axis** | **Y axis** | **Z axis** |
| --- | --- | --- | --- | --- | --- | --- | --- |
| C | -0.78371 | -2.23801 | 0.278913 | H | 0.441287 | -1.585136 | 1.956837 |
| C | 0.082882 | -3.391244 | -0.279974 | H | -0.503294 | -0.305497 | 1.240476 |
| C | 1.164293 | -2.8032 | -1.208901 | H | 2.512602 | -0.323092 | -2.489809 |
| C | 1.766266 | -1.535248 | -0.855146 | H | 3.483679 | -1.772258 | -2.146175 |
| C | 1.285853 | -0.77237 | 0.174572 | H | 1.591474 | 1.130001 | 1.234513 |
| C | 0.091057 | -1.19128 | 0.991474 | H | -2.38835 | -3.626016 | 0.689942 |
| C | 2.889395 | -0.979661 | -1.69136 | H | -1.544164 | -3.061101 | 2.152208 |
| O | 3.813956 | -0.207844 | -0.884967 | H | -0.177172 | -4.500979 | -1.819056 |
| C | 3.253366 | 0.636973 | 0.01109 | H | 3.698925 | 2.361635 | 1.238609 |
| C | 1.992627 | 0.437367 | 0.503616 | H | -3.836253 | -1.879173 | -0.553299 |
| C | -1.935352 | -2.756434 | 1.175816 | H | -4.765624 | -2.590938 | 0.738077 |
| O | -0.745463 | -4.240147 | -1.06596 | H | -5.314402 | -0.233556 | 1.585729 |
| O | 1.489931 | -3.44842 | -2.213527 | H | -6.026775 | -0.666484 | 0.034962 |
| C | 4.113335 | 1.706869 | 0.477131 | H | -3.490416 | 1.019569 | 0.390147 |
| C | -2.988082 | -1.67953 | 1.404242 | H | -4.20459 | 0.579361 | -1.157661 |
| C | -4.188976 | -1.691244 | 0.471041 | H | -5.58782 | 2.254305 | 1.010531 |
| C | -5.075542 | -0.444454 | 0.53599 | H | -6.307618 | 1.811581 | -0.53135 |
| C | -4.444481 | 0.798519 | -0.106451 | H | -4.490547 | 3.064702 | -1.739582 |
| C | -5.348523 | 2.036054 | -0.040924 | H | -3.769435 | 3.505952 | -0.19798 |
| C | -4.72907 | 3.281643 | -0.687473 | H | -6.59002 | 4.296733 | -1.109174 |
| O | -2.862116 | -0.85496 | 2.298865 | H | -5.868787 | 4.738215 | 0.430894 |
| C | -5.631125 | 4.520769 | -0.620257 | H | -5.673709 | 6.626717 | -1.204136 |
| C | -5.005415 | 5.760146 | -1.268555 | H | -4.061679 | 6.029507 | -0.777985 |
| C | 5.369028 | 1.919224 | 0.03903 | H | -4.788049 | 5.584617 | -2.329588 |
| C | 6.174123 | 3.02593 | 0.591607 | H | 5.821478 | 1.294663 | -0.722575 |
| O | 5.810985 | 3.815138 | 1.447663 | H | 7.876712 | 3.823581 | 0.431546 |
| O | 7.401256 | 3.074204 | 0.024458 | H | 0.043381 | -4.732026 | 1.43137 |
| C | 0.788173 | -4.232677 | 0.804606 | H | 1.444005 | -3.634651 | 1.445461 |
| H | -1.229622 | -1.759578 | -0.604739 | H | 1.395666 | -5.00328 | 0.319016 |

**Table S41.** The Cartesian coordinates of the lowest energy conformers (Conf. D) for **9**.

| **Conf. A** | **X axis** | **Y axis** | **Z axis** | **Conf. A** | **X axis** | **Y axis** | **Z axis** |
| --- | --- | --- | --- | --- | --- | --- | --- |
| C | 1.430597 | -1.661859 | -0.335412 | H | 0.560143 | -0.177743 | -1.668496 |
| C | 0.956717 | -2.796818 | 0.602897 | H | -0.064921 | -1.78768 | -1.913616 |
| C | -0.15989 | -2.258616 | 1.520557 | H | -2.008257 | 0.075786 | 2.377093 |
| C | -1.108353 | -1.311006 | 0.975128 | H | -2.583554 | -1.599543 | 2.528647 |
| C | -0.939507 | -0.756145 | -0.264476 | H | -1.832099 | 0.670086 | -1.680545 |
| C | 0.249031 | -1.080508 | -1.131881 | H | 2.220526 | -2.750281 | -2.054906 |
| C | -2.263296 | -0.837119 | 1.818396 | H | 3.308658 | -2.684588 | -0.647496 |
| O | -3.425801 | -0.545461 | 1.003302 | H | 1.635574 | -3.363093 | 2.301787 |
| C | -3.189943 | 0.140462 | -0.138937 | H | -4.181591 | 1.326133 | -1.652104 |
| C | -1.969154 | 0.120935 | -0.756148 | H | 5.308294 | -1.172551 | -1.322443 |
| C | 2.597638 | -2.110882 | -1.249781 | H | 4.352096 | -0.394418 | -0.09575 |
| O | 2.046647 | -3.183507 | 1.43178 | H | 6.072497 | 1.105112 | -1.30251 |
| O | -0.213083 | -2.672576 | 2.685659 | H | 5.123631 | 0.913286 | -2.771976 |
| C | -4.340368 | 0.81586 | -0.706146 | H | 4.554303 | 3.046381 | -1.832843 |
| C | 3.296262 | -0.91524 | -1.88475 | H | 3.156732 | 2.006048 | -1.62186 |
| C | 4.539364 | -0.397127 | -1.177918 | H | 3.805896 | 1.640141 | 0.787678 |
| C | 5.051746 | 0.957647 | -1.679044 | H | 5.229436 | 2.64443 | 0.553455 |
| C | 4.188445 | 2.168254 | -1.282218 | H | 2.32574 | 3.591622 | 0.24443 |
| C | 4.191609 | 2.495821 | 0.218125 | H | 3.743802 | 4.586827 | -0.051296 |
| C | 3.369954 | 3.748864 | 0.554511 | H | 4.442896 | 4.315599 | 2.346309 |
| O | 2.869193 | -0.410034 | -2.913666 | H | 2.903555 | 5.134675 | 2.138725 |
| C | 3.399497 | 4.159905 | 2.036159 | H | 3.237163 | 2.190279 | 2.986252 |
| C | 2.72863 | 3.160989 | 2.987086 | H | 2.734858 | 3.534152 | 4.017849 |
| C | -5.564876 | 0.832841 | -0.14574 | H | 1.682966 | 2.988202 | 2.701906 |
| C | -6.680644 | 1.532214 | -0.812207 | H | -5.767074 | 0.341356 | 0.798931 |
| O | -6.616023 | 2.13323 | -1.871497 | H | -8.51502 | 1.914359 | -0.588162 |
| O | -7.823241 | 1.433356 | -0.094818 | H | 0.076822 | -4.773538 | 0.60382 |
| C | 0.414194 | -4.039072 | -0.134739 | H | -0.428076 | -3.806503 | -0.794254 |
| H | 1.806707 | -0.874692 | 0.333584 | H | 1.210847 | -4.496298 | -0.72906 |

**Table S42.** The Cartesian coordinates of the lowest energy conformers (Conf. E) for **9**.

| **Conf. A** | **X axis** | **Y axis** | **Z axis** | **Conf. A** | **X axis** | **Y axis** | **Z axis** |
| --- | --- | --- | --- | --- | --- | --- | --- |
| C | 0.643515 | -0.023448 | 0.496598 | H | -0.851146 | -1.012456 | 1.740593 |
| C | 0.47197 | 1.447319 | 0.937869 | H | -0.583401 | -1.782838 | 0.190617 |
| C | -0.694167 | 2.079166 | 0.153709 | H | -3.158621 | 2.932488 | -0.566871 |
| C | -1.854806 | 1.263085 | -0.135528 | H | -2.993279 | 1.776417 | -1.907456 |
| C | -1.867973 | -0.08261 | 0.113178 | H | -3.122719 | -1.881281 | -0.056764 |
| C | -0.670709 | -0.804676 | 0.675206 | H | 2.671331 | -0.00734 | 1.258116 |
| C | -3.052473 | 1.875886 | -0.813278 | H | 1.592733 | -0.990604 | 2.219323 |
| O | -4.282956 | 1.244788 | -0.378948 | H | 1.33399 | 3.039893 | 0.311213 |
| C | -4.258193 | -0.106224 | -0.30242 | H | -5.557516 | -1.832822 | -0.207472 |
| C | -3.091834 | -0.800262 | -0.131236 | H | 3.417596 | -2.700657 | 2.17525 |
| C | 1.830369 | -0.70964 | 1.183773 | H | 3.563951 | -3.679947 | 0.699162 |
| O | 1.656745 | 2.162753 | 0.601707 | H | 5.674007 | -2.481764 | 1.303166 |
| O | -0.604929 | 3.267632 | -0.18066 | H | 4.863011 | -0.931373 | 1.175735 |
| C | -5.555569 | -0.752377 | -0.321374 | H | 5.171841 | -2.94049 | -1.124712 |
| C | 2.337839 | -1.957246 | 0.471041 | H | 4.453864 | -1.34603 | -1.277067 |
| C | 3.541397 | -2.65546 | 1.086216 | H | 6.81393 | -1.485376 | -2.118025 |
| C | 4.880407 | -1.947838 | 0.764046 | H | 7.373981 | -1.895251 | -0.503869 |
| C | 5.220608 | -1.914944 | -0.732765 | H | 6.610401 | 0.344984 | 0.337843 |
| C | 6.605965 | -1.327531 | -1.050287 | H | 5.966983 | 0.72899 | -1.252568 |
| C | 6.760336 | 0.167497 | -0.736242 | H | 8.919569 | 0.166536 | -0.635622 |
| O | 1.825698 | -2.369653 | -0.560457 | H | 8.278794 | 0.566552 | -2.221908 |
| C | 8.126952 | 0.732495 | -1.145759 | H | 9.267642 | 2.596808 | -1.13704 |
| C | 8.282855 | 2.224135 | -0.832073 | H | 7.524098 | 2.819759 | -1.354848 |
| C | -6.727575 | -0.102341 | -0.455098 | H | 8.171465 | 2.416393 | 0.24238 |
| C | -8.000299 | -0.849035 | -0.441494 | H | -6.772863 | 0.973828 | -0.576055 |
| O | -8.118953 | -2.05677 | -0.318865 | H | -9.862944 | -0.575771 | -0.57064 |
| O | -9.059886 | -0.02063 | -0.587304 | H | 1.033832 | 1.264379 | 3.031161 |
| C | 0.18418 | 1.626361 | 2.444473 | H | -0.717826 | 1.09747 | 2.76917 |
| H | 0.863493 | 0.009008 | -0.57801 | H | 0.05128 | 2.69167 | 2.660099 |

**Table S43.** The Cartesian coordinates of the lowest energy conformers (Conf. F) for **9**.

| **Conf. A** | **X axis** | **Y axis** | **Z axis** | **Conf. A** | **X axis** | **Y axis** | **Z axis** |
| --- | --- | --- | --- | --- | --- | --- | --- |
| C | -1.20504 | -1.984406 | 0.353592 | H | 0.029328 | -1.284733 | 2.006074 |
| C | -0.477237 | -3.298272 | -0.017076 | H | -0.7082 | 0.00313 | 1.089134 |
| C | 0.70548 | -2.97142 | -0.951105 | H | 3.174971 | -2.367301 | -1.878411 |
| C | 1.456731 | -1.756417 | -0.71739 | H | 2.419592 | -0.858722 | -2.439378 |
| C | 1.043385 | -0.81756 | 0.188579 | H | 1.560114 | 1.144729 | 1.035847 |
| C | -0.227649 | -0.975489 | 0.98234 | H | -2.993798 | -3.09568 | 0.837489 |
| C | 2.674867 | -1.455696 | -1.551096 | H | -2.138303 | -2.468228 | 2.267282 |
| O | 3.66287 | -0.715899 | -0.791285 | H | -0.823503 | -4.54937 | -1.425099 |
| C | 3.186695 | 0.29688 | -0.03073 | H | 3.813263 | 2.086948 | 1.010651 |
| C | 1.892644 | 0.322525 | 0.41271 | H | -5.210054 | -1.765822 | 0.627928 |
| C | -2.448159 | -2.237917 | 1.242514 | H | -4.140456 | -1.338364 | -0.680435 |
| O | -1.379957 | -4.128228 | -0.738675 | H | -6.170694 | 0.204577 | -0.36515 |
| O | 0.979414 | -3.771802 | -1.85438 | H | -5.48019 | 0.724697 | 1.16851 |
| C | 4.16408 | 1.296147 | 0.353451 | H | -4.142041 | 1.060117 | -1.573628 |
| C | -3.35647 | -1.015938 | 1.287314 | H | -3.455953 | 1.583992 | -0.039283 |
| C | -4.50777 | -0.985641 | 0.294099 | H | -5.385607 | 3.150988 | 0.296249 |
| C | -5.22263 | 0.362368 | 0.165486 | H | -6.119913 | 2.579149 | -1.189535 |
| C | -4.403075 | 1.431392 | -0.570952 | H | -5.066062 | 4.706592 | -1.644339 |
| C | -5.150284 | 2.765394 | -0.705644 | H | -4.219132 | 3.447084 | -2.529675 |
| C | -4.403599 | 3.837913 | -1.518505 | H | -2.609097 | 5.019477 | -1.641288 |
| O | -3.159315 | -0.113469 | 2.089102 | H | -2.370069 | 3.479914 | -0.839417 |
| C | -3.06824 | 4.323183 | -0.926407 | H | -3.886775 | 5.872154 | 0.375443 |
| C | -3.196886 | 5.020517 | 0.433205 | H | -3.573614 | 4.341629 | 1.206817 |
| C | 5.452393 | 1.290268 | -0.038988 | H | -2.226662 | 5.400429 | 0.773934 |
| C | 6.37673 | 2.343915 | 0.42327 | H | 5.845306 | 0.524489 | -0.697807 |
| O | 6.090259 | 3.27131 | 1.161786 | H | 8.17477 | 2.892736 | 0.26128 |
| O | 7.619515 | 2.165832 | -0.080282 | H | 0.774194 | -3.491969 | 1.795575 |
| C | 0.071007 | -4.078987 | 1.196064 | H | 0.590974 | -4.973886 | 0.83901 |
| H | -1.550782 | -1.567788 | -0.603186 | H | -0.755717 | -4.399453 | 1.836893 |

**Table S44.** The Cartesian coordinates of the lowest energy conformers (Conf. G) for **9**.

| **Conf. A** | **X axis** | **Y axis** | **Z axis** | **Conf. A** | **X axis** | **Y axis** | **Z axis** |
| --- | --- | --- | --- | --- | --- | --- | --- |
| C | -0.20902 | -1.68395 | 0.444136 | H | 0.258892 | 0.279463 | 1.256453 |
| C | 0.541571 | -2.935596 | -0.06876 | H | 1.132745 | -1.019717 | 2.025316 |
| C | 1.623516 | -2.496654 | -1.076129 | H | 3.110143 | -0.221155 | -2.569016 |
| C | 2.33744 | -1.261553 | -0.831226 | H | 3.976217 | -1.720157 | -2.164147 |
| C | 1.961622 | -0.397693 | 0.161506 | H | 2.459966 | 1.53736 | 1.07983 |
| C | 0.773145 | -0.664869 | 1.04829 | H | -0.944253 | -2.321174 | 2.39776 |
| C | 3.465773 | -0.854223 | -1.742395 | H | -1.891168 | -2.915688 | 1.013638 |
| O | 4.480902 | -0.111038 | -1.022613 | H | 0.130881 | -4.11815 | -1.518093 |
| C | 4.026397 | 0.832337 | -0.165658 | H | 4.658458 | 2.588996 | 0.927643 |
| C | 2.774739 | 0.768993 | 0.383111 | H | -4.160748 | -1.770887 | 1.066826 |
| C | -1.355493 | -2.052108 | 1.418836 | H | -3.283289 | -1.089587 | -0.275259 |
| O | -0.382668 | -3.765183 | -0.763215 | H | -4.684614 | 0.555118 | 1.91872 |
| O | 1.85401 | -3.228831 | -2.046935 | H | -3.81058 | 1.245481 | 0.563993 |
| C | 4.988031 | 1.854197 | 0.198358 | H | -5.418233 | 0.145022 | -1.036602 |
| C | -2.316152 | -0.88562 | 1.615501 | H | -6.294579 | -0.54563 | 0.321777 |
| C | -3.575042 | -0.8875 | 0.766467 | H | -6.818955 | 1.768584 | 1.159371 |
| C | -4.418903 | 0.383294 | 0.867956 | H | -5.944382 | 2.458505 | -0.20102 |
| C | -5.691537 | 0.321587 | 0.014273 | H | -8.42567 | 0.66579 | -0.432589 |
| C | -6.547127 | 1.591604 | 0.108115 | H | -7.551231 | 1.35524 | -1.793144 |
| C | -7.822814 | 1.532872 | -0.741709 | H | -8.952204 | 2.979192 | 0.400392 |
| O | -2.066391 | 0.010225 | 2.410014 | H | -8.078918 | 3.667992 | -0.959734 |
| C | -8.680607 | 2.801545 | -0.650018 | H | -10.542775 | 3.653436 | -1.414415 |
| C | -9.952837 | 2.73358 | -1.501275 | H | -9.711908 | 2.590634 | -2.562099 |
| C | 6.23899 | 1.930838 | -0.294635 | H | -10.591746 | 1.897023 | -1.191761 |
| C | 7.153912 | 2.997812 | 0.155428 | H | 6.608279 | 1.222288 | -1.027038 |
| O | 6.890893 | 3.868691 | 0.967766 | H | 8.909685 | 3.634551 | -0.113327 |
| O | 8.358277 | 2.904044 | -0.453391 | H | 1.951764 | -3.17367 | 1.617455 |
| C | 1.227107 | -3.758752 | 1.042138 | H | 1.754673 | -4.603039 | 0.58654 |
| H | -0.656918 | -1.231184 | -0.451888 | H | 0.474019 | -4.158757 | 1.727537 |

**Table S45.** The Cartesian coordinates of the lowest energy conformers (Conf. A) for **10a**.

| **Conf. A** | **X axis** | **Y axis** | **Z axis** | **Conf. A** | **X axis** | **Y axis** | **Z axis** |
| --- | --- | --- | --- | --- | --- | --- | --- |
| C | 1.720156 | -0.967737 | 0.310042 | H | 1.548005 | -0.585015 | -0.704347 |
| C | 3.081859 | -1.665476 | 0.28073 | H | 1.922177 | -0.043562 | 2.275721 |
| C | 4.058031 | -0.72768 | -0.422436 | H | 0.842944 | 0.805714 | 1.18449 |
| C | 3.954884 | 0.704725 | -0.005739 | H | 5.744073 | 1.888013 | 0.310252 |
| C | 2.92666 | 1.137868 | 0.775906 | H | 4.659001 | 2.52755 | -0.936471 |
| C | 1.780094 | 0.244369 | 1.22425 | H | 5.64647 | 1.087335 | -1.26295 |
| C | 5.061527 | 1.609367 | -0.503372 | H | 2.406541 | 2.626913 | 2.233697 |
| O | 2.353034 | 3.722088 | -0.84156 | H | 3.808495 | 3.058423 | 1.237069 |
| C | 1.936922 | 3.397622 | 0.255359 | H | -1.334191 | -3.902211 | 0.011378 |
| C | 2.824039 | 2.58466 | 1.222181 | H | -1.722273 | -2.952168 | 1.422134 |
| C | 0.753964 | -2.136444 | 0.475942 | H | -3.048281 | -2.529787 | -1.32548 |
| C | 1.510276 | -3.234081 | -0.319157 | H | -3.724715 | -3.544575 | -0.05812 |
| O | 2.807654 | -2.871771 | -0.489422 | H | -3.863084 | -1.550218 | 1.468077 |
| O | 4.885167 | -1.138822 | -1.223881 | H | -3.211753 | -0.529623 | 0.188254 |
| O | 1.066935 | -4.265383 | -0.761976 | H | -5.178752 | -1.109945 | -1.269339 |
| C | -0.655819 | -1.923507 | -0.093257 | H | -5.841656 | -2.098213 | 0.02558 |
| C | -1.712516 | -2.916894 | 0.322689 | H | -5.969458 | -0.064171 | 1.506021 |
| C | -3.11391 | -2.650419 | -0.236952 | H | -5.302933 | 0.918575 | 0.208991 |
| C | -3.812922 | -1.427755 | 0.375799 | H | -7.284014 | 0.355933 | -1.233651 |
| C | -5.227724 | -1.207338 | -0.174564 | H | -7.949388 | -0.627093 | 0.061755 |
| C | -5.920073 | 0.030293 | 0.410733 | H | -9.022484 | 1.637233 | 0.0364 |
| O | -0.885128 | -1.005702 | -0.868818 | H | -8.111524 | 1.418564 | 1.539526 |
| C | -7.333331 | 0.261006 | -0.139497 | H | -7.440453 | 2.408585 | 0.234686 |
| C | -8.016397 | 1.499758 | 0.449451 | H | 0.692754 | 4.812647 | 1.194462 |
| C | 0.554064 | 3.819932 | 0.741195 | H | 0.214851 | 3.16956 | 1.553354 |
| C | -0.50038 | 3.920658 | -0.375881 | H | -1.323153 | 4.571687 | -0.075796 |
| C | -1.091204 | 2.570926 | -0.733369 | H | -0.029204 | 4.348931 | -1.268119 |
| O | -0.131847 | 1.668131 | -1.013645 | H | -0.530651 | 0.77283 | -1.125592 |
| O | -2.279007 | 2.310452 | -0.754693 | H | 2.963112 | -2.709863 | 2.199277 |
| C | 3.67301 | -2.103191 | 1.628811 | H | 4.565776 | -2.70603 | 1.438035 |
| H | 0.666556 | -2.487768 | 1.511814 | H | 3.965493 | -1.244173 | 2.237669 |

**Table S46.** The Cartesian coordinates of the lowest energy conformers (Conf. B) for **10a**.

| **Conf. A** | **X axis** | **Y axis** | **Z axis** | **Conf. A** | **X axis** | **Y axis** | **Z axis** |
| --- | --- | --- | --- | --- | --- | --- | --- |
| C | 1.255466 | -1.017651 | 0.387958 | H | 1.320719 | -0.631347 | -0.638272 |
| C | 2.459775 | -1.945842 | 0.577282 | H | 1.240627 | -0.113846 | 2.372576 |
| C | 3.683451 | -1.198842 | 0.067643 | H | 0.589082 | 0.914414 | 1.114699 |
| C | 3.792436 | 0.216201 | 0.543869 | H | 5.94098 | 0.162567 | 0.619026 |
| C | 2.740307 | 0.836099 | 1.148519 | H | 5.280975 | 1.801793 | 0.743036 |
| C | 1.373821 | 0.178041 | 1.32081 | H | 5.294177 | 0.952397 | -0.808797 |
| C | 5.144914 | 0.828801 | 0.270465 | H | 2.340403 | 2.283091 | 2.682661 |
| O | 0.947397 | 3.703536 | 1.190169 | H | 3.824697 | 2.61515 | 1.768295 |
| C | 1.99933 | 3.225706 | 0.80474 | H | -2.104135 | -3.461197 | -0.472439 |
| C | 2.796302 | 2.253868 | 1.687476 | H | -2.550517 | -2.571903 | 0.959081 |
| C | 0.092986 | -2.008844 | 0.398377 | H | -3.456125 | -1.751745 | -1.866274 |
| C | 0.764179 | -3.227342 | -0.291595 | H | -4.392989 | -2.814732 | -0.824199 |
| O | 2.114378 | -3.093865 | -0.248026 | H | -4.546082 | -1.007128 | 0.901525 |
| O | 4.539616 | -1.729725 | -0.623714 | H | -3.623662 | 0.07423 | -0.136625 |
| O | 0.229984 | -4.171947 | -0.819129 | H | -5.392937 | -0.132313 | -1.914993 |
| C | -1.185362 | -1.587452 | -0.335372 | H | -6.345224 | -1.151454 | -0.846148 |
| C | -2.399518 | -2.46017 | -0.124473 | H | -5.558173 | 1.761082 | -0.266796 |
| C | -3.679331 | -1.981007 | -0.81692 | H | -7.102159 | 1.246084 | -0.92863 |
| C | -4.333651 | -0.763258 | -0.148413 | H | -7.546971 | -0.088011 | 1.160766 |
| C | -5.622296 | -0.321831 | -0.856677 | H | -6.037769 | 0.492851 | 1.8476 |
| C | -6.283727 | 0.93366 | -0.264157 | H | -7.952287 | 1.864152 | 2.682081 |
| O | -1.197614 | -0.611781 | -1.072384 | H | -6.873439 | 2.871642 | 1.703521 |
| C | -6.846021 | 0.759349 | 1.153933 | H | -8.398616 | 2.28815 | 1.02093 |
| C | -7.557688 | 2.014532 | 1.670456 | H | 3.004453 | 4.563192 | -0.50451 |
| C | 2.562475 | 3.560524 | -0.574574 | H | 3.366356 | 2.872214 | -0.84654 |
| C | 1.473818 | 3.564859 | -1.669309 | H | 0.656668 | 4.229226 | -1.384573 |
| C | 0.960966 | 2.154223 | -1.884484 | H | 1.914158 | 3.917654 | -2.606349 |
| O | -0.289558 | 1.980582 | -1.431872 | H | -0.52007 | 1.021276 | -1.485975 |
| O | 1.633442 | 1.252088 | -2.355214 | H | 1.8462 | -2.935388 | 2.431485 |
| C | 2.733151 | -2.463736 | 1.998176 | H | 3.529333 | -3.21241 | 1.947864 |
| H | -0.18462 | -2.328441 | 1.411227 | H | 3.061095 | -1.661694 | 2.663734 |

**Table S47.** The Cartesian coordinates of the lowest energy conformers (Conf. C) for **10a**.

| **Conf. A** | **X axis** | **Y axis** | **Z axis** | **Conf. A** | **X axis** | **Y axis** | **Z axis** |
| --- | --- | --- | --- | --- | --- | --- | --- |
| C | 1.620318 | -0.97743 | 0.310107 | H | 1.444512 | -0.594221 | -0.703583 |
| C | 2.987194 | -1.665861 | 0.278653 | H | 1.819966 | -0.056237 | 2.277315 |
| C | 3.958208 | -0.721762 | -0.422536 | H | 0.735303 | 0.790577 | 1.18924 |
| C | 3.848651 | 0.708313 | -0.001246 | H | 5.630417 | 1.89985 | 0.323417 |
| C | 2.81704 | 1.133997 | 0.78002 | H | 4.547457 | 2.535963 | -0.92677 |
| C | 1.674974 | 0.233577 | 1.226739 | H | 5.543157 | 1.10093 | -1.251432 |
| C | 4.952657 | 1.61912 | -0.493525 | H | 2.278992 | 2.620044 | 2.23506 |
| O | 2.253238 | 3.713972 | -0.841635 | H | 3.690124 | 3.055445 | 1.253242 |
| C | 1.828268 | 3.391982 | 0.252716 | H | -1.34846 | -3.964606 | 0.02388 |
| C | 2.706701 | 2.579747 | 1.227854 | H | -1.805282 | -3.023713 | 1.419513 |
| C | 0.664277 | -2.154741 | 0.477076 | H | -3.103806 | -2.682769 | -1.352555 |
| C | 1.427837 | -3.245358 | -0.322096 | H | -3.739896 | -3.732411 | -0.093794 |
| O | 2.721606 | -2.872338 | -0.49356 | H | -3.978772 | -1.719788 | 1.428725 |
| O | 4.786671 | -1.126977 | -1.22572 | H | -3.443097 | -0.690426 | 0.103802 |
| O | 0.99373 | -4.280113 | -0.766146 | H | -5.406734 | -1.487495 | -1.277957 |
| C | -0.754336 | -1.958893 | -0.076894 | H | -5.904437 | -2.52051 | 0.053342 |
| C | -1.776195 | -2.995402 | 0.319779 | H | -7.307328 | -0.587357 | 0.152093 |
| C | -3.17977 | -2.803854 | -0.264573 | H | -6.193325 | -0.450219 | 1.50334 |
| C | -3.965022 | -1.626263 | 0.332235 | H | -4.801215 | 1.184748 | 0.152627 |
| C | -5.411488 | -1.565946 | -0.180366 | H | -5.927099 | 1.011034 | -1.18094 |
| C | -6.25075 | -0.41738 | 0.404614 | H | -6.431325 | 3.079154 | 0.139867 |
| O | -1.015956 | -1.023167 | -0.820259 | H | -7.784702 | 1.942504 | 0.260655 |
| C | -5.853332 | 0.982924 | -0.084014 | H | -6.648733 | 2.111619 | 1.607158 |
| C | -6.727141 | 2.09162 | 0.512772 | H | 0.576171 | 4.811668 | 1.174546 |
| C | 0.441763 | 3.816614 | 0.725202 | H | 0.095307 | 3.169656 | 1.536953 |
| C | -0.600903 | 3.91113 | -0.402845 | H | -1.426337 | 4.565298 | -0.117095 |
| C | -1.190483 | 2.561112 | -0.760947 | H | -0.120207 | 4.333662 | -1.29294 |
| O | -0.231328 | 1.643009 | -0.986623 | H | -0.634894 | 0.749235 | -1.096598 |
| O | -2.379005 | 2.313414 | -0.829524 | H | 2.876384 | -2.714502 | 2.1959 |
| C | 3.581755 | -2.102134 | 1.625947 | H | 4.478282 | -2.698816 | 1.433468 |
| H | 0.5875 | -2.509036 | 1.513092 | H | 3.869073 | -1.242353 | 2.236133 |

**Table S48.** The Cartesian coordinates of the lowest energy conformers (Conf. D) for **10a**.

| **Conf. A** | **X axis** | **Y axis** | **Z axis** | **Conf. A** | **X axis** | **Y axis** | **Z axis** |
| --- | --- | --- | --- | --- | --- | --- | --- |
| C | 1.272489 | -1.030661 | 0.326926 | H | 1.158962 | -0.416385 | -0.576556 |
| C | 2.061413 | -2.274894 | -0.094294 | H | 2.180216 | -0.728772 | 2.286729 |
| C | 3.254793 | -1.791539 | -0.905288 | H | 1.59492 | 0.740075 | 1.536897 |
| C | 4.002349 | -0.648731 | -0.291797 | H | 5.899033 | -1.323279 | -1.048292 |
| C | 3.478828 | 0.052458 | 0.752748 | H | 5.949769 | 0.331265 | -0.417579 |
| C | 2.086852 | -0.214495 | 1.319376 | H | 5.183371 | -0.014761 | -1.973984 |
| C | 5.333768 | -0.390203 | -0.954711 | H | 4.118709 | 1.063713 | 2.53378 |
| O | 2.944788 | 3.174499 | 1.943069 | H | 5.264028 | 1.21432 | 1.186699 |
| C | 3.580293 | 2.552082 | 1.111047 | H | -2.791096 | -2.17089 | 0.534294 |
| C | 4.203277 | 1.189931 | 1.449312 | H | -2.492695 | -1.406657 | 2.075015 |
| C | -0.106867 | -1.618673 | 0.618906 | H | -3.609702 | 0.713625 | 1.258928 |
| C | -0.157733 | -2.746727 | -0.44678 | H | -3.905782 | -0.046789 | -0.294852 |
| O | 1.095762 | -3.01301 | -0.894844 | H | -5.338698 | -1.787437 | 0.820732 |
| O | 3.611178 | -2.331896 | -1.941626 | H | -4.985302 | -1.077996 | 2.389185 |
| O | -1.126709 | -3.328148 | -0.869846 | H | -7.183333 | -0.348666 | 1.734009 |
| C | -1.307317 | -0.674661 | 0.486711 | H | -6.122377 | 1.051275 | 1.69367 |
| C | -2.627377 | -1.190181 | 1.003823 | H | -5.969403 | 0.871943 | -0.807427 |
| C | -3.813983 | -0.251681 | 0.777996 | H | -6.959991 | -0.580053 | -0.76878 |
| C | -5.124161 | -0.834359 | 1.326337 | H | -8.86038 | 0.747443 | 0.199001 |
| C | -6.339408 | 0.097837 | 1.18928 | H | -7.865205 | 2.195193 | 0.176293 |
| C | -6.77977 | 0.376402 | -0.254892 | H | -9.387162 | 2.143206 | -1.813568 |
| O | -1.191375 | 0.432729 | -0.018374 | H | -7.699233 | 2.05144 | -2.342139 |
| C | -8.04292 | 1.243092 | -0.343989 | H | -8.699954 | 0.591615 | -2.320878 |
| C | -8.483128 | 1.523866 | -1.784498 | H | 4.544626 | 3.872667 | -0.244908 |
| C | 3.774174 | 3.092565 | -0.303825 | H | 4.152832 | 2.314681 | -0.971354 |
| C | 2.480059 | 3.704261 | -0.882014 | H | 2.082477 | 4.45757 | -0.2003 |
| C | 1.458849 | 2.608179 | -1.116675 | H | 2.707915 | 4.173902 | -1.843275 |
| O | 0.424771 | 2.669266 | -0.264907 | H | -0.136264 | 1.864378 | -0.382953 |
| O | 1.592476 | 1.722724 | -1.944831 | H | 1.724428 | -3.517422 | 1.676047 |
| C | 2.547105 | -3.212249 | 1.022548 | H | 2.97066 | -4.110296 | 0.563068 |
| H | -0.164246 | -2.103921 | 1.602097 | H | 3.322234 | -2.744006 | 1.633791 |

**Table S49.** The Cartesian coordinates of the lowest energy conformers (Conf. E) for **10a**.

| **Conf. A** | **X axis** | **Y axis** | **Z axis** | **Conf. A** | **X axis** | **Y axis** | **Z axis** |
| --- | --- | --- | --- | --- | --- | --- | --- |
| C | 1.059301 | -1.151562 | 0.404778 | H | 0.774069 | -0.502706 | -0.434252 |
| C | 1.928205 | -2.266027 | -0.187374 | H | 2.150629 | -0.863572 | 2.269319 |
| C | 2.960189 | -1.598808 | -1.084391 | H | 1.320077 | 0.568452 | 1.700313 |
| C | 3.639838 | -0.411748 | -0.476082 | H | 5.509236 | -0.807423 | -1.463023 |
| C | 3.163673 | 0.161319 | 0.664794 | H | 5.427934 | 0.811659 | -0.749102 |
| C | 1.886982 | -0.305064 | 1.359585 | H | 4.541759 | 0.43793 | -2.23393 |
| C | 4.848493 | 0.04163 | -1.258686 | H | 3.88188 | 1.1494 | 2.42986 |
| O | 2.393913 | 3.116622 | 2.102486 | H | 4.83939 | 1.510017 | 0.97965 |
| C | 3.010664 | 2.629338 | 1.171947 | H | -2.703176 | -2.851166 | 1.15175 |
| C | 3.826294 | 1.340923 | 1.353094 | H | -2.426951 | -1.887771 | 2.584473 |
| C | -0.20162 | -1.916749 | 0.801347 | H | -4.749768 | -1.558335 | 2.031032 |
| C | -0.249859 | -2.976536 | -0.332266 | H | -3.927785 | -0.063911 | 1.601412 |
| O | 0.965151 | -3.064474 | -0.931394 | H | -3.857733 | -0.836795 | -0.813495 |
| O | 3.253972 | -2.033109 | -2.188079 | H | -4.683563 | -2.312496 | -0.34773 |
| O | -1.19166 | -3.641079 | -0.688913 | H | -6.291861 | -0.791067 | -1.336 |
| C | -1.509511 | -1.121745 | 0.863089 | H | -6.687263 | -1.038792 | 0.357529 |
| C | -2.682883 | -1.815463 | 1.51541 | H | -7.023572 | 1.295386 | -0.263076 |
| C | -4.033692 | -1.118608 | 1.324889 | H | -5.759956 | 1.192745 | 0.946932 |
| C | -4.586013 | -1.245565 | -0.10266 | H | -4.01174 | 1.482507 | -0.810744 |
| C | -5.952665 | -0.570905 | -0.313734 | H | -5.245348 | 1.473534 | -2.063042 |
| C | -5.991246 | 0.953237 | -0.100671 | H | -4.539812 | 3.819369 | -1.516931 |
| O | -1.581864 | 0.015496 | 0.419589 | H | -6.244745 | 3.583707 | -1.097006 |
| C | -5.055242 | 1.754365 | -1.016793 | H | -5.012346 | 3.583797 | 0.173323 |
| C | -5.221137 | 3.269638 | -0.857117 | H | 3.659784 | 4.133522 | -0.181046 |
| C | 2.985195 | 3.267838 | -0.214754 | H | 3.378328 | 2.581512 | -0.968639 |
| C | 1.572336 | 3.743288 | -0.615404 | H | 1.166371 | 4.406866 | 0.14942 |
| C | 0.667298 | 2.542338 | -0.810561 | H | 1.635174 | 4.284813 | -1.563869 |
| O | -0.282948 | 2.446956 | 0.130773 | H | -0.753879 | 1.58503 | 0.024108 |
| O | 0.824788 | 1.710916 | -1.68911 | H | 1.933314 | -3.639715 | 1.515708 |
| C | 2.638294 | -3.203678 | 0.801689 | H | 3.102332 | -4.017932 | 0.237237 |
| H | -0.091113 | -2.463049 | 1.747525 | H | 3.422153 | -2.684876 | 1.358689 |

**Table S50.** The Cartesian coordinates of the lowest energy conformers (Conf. F) for **10a**.

| **Conf. A** | **X axis** | **Y axis** | **Z axis** | **Conf. A** | **X axis** | **Y axis** | **Z axis** |
| --- | --- | --- | --- | --- | --- | --- | --- |
| C | 1.601613 | -0.980654 | 0.332029 | H | 1.522006 | -0.62424 | -0.703193 |
| C | 2.948155 | -1.700476 | 0.435976 | H | 1.653246 | -0.006688 | 2.282769 |
| C | 3.995384 | -0.798132 | -0.209106 | H | 0.686826 | 0.830588 | 1.081653 |
| C | 3.884547 | 0.646463 | 0.161531 | H | 5.664619 | 1.805748 | 0.595604 |
| C | 2.803577 | 1.116853 | 0.844098 | H | 4.696778 | 2.433934 | -0.749473 |
| C | 1.606591 | 0.255046 | 1.215916 | H | 5.679607 | 0.969348 | -0.961893 |
| C | 5.045088 | 1.519503 | -0.264504 | H | 2.194589 | 2.650091 | 2.218996 |
| O | 2.414319 | 3.675532 | -0.871723 | H | 3.680896 | 3.033446 | 1.330486 |
| C | 1.901593 | 3.377618 | 0.191314 | H | -1.476636 | -3.869808 | -0.142761 |
| C | 2.692381 | 2.575853 | 1.246374 | H | -1.956388 | -2.872296 | 1.20554 |
| C | 0.603227 | -2.128103 | 0.447489 | H | -3.048141 | -2.506702 | -1.651263 |
| C | 1.403049 | -3.260525 | -0.249773 | H | -3.847045 | -3.465413 | -0.411977 |
| O | 2.716694 | -2.924467 | -0.320185 | H | -4.049209 | -1.421674 | 1.036791 |
| O | 4.877385 | -1.243475 | -0.929621 | H | -3.285497 | -0.454614 | -0.22239 |
| O | 0.978944 | -4.296752 | -0.699858 | H | -5.132404 | -1.102277 | -1.81565 |
| C | -0.74861 | -1.909126 | -0.246519 | H | -5.940766 | -1.960717 | -0.512348 |
| C | -1.857244 | -2.868626 | 0.110042 | H | -5.317867 | 1.04758 | -0.532793 |
| C | -3.202799 | -2.590674 | -0.568323 | H | -6.866319 | 0.335352 | -0.961375 |
| C | -3.91882 | -1.334019 | -0.050727 | H | -7.070594 | -0.607021 | 1.365531 |
| C | -5.279182 | -1.107716 | -0.726113 | H | -5.559659 | 0.172423 | 1.80871 |
| C | -5.983659 | 0.197658 | -0.320264 | H | -7.499372 | 1.570474 | 2.534351 |
| O | -0.891186 | -1.015578 | -1.069723 | H | -6.563774 | 2.433885 | 1.303081 |
| C | -6.431389 | 0.260805 | 1.147007 | H | -8.090166 | 1.651985 | 0.86621 |
| C | -7.188616 | 1.550114 | 1.483262 | H | 0.597795 | 4.821356 | 0.995359 |
| C | 0.486964 | 3.822554 | 0.547917 | H | 0.071138 | 3.189826 | 1.338212 |
| C | -0.465479 | 3.914476 | -0.658111 | H | -1.300045 | 4.584073 | -0.443798 |
| C | -1.045683 | 2.56584 | -1.035928 | H | 0.088056 | 4.315555 | -1.51488 |
| O | -0.080568 | 1.644604 | -1.220933 | H | -0.483262 | 0.752756 | -1.345404 |
| O | -2.231785 | 2.320941 | -1.146934 | H | 2.653005 | -2.68372 | 2.368006 |
| C | 3.418725 | -2.107343 | 1.84007 | H | 4.311861 | -2.731238 | 1.740581 |
| H | 0.421993 | -2.447387 | 1.481519 | H | 3.677839 | -1.235398 | 2.445675 |

**Table S51.** The Cartesian coordinates of the lowest energy conformers (Conf. G) for **10a**.

| **Conf. A** | **X axis** | **Y axis** | **Z axis** | **Conf. A** | **X axis** | **Y axis** | **Z axis** |
| --- | --- | --- | --- | --- | --- | --- | --- |
| C | 1.06805 | -0.979722 | 0.448543 | H | 1.025288 | -0.38248 | -0.472444 |
| C | 1.62502 | -2.350309 | 0.049004 | H | 2.084356 | -0.776302 | 2.36749 |
| C | 2.854654 | -2.10032 | -0.811392 | H | 1.721865 | 0.747654 | 1.586192 |
| C | 3.800949 | -1.077966 | -0.26384 | H | 5.539179 | -2.080141 | -1.037956 |
| C | 3.43614 | -0.2651 | 0.76712 | H | 5.008837 | -0.715455 | -2.006278 |
| C | 2.041112 | -0.281291 | 1.386463 | H | 5.873987 | -0.42936 | -0.48986 |
| C | 5.132844 | -1.065249 | -0.974493 | H | 5.399968 | 0.604519 | 1.10077 |
| O | 3.456453 | 2.94723 | 1.826821 | H | 4.29144 | 0.698664 | 2.483398 |
| C | 3.953165 | 2.195024 | 1.007745 | H | -3.051396 | -1.507375 | 1.027522 |
| C | 4.358938 | 0.764757 | 1.392458 | H | -2.603181 | -0.488715 | 2.375209 |
| C | -0.377193 | -1.323374 | 0.803002 | H | -4.7056 | 0.369934 | 1.606049 |
| C | -0.651688 | -2.45668 | -0.221862 | H | -3.502349 | 1.540863 | 1.081878 |
| O | 0.523992 | -2.940507 | -0.697934 | H | -3.499418 | 0.483172 | -1.212651 |
| O | 3.08164 | -2.727624 | -1.83505 | H | -4.741632 | -0.63913 | -0.688575 |
| O | -1.718515 | -2.878687 | -0.595454 | H | -4.998205 | 2.418431 | -0.82585 |
| C | -1.415046 | -0.204395 | 0.674023 | H | -5.624151 | 1.293715 | -2.020568 |
| C | -2.769089 | -0.478444 | 1.286073 | H | -6.591092 | 1.509999 | 0.887631 |
| C | -3.869374 | 0.5207 | 0.913648 | H | -7.386435 | 2.182645 | -0.521618 |
| C | -4.357084 | 0.378638 | -0.536277 | H | -7.68298 | -0.141597 | -1.45474 |
| C | -5.422144 | 1.41119 | -0.946345 | H | -6.955203 | -0.809914 | -0.00259 |
| C | -6.758913 | 1.344426 | -0.186094 | H | -9.445583 | -0.888939 | 0.166039 |
| O | -1.145848 | 0.852565 | 0.121624 | H | -8.795824 | 0.201969 | 1.400565 |
| C | -7.542264 | 0.038364 | -0.379042 | H | -9.537786 | 0.865306 | -0.062994 |
| C | -8.906559 | 0.053253 | 0.319086 | H | 4.400881 | 1.779651 | -1.077672 |
| C | 4.180609 | 2.63649 | -0.436254 | H | 5.070811 | 3.279287 | -0.437371 |
| C | 2.984401 | 3.430969 | -1.003339 | H | 2.743203 | 4.267789 | -0.346244 |
| C | 1.787621 | 2.51226 | -1.154753 | H | 3.250162 | 3.815287 | -1.992335 |
| O | 0.814738 | 2.774108 | -0.269484 | H | 0.12325 | 2.071184 | -0.335285 |
| O | 1.738333 | 1.588169 | -1.949316 | H | 2.24011 | -4.289933 | 0.747124 |
| C | 1.984423 | -3.320609 | 1.1853 | H | 2.844484 | -2.96835 | 1.759668 |
| H | -0.478101 | -1.764716 | 1.803183 | H | 1.143356 | -3.465364 | 1.870011 |

**Table S52.** The Cartesian coordinates of the lowest energy conformers (Conf. H) for **10a**.

| **Conf. A** | **X axis** | **Y axis** | **Z axis** | **Conf. A** | **X axis** | **Y axis** | **Z axis** |
| --- | --- | --- | --- | --- | --- | --- | --- |
| C | 1.428924 | -1.072434 | 0.288362 | H | 1.380634 | -0.6549 | -0.726592 |
| C | 2.430186 | -2.229778 | 0.234275 | H | 2.001933 | -0.290484 | 2.239304 |
| C | 3.669787 | -1.724573 | -0.492866 | H | 1.307505 | 0.904773 | 1.170621 |
| C | 4.133842 | -0.368454 | -0.070746 | H | 5.473564 | -0.009249 | -1.708551 |
| C | 3.363469 | 0.422743 | 0.726607 | H | 5.823939 | 0.985036 | -0.295922 |
| C | 1.964674 | 0.02922 | 1.187755 | H | 6.240514 | -0.745895 | -0.309444 |
| C | 5.493792 | -0.002975 | -0.613367 | H | 4.87956 | 1.852337 | 1.324903 |
| O | 3.87883 | 2.817007 | -0.989128 | H | 3.329144 | 2.033705 | 2.146269 |
| C | 3.431135 | 2.879752 | 0.140735 | H | -2.438775 | -2.761008 | 0.134906 |
| C | 3.795216 | 1.801189 | 1.184979 | H | -2.497113 | -1.713636 | 1.529584 |
| C | 0.107155 | -1.804332 | 0.504333 | H | -3.693879 | 0.052547 | 0.166036 |
| C | 0.374393 | -3.102251 | -0.305376 | H | -3.663093 | -1.007431 | -1.232429 |
| O | 1.707449 | -3.245238 | -0.518757 | H | -4.983367 | -2.732854 | 0.047247 |
| O | 4.268825 | -2.403508 | -1.314443 | H | -5.069524 | -1.643548 | 1.423523 |
| O | -0.430971 | -3.896568 | -0.725348 | H | -6.232144 | -1.061078 | -1.358925 |
| C | -1.159713 | -1.089128 | 0.020897 | H | -7.145977 | -1.682624 | 0.006879 |
| C | -2.475755 | -1.702089 | 0.428649 | H | -6.607441 | 0.407469 | 1.311253 |
| C | -3.712413 | -1.000772 | -0.135691 | H | -5.765473 | 1.057384 | -0.088693 |
| C | -5.015058 | -1.669921 | 0.325056 | H | -7.883467 | 0.920036 | -1.431534 |
| C | -6.291909 | -1.04437 | -0.260452 | H | -8.728803 | 0.281603 | -0.029884 |
| C | -6.583325 | 0.387415 | 0.211039 | H | -9.149904 | 2.740201 | -0.264835 |
| O | -1.093639 | -0.075923 | -0.661003 | H | -8.261679 | 2.421971 | 1.234081 |
| C | -7.905656 | 0.944572 | -0.332618 | H | -7.410921 | 3.064944 | -0.178674 |
| C | -8.199876 | 2.372166 | 0.139751 | H | 2.122597 | 3.894641 | 1.561589 |
| C | 2.509001 | 4.028329 | 0.548205 | H | 3.117082 | 4.941764 | 0.545171 |
| C | 1.340357 | 4.210751 | -0.449219 | H | 0.813846 | 5.138925 | -0.211468 |
| C | 0.358745 | 3.063828 | -0.3096 | H | 1.728791 | 4.266856 | -1.467703 |
| O | 0.473804 | 2.155168 | -1.292214 | H | -0.122984 | 1.393046 | -1.088322 |
| O | -0.405879 | 2.937189 | 0.631083 | H | 3.448769 | -3.747755 | 1.365877 |
| C | 2.846881 | -2.858009 | 1.573079 | H | 3.450129 | -2.170152 | 2.170561 |
| H | -0.050509 | -2.105232 | 1.548363 | H | 1.97659 | -3.161677 | 2.162574 |

**Table S53.** The Cartesian coordinates of the lowest energy conformers (Conf. A) for **10b**.

| **Conf. A** | **X axis** | **Y axis** | **Z axis** | **Conf. A** | **X axis** | **Y axis** | **Z axis** |
| --- | --- | --- | --- | --- | --- | --- | --- |
| C | 2.025602 | -0.705548 | 0.629833 | H | 0.438654 | 0.59737 | -0.03534 |
| C | 3.380249 | -1.23294 | 0.121453 | H | 1.085291 | -0.405407 | -1.309135 |
| C | 4.121247 | -0.291739 | -0.86166 | H | 4.988646 | 1.695284 | -2.618247 |
| C | 3.598269 | 1.070768 | -1.103287 | H | 4.195674 | 3.047238 | -1.789943 |
| C | 2.283471 | 1.309183 | -0.88177 | H | 5.473559 | 2.123853 | -0.984258 |
| C | 1.368873 | 0.191163 | -0.428365 | H | 0.716377 | 2.481057 | -1.755052 |
| C | 4.608256 | 2.046756 | -1.65266 | H | 2.211495 | 3.382908 | -1.579421 |
| O | 1.596729 | 2.902101 | 1.290465 | H | 1.592743 | -2.479098 | 1.798555 |
| C | 1.082907 | 3.213667 | 0.228534 | H | -0.875123 | -3.893205 | 1.255249 |
| C | 1.582706 | 2.62565 | -1.097091 | H | -0.942102 | -3.316538 | -0.377661 |
| C | 1.251412 | -2.020393 | 0.856367 | H | -3.218505 | -3.6814 | 0.773836 |
| C | 1.811735 | -2.923218 | -0.246198 | H | -2.849408 | -2.32451 | 1.830922 |
| O | 3.013553 | -2.435756 | -0.644571 | H | -3.076908 | -2.192103 | -1.227876 |
| O | 5.169903 | -0.683299 | -1.360781 | H | -2.685138 | -0.819242 | -0.198796 |
| O | 1.352598 | -3.934122 | -0.724153 | H | -5.299776 | -2.418322 | -0.081639 |
| C | -0.268558 | -1.88782 | 1.049735 | H | -4.899564 | -1.067216 | 0.970303 |
| C | -1.181283 | -3.024999 | 0.651712 | H | -4.729582 | 0.445235 | -1.030143 |
| C | -2.674919 | -2.729408 | 0.826358 | H | -5.133869 | -0.90143 | -2.085735 |
| C | -3.240257 | -1.765281 | -0.227059 | H | -6.955184 | 0.210215 | 0.118385 |
| C | -4.734685 | -1.475186 | -0.038263 | H | -7.357825 | -1.135594 | -0.936895 |
| C | -5.298013 | -0.495747 | -1.076096 | H | -8.410572 | 0.980005 | -1.770289 |
| O | -0.675794 | -0.878408 | 1.60729 | H | -6.818679 | 1.748186 | -1.88424 |
| C | -6.790745 | -0.194853 | -0.890394 | H | -7.224635 | 0.392522 | -2.947335 |
| C | -7.343561 | 0.785761 | -1.929957 | H | -0.566214 | 4.184543 | -0.796504 |
| C | -0.106532 | 4.172349 | 0.194012 | H | 0.26146 | 5.183888 | 0.405203 |
| C | -1.13826 | 3.774264 | 1.267169 | H | -0.719039 | 3.884676 | 2.268718 |
| C | -1.573824 | 2.334637 | 1.060528 | H | -2.018417 | 4.42403 | 1.197176 |
| O | -1.755159 | 1.664991 | 2.206555 | H | -1.847239 | 0.711737 | 1.983715 |
| O | -1.703796 | 1.82112 | -0.039432 | H | 3.892228 | -2.336642 | 1.930361 |
| C | 4.355874 | -1.639094 | 1.226311 | H | 5.23359 | -2.112999 | 0.781323 |
| H | 2.153026 | -0.135114 | 1.552085 | H | 4.680977 | -0.754779 | 1.784415 |

**Table S54.** The Cartesian coordinates of the lowest energy conformers (Conf. B) for **10b**.

| **Conf. A** | **X axis** | **Y axis** | **Z axis** | **Conf. A** | **X axis** | **Y axis** | **Z axis** |
| --- | --- | --- | --- | --- | --- | --- | --- |
| C | 1.874742 | -0.710026 | 0.719396 | H | 1.815932 | -0.114341 | 1.632486 |
| C | 3.289774 | -1.288101 | 0.53199 | H | 0.512142 | 0.605734 | -0.317421 |
| C | 4.256775 | -0.394344 | -0.285803 | H | 1.404493 | -0.446431 | -1.387487 |
| C | 3.843333 | 0.976013 | -0.659794 | H | 5.529642 | 1.547642 | -1.865052 |
| C | 2.520055 | 1.254064 | -0.738502 | H | 4.640244 | 2.927813 | -1.197382 |
| C | 1.495988 | 0.169525 | -0.479689 | H | 5.697014 | 1.953956 | -0.163244 |
| C | 4.979128 | 1.91171 | -0.990187 | H | 1.222224 | 2.447255 | -1.956755 |
| O | 1.418075 | 2.909966 | 1.202472 | H | 2.666709 | 3.313857 | -1.464715 |
| C | 1.158824 | 3.209961 | 0.048492 | H | -1.163373 | -3.812013 | 0.76523 |
| C | 1.923951 | 2.583222 | -1.124162 | H | -0.905851 | -3.255008 | -0.85541 |
| C | 1.027547 | -1.995968 | 0.803161 | H | -3.37198 | -3.570109 | -0.130226 |
| C | 1.783831 | -2.945899 | -0.129433 | H | -3.170581 | -2.181768 | 0.930407 |
| O | 3.058948 | -2.502829 | -0.267799 | H | -2.810073 | -2.182396 | -2.117684 |
| O | 5.375388 | -0.82724 | -0.537681 | H | -2.603787 | -0.761595 | -1.102736 |
| O | 1.40634 | -3.95773 | -0.672338 | H | -4.812763 | -0.861233 | -2.296285 |
| C | -0.493175 | -1.818499 | 0.658798 | H | -5.226384 | -2.326289 | -1.41894 |
| C | -1.333237 | -2.947087 | 0.106578 | H | -5.167455 | -1.059253 | 0.745915 |
| C | -2.827022 | -2.624695 | -0.010951 | H | -4.656959 | 0.391024 | -0.106781 |
| C | -3.162675 | -1.701972 | -1.193751 | H | -7.31479 | -1.073588 | -0.557534 |
| C | -4.660327 | -1.385662 | -1.342212 | H | -6.811532 | 0.385029 | -1.397763 |
| C | -5.251758 | -0.529057 | -0.213007 | H | -8.362363 | 0.949072 | 0.487242 |
| O | -0.979029 | -0.780466 | 1.08527 | H | -7.270443 | 0.169507 | 1.643482 |
| C | -6.721796 | -0.154714 | -0.44418 | H | -6.761123 | 1.639097 | 0.798636 |
| C | -7.313357 | 0.698091 | 0.682967 | H | -0.199335 | 4.194051 | -1.329511 |
| C | 0.032017 | 4.194344 | -0.262284 | H | 0.372675 | 5.201801 | 0.006549 |
| C | -1.218969 | 3.844947 | 0.565858 | H | -1.02471 | 3.964923 | 1.633203 |
| C | -1.641789 | 2.413072 | 0.291595 | H | -2.044411 | 4.5152 | 0.298451 |
| O | -2.138323 | 1.78824 | 1.368787 | H | -2.196087 | 0.830438 | 1.154417 |
| O | -1.507235 | 1.865986 | -0.791012 | H | 3.361308 | -2.355617 | 2.430888 |
| C | 3.988171 | -1.688299 | 1.831743 | H | 4.927227 | -2.195689 | 1.599705 |
| H | 1.141292 | -2.434666 | 1.807908 | H | 4.209593 | -0.797572 | 2.429063 |

**Table S55.** The Cartesian coordinates of the lowest energy conformers (Conf. C) for **10b**.

| **Conf. A** | **X axis** | **Y axis** | **Z axis** | **Conf. A** | **X axis** | **Y axis** | **Z axis** |
| --- | --- | --- | --- | --- | --- | --- | --- |
| C | 1.655508 | -0.714736 | 0.671336 | H | 1.516534 | -0.060805 | 1.534428 |
| C | 3.022021 | -1.423121 | 0.725942 | H | 1.580145 | -0.530192 | -1.494838 |
| C | 4.166557 | -0.669015 | 0.000182 | H | 0.580947 | 0.628301 | -0.645346 |
| C | 3.944669 | 0.730854 | -0.434745 | H | 5.876172 | 1.074032 | -1.302237 |
| C | 2.677907 | 1.125831 | -0.704204 | H | 5.738941 | 1.62102 | 0.363146 |
| C | 1.546519 | 0.122074 | -0.609912 | H | 4.995772 | 2.568199 | -0.936454 |
| C | 5.198184 | 1.554247 | -0.588319 | H | 3.094041 | 3.193309 | -1.280901 |
| O | 1.383986 | 2.807619 | 1.079117 | H | 1.728288 | 2.451679 | -2.099811 |
| C | 1.312977 | 3.130314 | -0.095841 | H | -1.225429 | -3.240651 | -0.960422 |
| C | 2.252913 | 2.505548 | -1.137582 | H | -0.593344 | -1.886816 | -1.840274 |
| C | 0.67841 | -1.913966 | 0.695577 | H | -2.900961 | -1.929339 | -2.449169 |
| C | 1.473555 | -2.998026 | -0.03027 | H | -2.765751 | -0.599837 | -1.308323 |
| O | 2.792741 | -2.675014 | -0.010117 | H | -3.603754 | -2.188383 | 0.522342 |
| O | 5.252092 | -1.224733 | -0.111706 | H | -3.761637 | -3.441445 | -0.695348 |
| O | 1.082078 | -4.023486 | -0.536921 | H | -5.957514 | -2.531965 | -0.35908 |
| C | -0.746553 | -1.560995 | 0.235822 | H | -5.414965 | -2.026412 | -1.950961 |
| C | -1.301643 | -2.147872 | -1.039203 | H | -4.846218 | 0.257611 | -1.009594 |
| C | -2.720412 | -1.690998 | -1.392877 | H | -6.545206 | -0.164953 | -0.928072 |
| C | -3.813537 | -2.354404 | -0.540906 | H | -6.299252 | -0.784037 | 1.481852 |
| C | -5.239019 | -1.879533 | -0.875526 | H | -4.59485 | -0.365508 | 1.431118 |
| C | -5.55841 | -0.417278 | -0.51411 | H | -5.950274 | 1.513272 | 2.39322 |
| O | -1.363335 | -0.768873 | 0.936199 | H | -5.156643 | 2.017675 | 0.893793 |
| C | -5.571367 | -0.120878 | 0.992392 | H | -6.884083 | 1.621706 | 0.89161 |
| C | -5.910548 | 1.339257 | 1.311559 | H | 0.117273 | 4.068101 | -1.641189 |
| C | 0.262372 | 4.13639 | -0.560922 | H | 0.634517 | 5.145588 | -0.342276 |
| C | -1.065573 | 3.90707 | 0.182114 | H | -1.827641 | 4.600747 | -0.192323 |
| C | -1.547432 | 2.485772 | -0.045112 | H | -0.951783 | 4.082935 | 1.253072 |
| O | -2.157377 | 1.949406 | 1.021019 | H | -2.27614 | 0.988478 | 0.841541 |
| O | -1.367181 | 1.870587 | -1.084215 | H | 2.73079 | -2.327012 | 2.685517 |
| C | 3.497213 | -1.771088 | 2.136841 | H | 4.403584 | -2.378086 | 2.077166 |
| H | 0.565053 | -2.257445 | 1.732653 | H | 3.72351 | -0.856498 | 2.694728 |

**Table S56.** The Cartesian coordinates of the lowest energy conformers (Conf. D) for **10b**.

| **Conf. A** | **X axis** | **Y axis** | **Z axis** | **Conf. A** | **X axis** | **Y axis** | **Z axis** |
| --- | --- | --- | --- | --- | --- | --- | --- |
| C | 1.852296 | -0.700102 | 0.652452 | H | 1.778281 | -0.065368 | 1.537547 |
| C | 3.234238 | -1.374187 | 0.565986 | H | 0.62821 | 0.637017 | -0.533444 |
| C | 4.288622 | -0.575826 | -0.24422 | H | 1.576127 | -0.477466 | -1.494149 |
| C | 3.993502 | 0.826874 | -0.623221 | H | 5.830377 | 1.241742 | -1.650535 |
| C | 2.69801 | 1.193246 | -0.76755 | H | 4.948796 | 2.702959 | -1.171477 |
| C | 1.605687 | 0.156901 | -0.596019 | H | 5.829026 | 1.748049 | 0.033225 |
| C | 5.207037 | 1.687438 | -0.867706 | H | 1.591254 | 2.52747 | -2.033793 |
| O | 1.529384 | 2.790596 | 1.171528 | H | 3.007613 | 3.285288 | -1.322617 |
| C | 1.34461 | 3.142776 | 0.017603 | H | -1.127846 | -3.270833 | -0.737296 |
| C | 2.20073 | 2.571105 | -1.122471 | H | -0.58753 | -1.905835 | -1.660056 |
| C | 0.910453 | -1.923866 | 0.741766 | H | -2.712418 | -0.640954 | -0.961254 |
| C | 1.659723 | -2.973074 | -0.077895 | H | -2.941848 | -1.994086 | -2.060114 |
| O | 2.967232 | -2.616795 | -0.173337 | H | -3.416677 | -2.12743 | 0.965816 |
| O | 5.372463 | -1.100216 | -0.467846 | H | -3.584514 | -3.474675 | -0.15215 |
| O | 1.247001 | -3.998038 | -0.568357 | H | -5.399051 | -2.233404 | -1.376147 |
| C | -0.558665 | -1.595911 | 0.423704 | H | -5.789357 | -2.614963 | 0.294026 |
| C | -1.219868 | -2.178124 | -0.80141 | H | -5.16967 | -0.260526 | 0.965417 |
| C | -2.668307 | -1.733351 | -1.029153 | H | -4.872694 | 0.132683 | -0.721999 |
| C | -3.677488 | -2.382388 | -0.069283 | H | -7.555692 | -0.818576 | 0.410713 |
| C | -5.140107 | -1.989214 | -0.334813 | H | -7.254931 | -0.439309 | -1.27822 |
| C | -5.468803 | -0.51396 | -0.06317 | H | -8.350805 | 1.496222 | -0.124461 |
| O | -1.121665 | -0.826823 | 1.191451 | H | -7.025071 | 1.571129 | 1.047621 |
| C | -6.954528 | -0.180488 | -0.252754 | H | -6.72356 | 1.952537 | -0.654339 |
| C | -7.283474 | 1.291198 | 0.018681 | H | -0.009353 | 4.088337 | -1.385563 |
| C | 0.233272 | 4.133629 | -0.321647 | H | 0.601701 | 5.145991 | -0.111287 |
| C | -1.014466 | 3.853486 | 0.534106 | H | -0.804554 | 4.003621 | 1.594415 |
| C | -1.486695 | 2.427776 | 0.312367 | H | -1.822273 | 4.539048 | 0.251899 |
| O | -1.98457 | 1.851166 | 1.413574 | H | -2.102729 | 0.893012 | 1.219019 |
| O | -1.391475 | 1.845459 | -0.756956 | H | 3.14536 | -2.321865 | 2.524546 |
| C | 3.845398 | -1.737609 | 1.919513 | H | 4.75547 | -2.321692 | 1.764167 |
| H | 0.902166 | -2.290141 | 1.777261 | H | 4.102176 | -0.828756 | 2.473584 |

**Table S57.** The Cartesian coordinates of the lowest energy conformers (Conf. E) for **10b**.

| **Conf. A** | **X axis** | **Y axis** | **Z axis** | **Conf. A** | **X axis** | **Y axis** | **Z axis** |
| --- | --- | --- | --- | --- | --- | --- | --- |
| C | 2.013066 | -0.664995 | 0.678347 | H | 2.17571 | 0.016498 | 1.515634 |
| C | 3.323061 | -1.361629 | 0.265679 | H | 0.516565 | 0.623004 | -0.212344 |
| C | 4.126707 | -0.618517 | -0.833246 | H | 1.17644 | -0.553736 | -1.326706 |
| C | 3.737129 | 0.763951 | -1.200451 | H | 5.217187 | 1.1114 | -2.715037 |
| C | 2.448299 | 1.137549 | -1.019452 | H | 4.512578 | 2.602566 | -2.066378 |
| C | 1.442977 | 0.127234 | -0.505167 | H | 5.688194 | 1.676851 | -1.118239 |
| C | 4.839419 | 1.594477 | -1.80719 | H | 1.04042 | 2.404919 | -2.027663 |
| O | 1.826432 | 2.863208 | 1.057148 | H | 2.590475 | 3.18849 | -1.765274 |
| C | 1.342113 | 3.147454 | -0.026507 | H | -1.259051 | -3.278742 | 0.336444 |
| C | 1.869135 | 2.49743 | -1.314464 | H | -0.961657 | -2.032137 | -0.831538 |
| C | 1.130047 | -1.86982 | 1.078946 | H | -3.123195 | -1.869949 | 1.356944 |
| C | 1.633952 | -2.968182 | 0.144335 | H | -2.799532 | -0.618614 | 0.165977 |
| O | 2.869062 | -2.634855 | -0.313335 | H | -3.400802 | -2.21843 | -1.682324 |
| O | 5.117402 | -1.165074 | -1.301968 | H | -3.644641 | -3.496694 | -0.501283 |
| O | 1.104559 | -4.009583 | -0.166147 | H | -5.812834 | -2.669347 | -1.137791 |
| C | -0.371451 | -1.544353 | 1.146797 | H | -5.530899 | -2.194119 | 0.53004 |
| C | -1.339309 | -2.192392 | 0.189686 | H | -4.954996 | 0.13421 | -0.227858 |
| C | -2.781302 | -1.702111 | 0.327233 | H | -5.16312 | -0.357481 | -1.902596 |
| C | -3.729352 | -2.410916 | -0.650181 | H | -7.363979 | -0.407964 | 0.234677 |
| C | -5.205848 | -2.006996 | -0.504371 | H | -7.575948 | -0.88778 | -1.442072 |
| C | -5.516537 | -0.550689 | -0.878294 | H | -8.396164 | 1.452884 | -1.088121 |
| O | -0.71732 | -0.735784 | 1.998179 | H | -6.798518 | 1.943747 | -0.501937 |
| C | -7.009906 | -0.209929 | -0.787192 | H | -7.010722 | 1.460876 | -2.191554 |
| C | -7.322961 | 1.242206 | -1.162714 | H | -0.335105 | 4.027678 | -1.080275 |
| C | 0.175938 | 4.128974 | -0.1204 | H | 0.581311 | 5.147722 | -0.069245 |
| C | -0.80518 | 3.908116 | 1.044425 | H | -0.327303 | 4.113198 | 2.003821 |
| C | -1.312285 | 2.477152 | 1.031184 | H | -1.661559 | 4.5855 | 0.944735 |
| O | -1.500047 | 1.964332 | 2.253589 | H | -1.661634 | 0.998126 | 2.150562 |
| O | -1.497963 | 1.838778 | 0.006593 | H | 3.754217 | -2.21464 | 2.222914 |
| C | 4.269403 | -1.66824 | 1.426847 | H | 5.106425 | -2.270272 | 1.065622 |
| H | 1.399337 | -2.182948 | 2.096693 | H | 4.663103 | -0.73764 | 1.848588 |

**Table S58.** The Cartesian coordinates of the lowest energy conformers (Conf. F) for **10b**.

| **Conf. A** | **X axis** | **Y axis** | **Z axis** | **Conf. A** | **X axis** | **Y axis** | **Z axis** |
| --- | --- | --- | --- | --- | --- | --- | --- |
| C | 1.853436 | -0.813106 | 0.744975 | H | 2.044965 | -0.13294 | 1.57719 |
| C | 3.031136 | -1.783825 | 0.538216 | H | 0.788288 | 0.699254 | -0.383219 |
| C | 4.106664 | -1.284129 | -0.46148 | H | 1.330461 | -0.647948 | -1.359902 |
| C | 4.068796 | 0.127626 | -0.912944 | H | 5.788575 | 0.068334 | -2.193831 |
| C | 2.874897 | 0.76616 | -0.921699 | H | 5.316387 | 1.712238 | -1.728458 |
| C | 1.62162 | 0.010508 | -0.528404 | H | 6.132559 | 0.648996 | -0.570251 |
| C | 5.391568 | 0.681836 | -1.377774 | H | 1.903822 | 2.247548 | -2.134283 |
| O | 2.38331 | 2.68447 | 1.015835 | H | 3.541367 | 2.702192 | -1.688706 |
| C | 2.108741 | 3.009233 | -0.128189 | H | -1.806249 | -2.708101 | -0.013992 |
| C | 2.639115 | 2.200036 | -1.321282 | H | -1.096095 | -1.594171 | -1.13777 |
| C | 0.693421 | -1.788244 | 1.054036 | H | -3.452519 | -0.906433 | 0.716382 |
| C | 1.073847 | -3.018021 | 0.231468 | H | -2.729575 | 0.202017 | -0.43988 |
| O | 2.400266 | -2.970368 | -0.05842 | H | -3.335933 | -1.373179 | -2.317063 |
| O | 5.009942 | -2.048437 | -0.777015 | H | -4.112301 | -2.446443 | -1.162291 |
| O | 0.382867 | -3.945696 | -0.119602 | H | -4.953474 | 0.455182 | -1.716604 |
| C | -0.701744 | -1.160417 | 0.900387 | H | -5.705173 | -0.967038 | -2.423397 |
| C | -1.638805 | -1.634674 | -0.181097 | H | -6.357392 | -1.759199 | -0.11919 |
| C | -2.953126 | -0.856759 | -0.258105 | H | -5.675868 | -0.292898 | 0.571019 |
| C | -3.878231 | -1.390048 | -1.361055 | H | -7.964113 | -0.373309 | -1.464628 |
| C | -5.188824 | -0.602221 | -1.524148 | H | -7.272989 | 1.092411 | -0.785828 |
| C | -6.151799 | -0.699132 | -0.33234 | H | -9.377999 | 0.471526 | 0.423274 |
| O | -0.986913 | -0.26325 | 1.682831 | H | -8.691834 | -1.106065 | 0.844457 |
| C | -7.47883 | 0.034904 | -0.566523 | H | -7.996466 | 0.371619 | 1.527036 |
| C | -8.440876 | -0.061356 | 0.622131 | H | 0.809508 | 4.17392 | -1.414965 |
| C | 1.200779 | 4.207743 | -0.395916 | H | 1.803291 | 5.120604 | -0.304417 |
| C | 0.052214 | 4.247339 | 0.627572 | H | 0.434019 | 4.392029 | 1.639617 |
| C | -0.7398 | 2.953427 | 0.569856 | H | -0.621383 | 5.082294 | 0.401106 |
| O | -1.195599 | 2.551257 | 1.762694 | H | -1.544347 | 1.636127 | 1.656608 |
| O | -0.91606 | 2.317925 | -0.458168 | H | 3.019568 | -2.591609 | 2.56029 |
| C | 3.735505 | -2.20668 | 1.827661 | H | 4.469357 | -2.984491 | 1.604096 |
| H | 0.755893 | -2.089622 | 2.108336 | H | 4.254929 | -1.351253 | 2.271956 |

**Table S59.** The Cartesian coordinates of the lowest energy conformers (Conf. G) for **10b**.

| **Conf. A** | **X axis** | **Y axis** | **Z axis** | **Conf. A** | **X axis** | **Y axis** | **Z axis** |
| --- | --- | --- | --- | --- | --- | --- | --- |
| C | 1.81786 | -0.920172 | 0.757264 | H | 2.301717 | -0.260572 | 1.480332 |
| C | 2.711985 | -2.128101 | 0.420637 | H | 0.877258 | 0.732374 | -0.28121 |
| C | 3.650513 | -1.913128 | -0.795443 | H | 0.934427 | -0.742349 | -1.221799 |
| C | 3.806789 | -0.5482 | -1.352448 | H | 5.166562 | 0.700879 | -2.502903 |
| C | 2.786061 | 0.328026 | -1.198615 | H | 5.959315 | -0.480053 | -1.447136 |
| C | 1.510114 | -0.124005 | -0.517494 | H | 5.183257 | -1.019335 | -2.929746 |
| C | 5.094622 | -0.310243 | -2.099388 | H | 1.911328 | 1.913526 | -2.349922 |
| O | 3.064445 | 2.408798 | 0.612199 | H | 3.660313 | 2.038085 | -2.243099 |
| C | 2.640616 | 2.721166 | -0.488892 | H | -1.556502 | -1.148049 | -0.467914 |
| C | 2.764392 | 1.756762 | -1.678025 | H | -2.225898 | -2.030436 | 0.866111 |
| C | 0.568945 | -1.610885 | 1.353053 | H | -3.317899 | 0.103978 | 1.726541 |
| C | 0.52442 | -2.926809 | 0.578209 | H | -2.624073 | 0.986475 | 0.374079 |
| O | 1.749923 | -3.175694 | 0.047201 | H | -4.593782 | -1.354161 | 0.116337 |
| O | 4.304661 | -2.864934 | -1.202877 | H | -3.915086 | -0.45676 | -1.234664 |
| O | -0.394421 | -3.699498 | 0.436881 | H | -5.703108 | 0.731469 | 0.958531 |
| C | -0.674332 | -0.707348 | 1.408833 | H | -4.952962 | 1.660112 | -0.330916 |
| C | -1.888543 | -1.025997 | 0.57424 | H | -7.243383 | 1.254919 | -0.957325 |
| C | -3.012063 | 0.006167 | 0.676899 | H | -6.220633 | 0.325493 | -2.042297 |
| C | -4.227276 | -0.363085 | -0.183705 | H | -6.717179 | -1.758424 | -0.731835 |
| C | -5.359865 | 0.668944 | -0.084971 | H | -7.678084 | -0.821166 | 0.401779 |
| C | -6.564683 | 0.39084 | -0.99898 | H | -9.146505 | -1.972985 | -1.273761 |
| O | -0.608572 | 0.268529 | 2.144784 | H | -9.281555 | -0.216057 | -1.456641 |
| C | -7.362489 | -0.873915 | -0.650362 | H | -8.305979 | -1.152056 | -2.598902 |
| C | -8.593048 | -1.065975 | -1.543386 | H | 2.716498 | 4.83626 | -0.811082 |
| C | 1.94442 | 4.064219 | -0.698748 | H | 1.356568 | 4.052443 | -1.619145 |
| C | 1.054406 | 4.400521 | 0.510767 | H | 1.651432 | 4.520682 | 1.416301 |
| C | 0.032941 | 3.297927 | 0.724496 | H | 0.522517 | 5.342601 | 0.3325 |
| O | -0.242267 | 3.064631 | 2.013733 | H | -0.775102 | 2.237355 | 2.060298 |
| O | -0.465638 | 2.657565 | -0.18828 | H | 2.931268 | -2.834112 | 2.469292 |
| C | 3.553469 | -2.638701 | 1.590515 | H | 4.058686 | -3.562654 | 1.299889 |
| H | 0.775725 | -1.87471 | 2.399009 | H | 4.310294 | -1.895985 | 1.863432 |

**Table S60.** The Cartesian coordinates of the lowest energy conformers (Conf. H) for **10b**.

| **Conf. A** | **X axis** | **Y axis** | **Z axis** | **Conf. A** | **X axis** | **Y axis** | **Z axis** |
| --- | --- | --- | --- | --- | --- | --- | --- |
| C | 1.558394 | -1.010292 | 0.555481 | H | 1.6031 | -0.460984 | 1.498454 |
| C | 2.567418 | -2.175344 | 0.553408 | H | 1.672724 | -0.540577 | -1.566315 |
| C | 3.971629 | -1.824944 | -0.000382 | H | 1.16868 | 0.794871 | -0.562153 |
| C | 4.303444 | -0.417248 | -0.308273 | H | 6.269037 | -0.710432 | -1.1267 |
| C | 3.291768 | 0.441447 | -0.579995 | H | 6.256977 | -0.376536 | 0.598755 |
| C | 1.858062 | -0.045921 | -0.60053 | H | 5.98562 | 0.942073 | -0.550832 |
| C | 5.779529 | -0.110476 | -0.351376 | H | 4.491741 | 2.200513 | -1.088158 |
| O | 2.751562 | 2.391214 | 1.324215 | H | 2.914336 | 2.123459 | -1.854308 |
| C | 2.877895 | 2.797175 | 0.180535 | H | -2.343845 | -2.619371 | -0.064781 |
| C | 3.450575 | 1.899564 | -0.923647 | H | -1.91017 | -1.945258 | -1.601746 |
| C | 0.227095 | -1.767244 | 0.383122 | H | -4.205552 | -1.334109 | -1.277219 |
| C | 0.636123 | -2.944335 | -0.509331 | H | -3.299811 | 0.150635 | -1.016643 |
| O | 1.973198 | -3.138099 | -0.389839 | H | -3.408745 | -0.251803 | 1.479604 |
| O | 4.797998 | -2.7249 | -0.098576 | H | -4.383199 | -1.687269 | 1.190966 |
| O | -0.044448 | -3.651656 | -1.214034 | H | -5.174991 | 1.176401 | 0.412291 |
| C | -1.017843 | -0.947019 | 0.018011 | H | -5.707967 | 0.340278 | 1.862445 |
| C | -2.209059 | -1.661122 | -0.582899 | H | -6.360175 | -0.521431 | -1.010633 |
| C | -3.50415 | -0.841185 | -0.594455 | H | -6.823419 | -1.41359 | 0.43186 |
| C | -4.145189 | -0.689258 | 0.793755 | H | -8.137524 | 0.573048 | 1.231877 |
| C | -5.413002 | 0.180644 | 0.815531 | H | -7.663668 | 1.468138 | -0.203737 |
| C | -6.611061 | -0.402372 | 0.052644 | H | -9.962232 | 0.510396 | -0.483449 |
| O | -1.027865 | 0.246877 | 0.279795 | H | -8.857266 | -0.219721 | -1.659404 |
| C | -7.877267 | 0.45649 | 0.170067 | H | -9.333525 | -1.123309 | -0.213799 |
| C | -9.075032 | -0.124908 | -0.588237 | H | 2.458145 | 4.351429 | -1.277041 |
| C | 2.425627 | 4.207665 | -0.19501 | H | 3.119272 | 4.924004 | 0.261524 |
| C | 1.005486 | 4.464686 | 0.345117 | H | 0.656004 | 5.45316 | 0.024315 |
| C | 0.049066 | 3.410661 | -0.182558 | H | 0.996864 | 4.446141 | 1.436498 |
| O | -0.892048 | 3.056094 | 0.704947 | H | -1.342841 | 2.257812 | 0.351639 |
| O | 0.145648 | 2.900863 | -1.286285 | H | 1.780531 | -3.173867 | 2.324832 |
| C | 2.743364 | -2.866029 | 1.906112 | H | 3.376699 | -3.747634 | 1.7849 |
| H | -0.048098 | -2.228527 | 1.346308 | H | 3.223692 | -2.184903 | 2.616334 |

**References**

Frisvad JC, Yilmaz N, Thrane U, Rasmussen KB, Houbraken J, Samson RA. 2013. *Talaromyces atroroseus*, a new species efficiently producing industrially relevant red pigments. PLOS One. 8(12):e84102. doi: 10.1371/journal.pone.0084102.

He Y, Cox R. 2016. The molecular steps of citrinin biosynthesis in fungi. Chem Sci. 7(3):2119–2127. doi: 10.1039/c5sc04027b.

Hou C, Wang Y, Meng X, Lu L, Fu P, Zhu W, Wang Y. 2025. Bioactive hydrogenated azaphilones from acid-tolerant fungus *Penicillium purpureum* OUCMDZ-019. J Antibiot. 78(5):281–287. doi: 10.1038/s41429-025-00818-7.

Wang H, Wang Y, Wang W, Fu P, Liu P, Zhu W. 2011. Anti-influenza virus polyketides from the acid-tolerant fungus *Penicillium purpurogenum* JS03-21. J Nat Prod. 74(9):2014–2018. doi: 10.1021/np2004769.

Wang H, Wang Y, Liu P, Wang W, Fan Y, Zhu W. 2013. Purpurides B and C, two new sesquiterpene esters from the aciduric fungus *Penicillium purpurogenum* JS03-21. Chem Biodiver. 10(7):1185–1192. doi: 10.1002/cbdv.201200175.
